# Supplementary material for: Presynaptic NMDARs on spinal nociceptor terminals state-dependently modulate synaptic transmission and pain
Source: Nat Commun. 2022 Feb 7;13:728. doi: 10.1038/s41467-022-28429-y (PMC8821657; doi:10.1038/s41467-022-28429-y)
Supplement: Supplementary file 1 — Supplementary Information [file 41467_2022_28429_MOESM1_ESM.pdf]

SUPPLEMENTARY INFORMATION

Presynaptic NMDARs on spinal nociceptor terminals state-dependently modulate synaptic transmission and pain

Xie et al

Supplementary figure 1

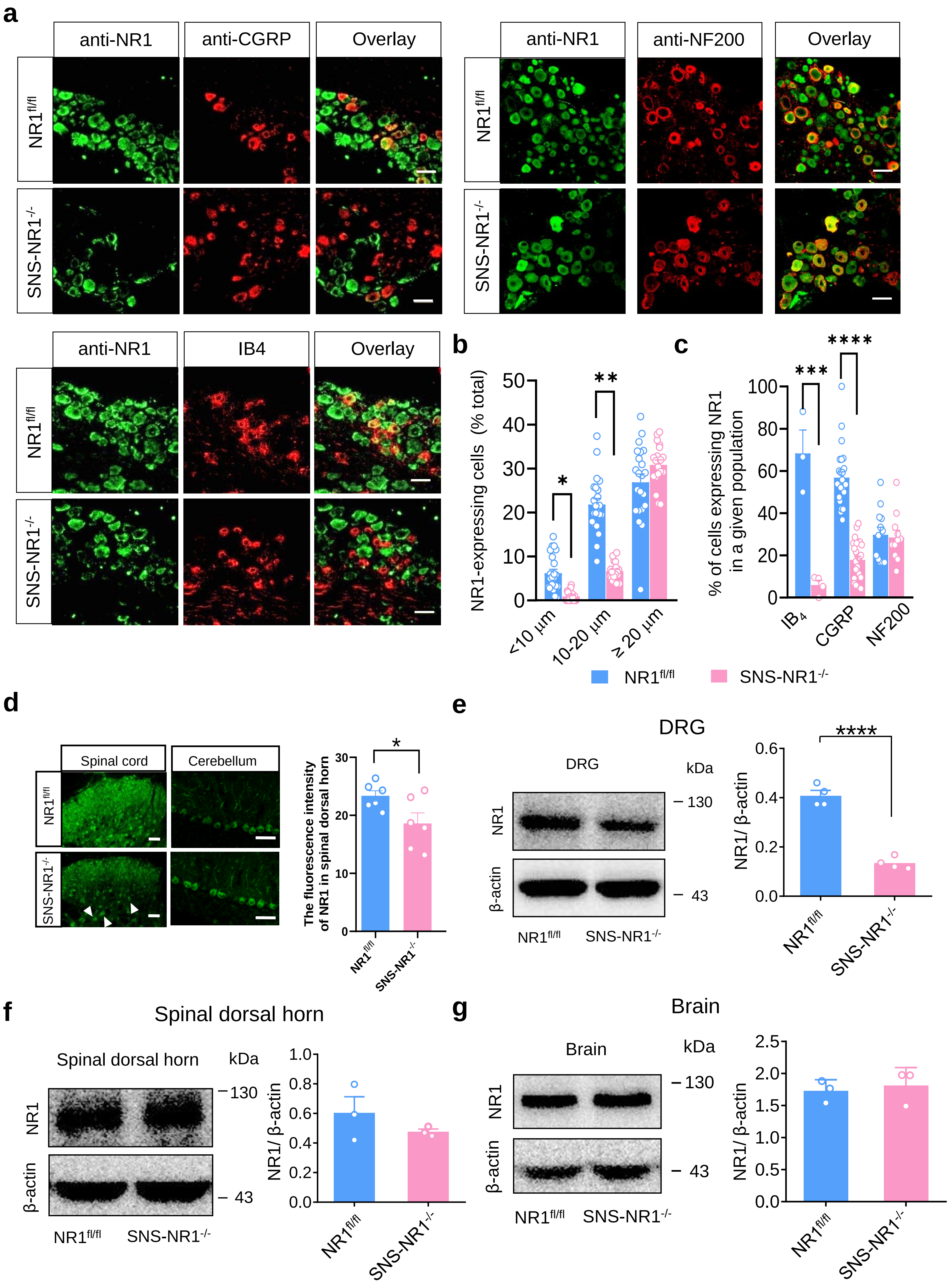

Supplementary Figure 1: Morphological confirmation of conditional deletion of NR1 specifically in nociceptive neurons of the dorsal root ganglia (DRG). (a-c) Typical examples (a) and quantitative summary (b, c) showing that in SNS-NR1$^{–/–}$ mice, NR1 immunoreactivity is abrogated from small- to medium-sized nociceptors (Isolectin B_4- or CGRP-positive neurons), but entirely preserved in large diameter neurons (NF200-positive neurons). n = 22 DRG sections for NR1$^{fl/fl}$, n = 19 DRG sections for SNS-NR1$^{-/-}$, *P < 0.05, *P < 0.01, ***P < 0.001, ****P < 0.0001 by Kruskal-Wallis H test. (d) Typical examples of anti-NR1 immunostaining in the spinal dorsal horn and brain of SNS-NR1$^{–/–}$ mice and their NR1$^{fl/fl}$ littermates. Spinal dorsal horns of SNS-NR1$^{–/–}$ mice showed a loss of anti-NR1 immunoreactivity in the superficial neuropil (quantitative summary shown in right panel), but distinct preservation of signals in cell bodies. Intact immunoreactivity of NR1 were seen in the brain of SNS-NR1$^{–/–}$ mice, i.e. cerebellar Purkinje neurons. n = 6, *P < 0.05 by unpaired t test. (e-g) Western blot analysis with anti-NR1 antibody confirmed that SNS-NR1$^{-/-}$ mice show a DRG-specific loss of NR1 (e) while retaining expression in the spinal dorsal horn (f) and brain (g). Shown are typical example blots in left panels and quantitative summary in right panels. n = 3-4, ****P < 0.0001 by unpaired t test. Scale bar represents 50 μm in all panels. Data are represented as mean ± S.E.M. See Supplemental Table 2 for detailed statistical information. DRG: dorsal root ganglia.

Supplementary figure 2

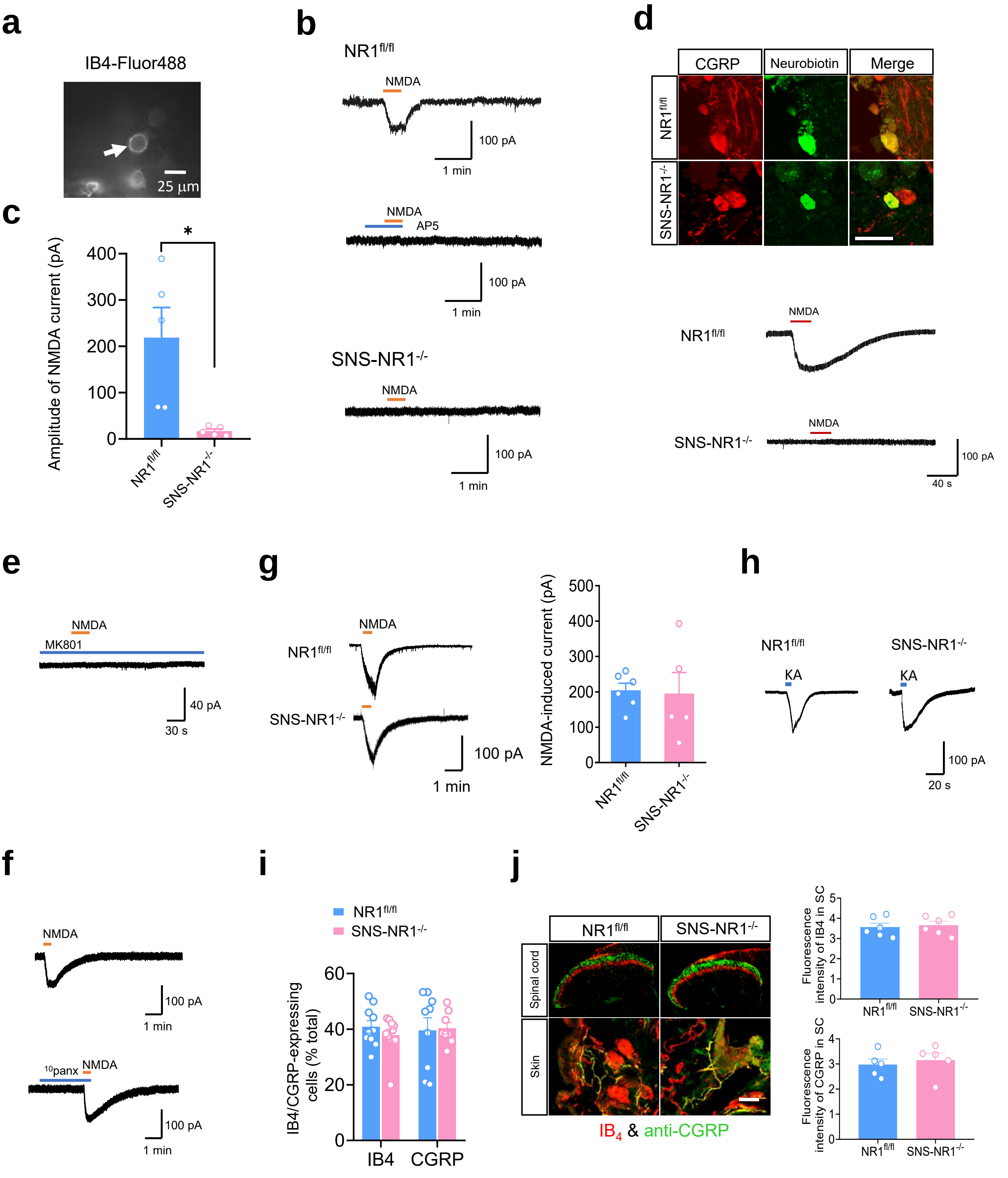

Supplementary Figure 2: Functional confirmation of conditional deletion of NR1 specifically in nociceptors and normal development of the sensory circuitry in SNS-NR1$^{-/-}$ mice. (a-c) NMDA induced an inward current in Fluor 488-conjugated isolectin B4 (IB4)-identified non-peptidergic nociceptive DRG neurons (a, arrow) of NR1$^{fl/fl}$ mice (b, upper panels), which was completely abolished in the presence of AP5 (b, middle panels) and SNS-NR1$^{-/-}$ mice (b, lower panels, and quantitative summary shown in c) (n = 5). **P* < 0.05 by unpaired *t* test with Welch's correction. (d) NMDA-induced currents were lost in CGRP-positive peptidergic DRG neurons in SNS-NR1$^{-/-}$ mice. Upper panels showing double immunofluorescence staining of CGRP and Neurobiotin in the patched DRG neurons. Lower panels showing the NMDA-induced inward current in CGRP-positive peptidergic DRG neurons derived from NR1$^{fl/fl}$ mice and SNS-NR1$^{-/-}$ mice shown above. (e, f) NMDA-induced inward current was blocked by MK-801 (e), an NMDAR channel blocker, but not ^{10}panx (f), a Panx1-blocking peptide. (g) NMDA-induced inward current in spinal neurons were comparable in NR1$^{fl/fl}$ mice and SNS-NR1$^{-/-}$ mice. Quantification was shown in the right panel (n = 5-6). *P* > 0.05 by unpaired *t* test. (h) Nociceptor-specific deletion of NR1 did not affect kainite receptor function, as characterized by no difference of kainic acid (KA)-induced current in NR1$^{fl/fl}$ mice and SNS-NR1$^{-/-}$ mice. (i) Normal development of IB4-labelled non-peptidergic and CGRP-expressing peptidergic nociceptive DRG neurons in adult SNS-NR1$^{-/-}$ mice, as compared to their NR1$^{fl/fl}$ littermates (n = 8-10). *P* > 0.05 by one way-ANOVA. (j) Adult NR1$^{fl/fl}$ mice and SNS-NR1$^{-/-}$ mice show similar patterns targeting of nociceptors in the spinal cord (upper panels) and the skin (lower panels) as shown by binding to Isolectin-B_4 (red) and immunoreactivity for CGRP (green) (n = 6). Shown are quantitative summary for spinal

cord in right panels. *P* > 0.05 by unpaired *t* test. Scale bars represent 50 μm in (d) and (j). Data are represented as mean ± S.E.M. See Supplemental Table 2 for detailed statistical information. SC: spinal cord.

Supplementary figure 3

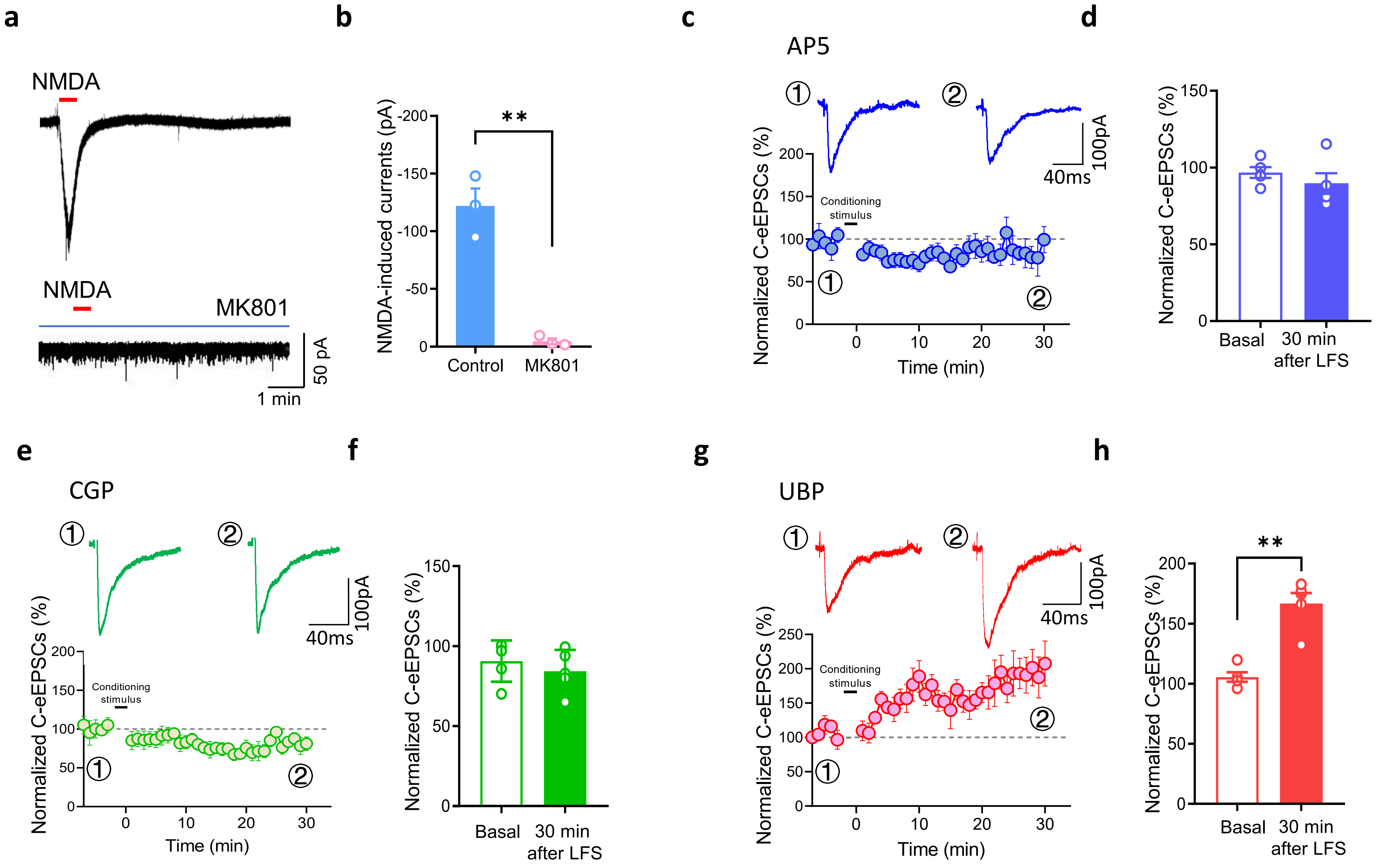

Supplementary Figure 3: Pharmacological assessment of PreNMDARs involved in the pre-LTP induced by low-frequency conditioning stimulus. (a, b) NMDA-induced inward current in spinal neurons was blocked by dialyzing NMDARs blocker, MK-801 (1 mM) into recorded neurons via the patch pipette. Typical traces and quantitative summary in the absence and presence of MK-801 are shown in (a) and (b), respectively. $^{**}P < 0.01$ by unpaired *t* test. (c-h) Time course and quantitative summary showing the effect of bath application of AP5 (50 μm, c, d), CGP78608 (1 μM, e, f) and UBP310 (10 μM, g, f) on pre-LTP induced by low-frequency conditioning stimulus. n = 5, $^{**}P < 0.01$ by paired *t* test. Data are represented as mean ± S.E.M. See Supplemental Table 2 for detailed statistical information. CGP, CGP78608; UBP, UBP310.

Supplementary figure 4

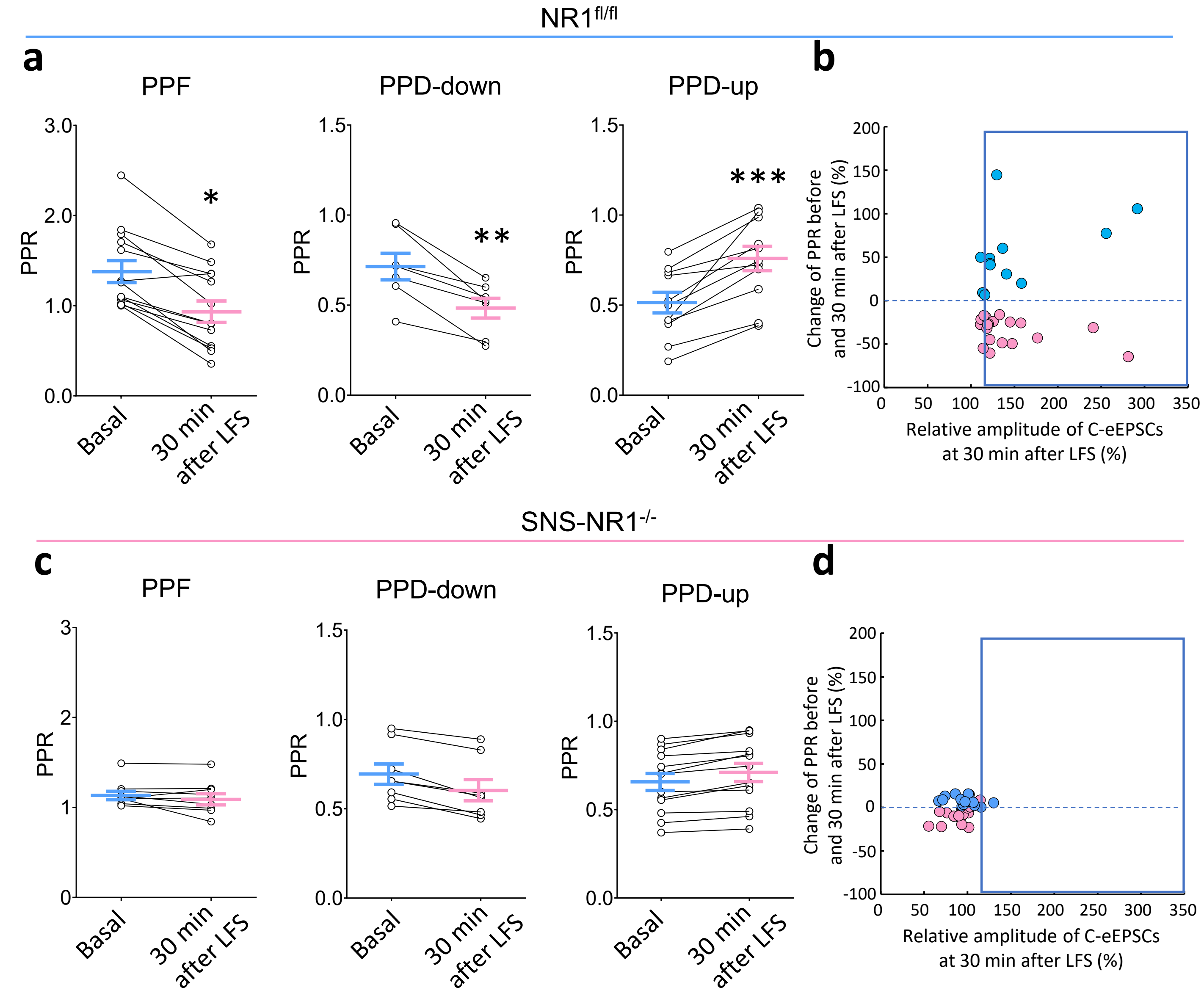

Supplementary Figure 4: The absolute changes of paired-pulse ratio (PPR) of C-eEPSCs prior to and 30 min following low frequency conditioning stimulus (LFS). (a) The absolute changes of PPF （paired-pulse facilitation) or PPD (paired-pulse depression) of C-eEPSCs prior to and 30 min following LFS in $NR1^{fl/fl}$ mice (a) and SNS-$NR1^{-/-}$ mice (c). **P* < 0.05 by Mann Whitney *U* test for left panel, ***P* < 0.01, ****P* < 0.001 by paired t test for middle and right panels in (a). (b, d) The changes of PPR following LFS was plotted to the relative magnitude of LTP in $NR1^{fl/fl}$ mice (b) and SNS-$NR1^{-/-}$ mice (d). Note that in $NR1^{fl/fl}$ mice, higher magnitudes of LTP were consistently linked with a big change in PPR (decrease / increase), which is indicative of presynaptic mechanisms. Data are represented as mean ± S.E.M. See Supplemental Table 2 for detailed statistical information.

Supplementary figure 5

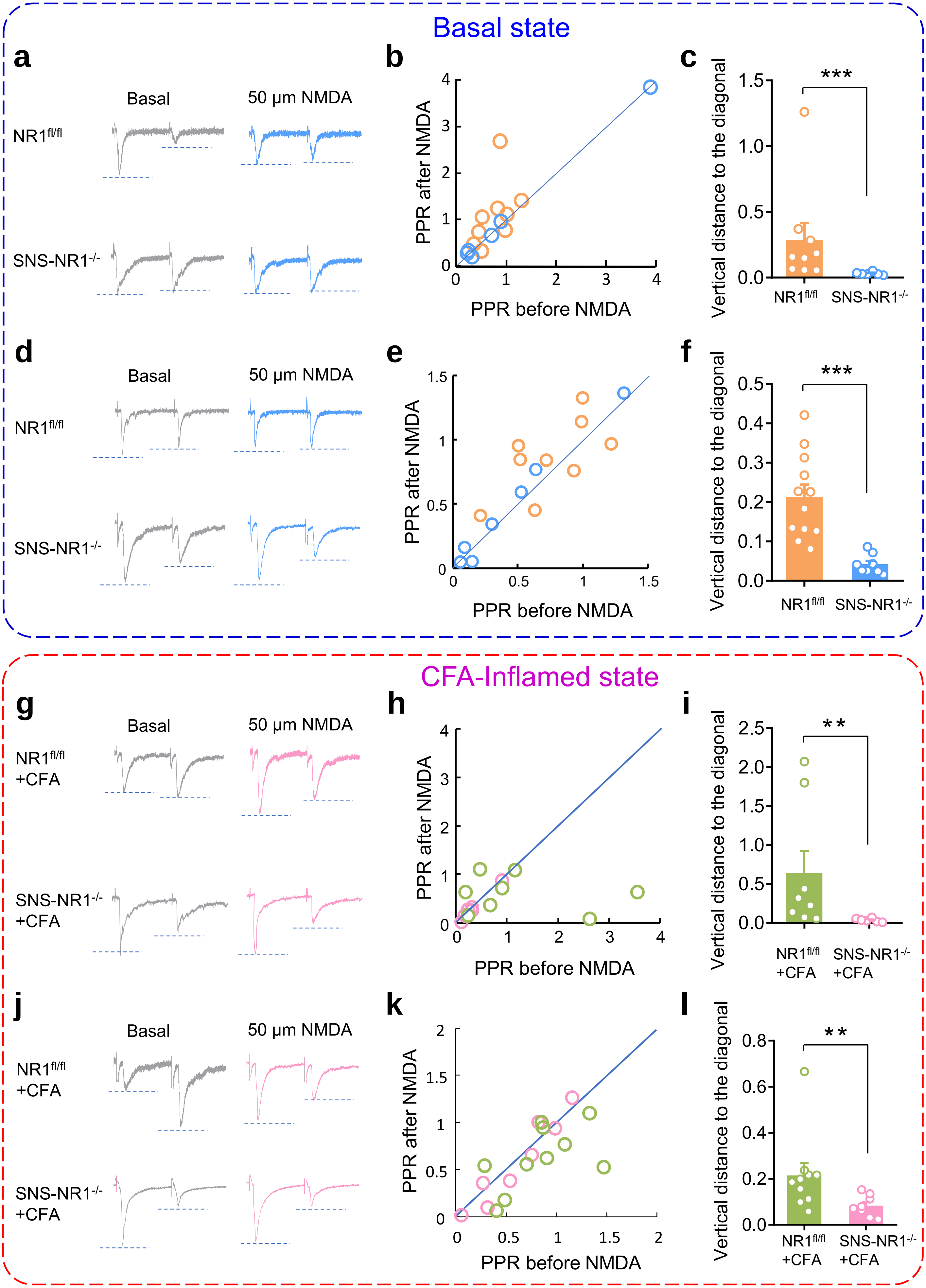

Supplementary Figure 5: PPR changes associated with NMDA application. (a, d) Traces of typical recordings showing paired-pulse facilitation (PPF) or paired-pulse depression (PPD) of C-eEPSCs induced by pairs of pulses with an interval of 110 ms prior to (grey traces) and following acute (a) or long-term exposure (d) to NMDA (blue traces) in basal state. (b, e) Paired-pulse ratio (PPR) prior to NMDA are plotted against PPR after acute (b) or long-term application (e) of NMDA in $NR1^{fl/fl}$ mice (yellow open circles) and $SNS\text{-}NR1^{-/-}$ mice (blue open circles). (c, f) C-eEPSCs recorded in $NR1^{fl/fl}$ mice showed clear change of PPR following acute (c) or long-term application (f) of NMDA, which is significantly different from $SNS\text{-}NR1^{-/-}$ mice. n = 6-9, ***P < 0.001 by Mann Whitney U test in (c). n = 8-12, ***P < 0.001 by unpaired t test with Welch's correction in (f). (g, j) Traces of typical recordings showing paired-pulse facilitation (PPF) or paired-pulse depression (PPD) of C-eEPSCs prior to (grey traces) and following acute (g) or long-term exposure (j) to NMDA (pink traces) in CFA-inflamed state. (h, k) Paired-pulse ratio (PPR) prior to NMDA are plotted against PPR after acute (h) or long-term application (k) of NMDA in inflamed $NR1^{fl/fl}$ mice (green open circles) and inflamed $SNS\text{-}NR1^{-/-}$ mice (pink open circles). (i, l) C-eEPSCs recorded in inflamed $NR1^{fl/fl}$ mice showed clear change of PPR following acute (i) or long-term application (l) of NMDA, which is significantly different from inflamed $SNS\text{-}NR1^{-/-}$ mice. n = 6-8 for (i) and 8-10 for (l), **P < 0.01 by Mann Whitney U test in (c) and (l). Data are represented as mean ± S.E.M. See Supplemental Table 2 for detailed statistical information.

Supplementary figure 6

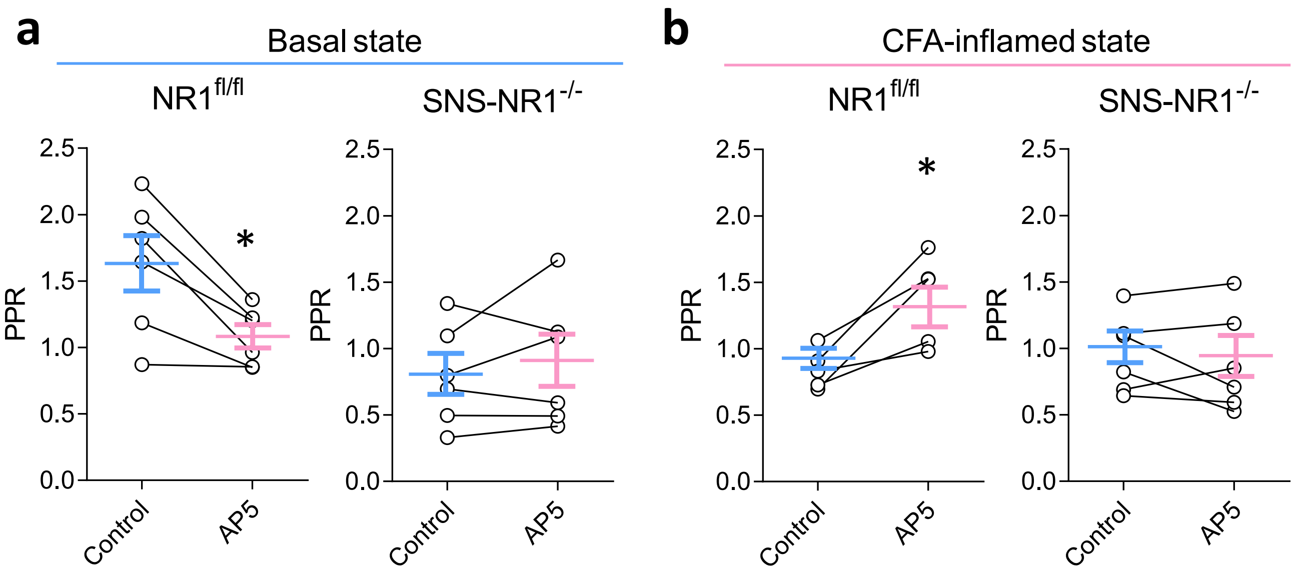

Supplementary Figure 6: The absolute changes of PPR following blockade of endogenous PreNMDARs in different states from $NR1^{fl/fl}$ mice and SNS-$NR1^{-/-}$ mice. The absolute changes of PPR after endogenous blockade of PreNMDARs with AP5 application in the basal state (a) and CFA-inflamed state (b) from $NR1^{fl/fl}$ mice and SNS-$NR1^{-/-}$ mice (n = 6). *$P < 0.05$ by paired *t* test. Data are represented as mean ± S.E.M. See Supplemental Table 2 for detailed statistical information.

Supplementary figure 7

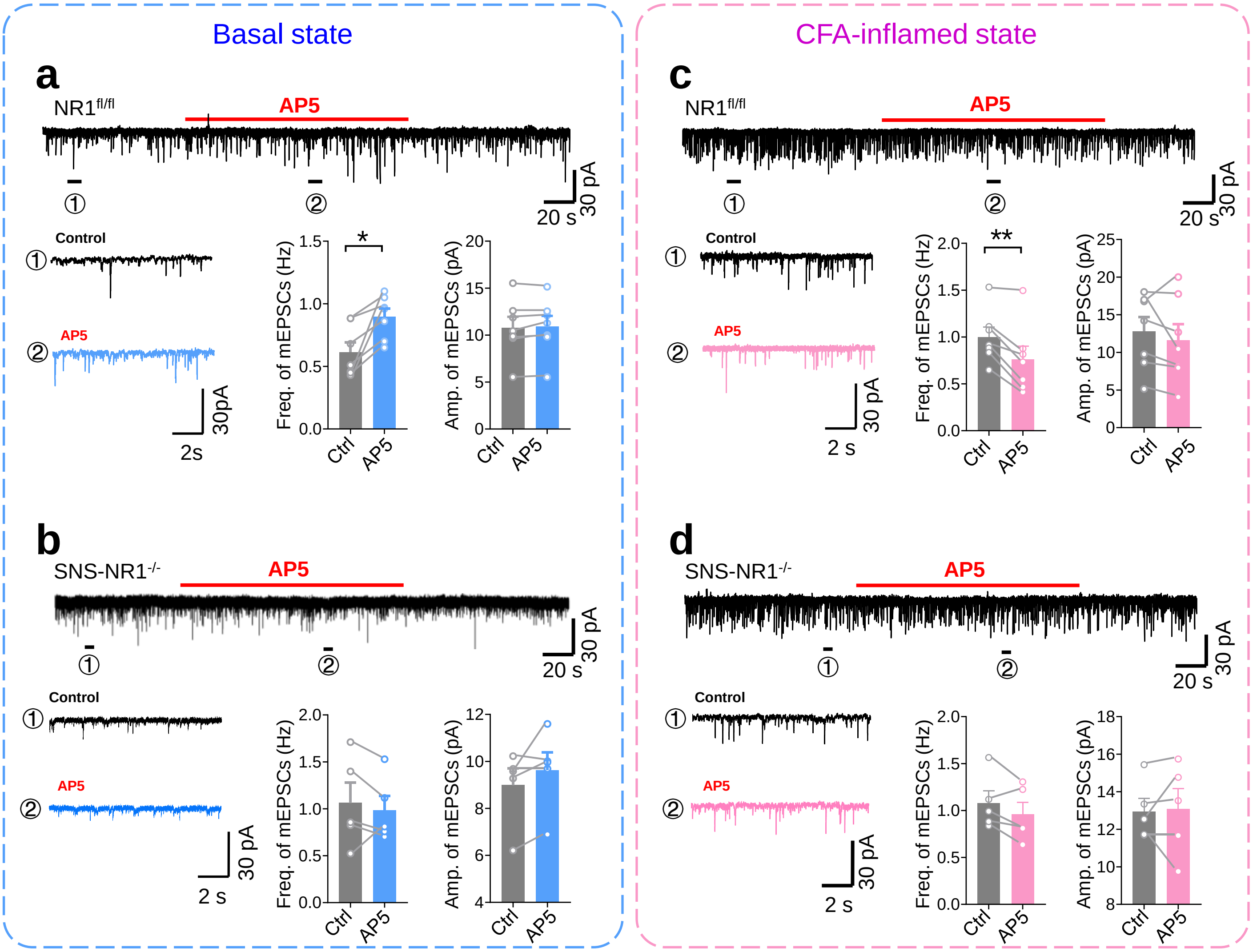

Supplementary Figure 7: The effect of blockade of endogenous PreNMDARs with AP5 on mEPSCs in the basal state and CFA-inflamed state in $NR1^{fl/fl}$ mice and SNS-$NR1^{-/-}$ mice. (a, b) Representative examples and quantitative summary showing bath application of AP5 (50 μM) caused a prominent increase in the frequency, but not amplitude of mEPSCs recorded in spinal neurons in $NR1^{fl/fl}$ mice in the basal state (a), whereas this did not come about in SNS-$NR1^{-/-}$ mice (b) (n = 7). *P < 0.05 by paired t test. (c, d) Following CFA-induced paw inflammation, AP5 application induced a marked reduction of mEPSCs frequency in $NR1^{fl/fl}$ mice (c), which was not observed in SNS-$NR1^{-/-}$ mice (d) (n= 5). **P < 0.01 by paired t test. mEPSCs were recorded and analyzed in the presence of A-803467 (0.5 μM), an Nav1.8 selective blocker besides TTX, gabazine and strychnine. Data are represented as mean ± S.E.M. See Supplemental Table 2 for detailed statistical information. Ctrl, control.

Supplementary figure 8

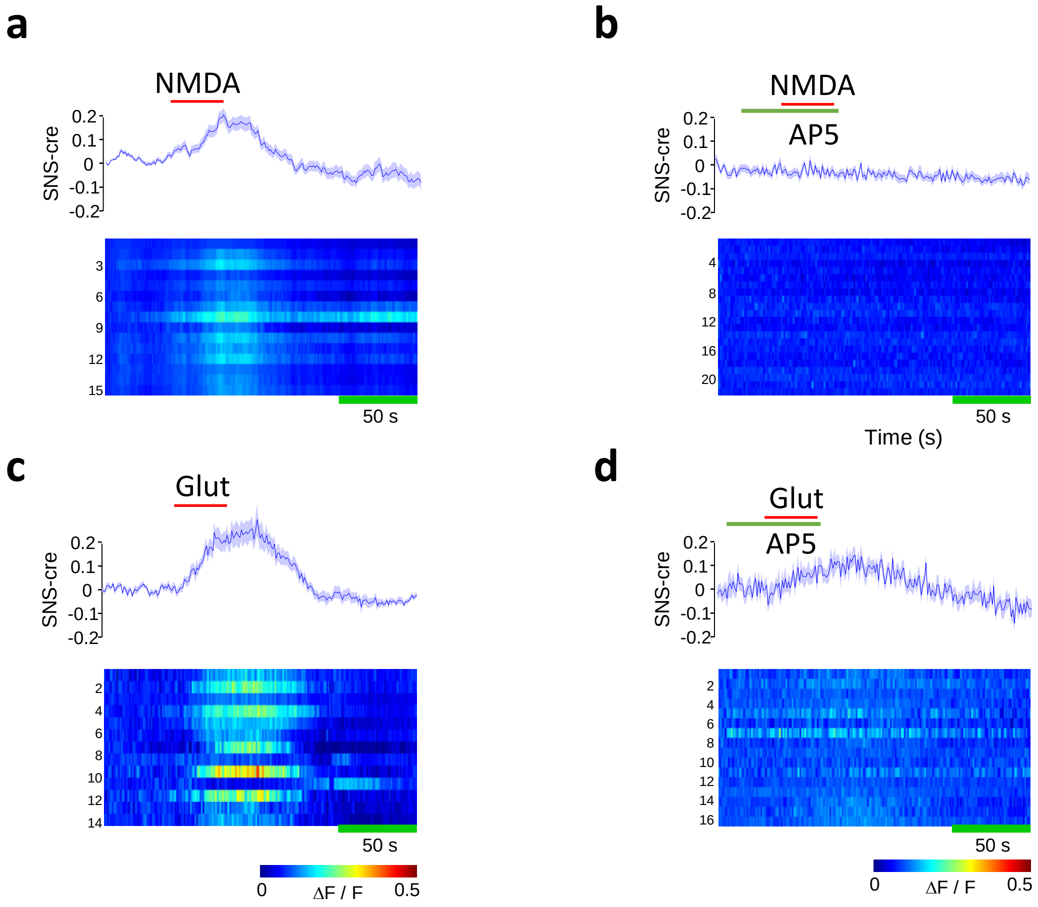

Supplementary Figure 8: Effects of PreNMDARs in the calcium transients evoked by NMDA and glutamate application in spinal slices from SNS-Cre mice. (a, b) Typical examples of traces of calcium transients as shown in heat maps and quantitative summary of peak GCaMP6s signals evoked by NMDA (50 μM) in the absence (a) and presence (b) of AP5 in spinal slices from SNS-Cre mice. (c, d) Typical examples of traces of calcium transients as shown in heat maps and quantitative summary of peak GCaMP6s signals evoked by glutamate (Glut, 1 mM) in the absence (c) and presence (d) of AP5 in spinal slices from SNS-Cre mice. Each row in y-axis represents a GCaMP6s-labelled presynaptic terminal puncta. A total of 15-22 puncta are illustrated for each agonist. Data are represented as mean ± S.E.M. Glu, glutamate.

Supplementary figure 9

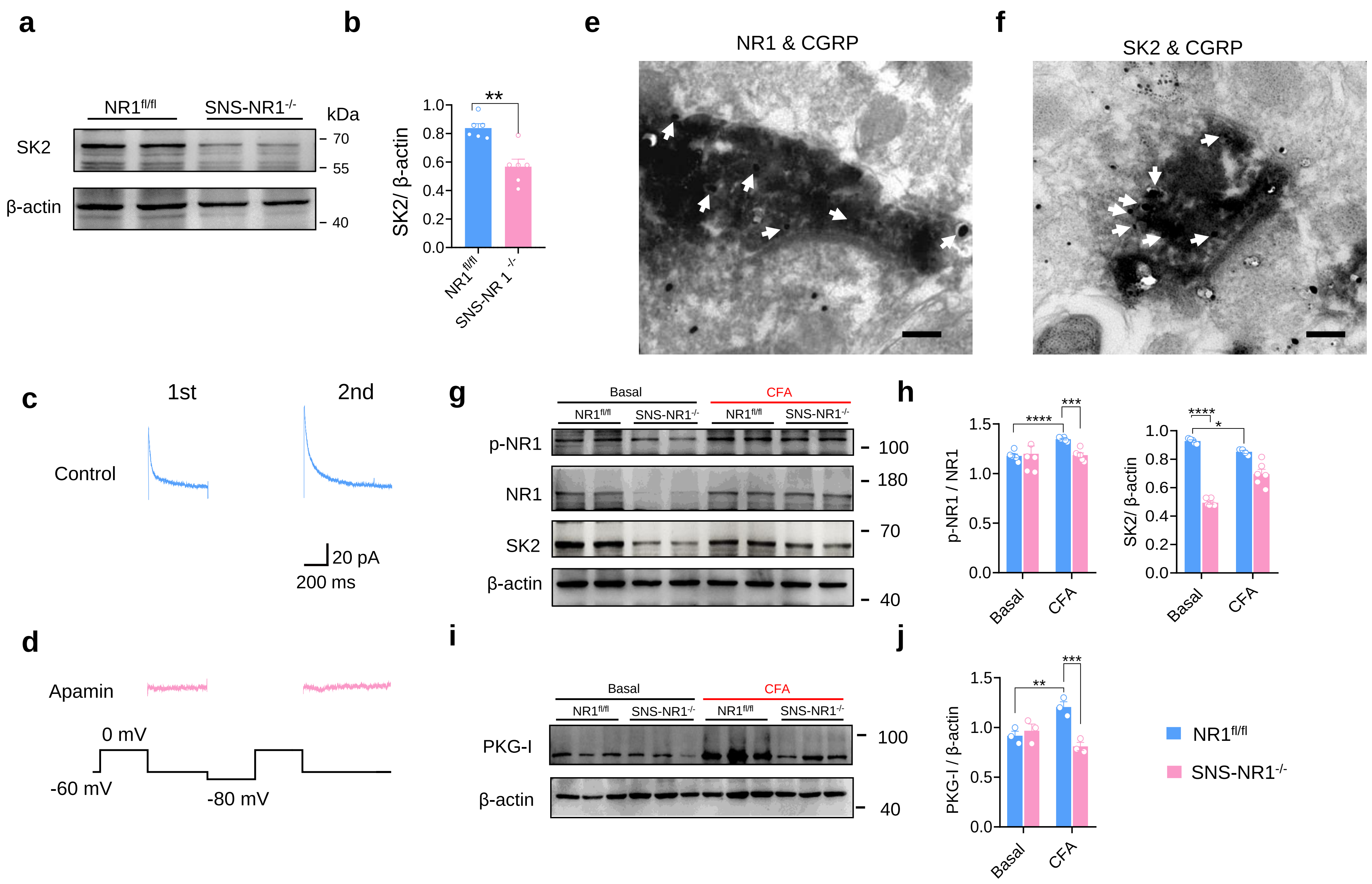

Supplementary Figure 9: Changes of SK2 and PreNMDARs in lumbar dorsal roots following peripheral inflammation. (a, b) Expression of SK2 subunit were downregulated in L3/L4 dorsal roots derived from nociceptor-specific $NR1^{-/-}$ mice as compared with $NR1^{fl/fl}$ mice in the basal state (n = 6). $^{**}P < 0.01$ by unpaired *t* test. (c, d) Identification of apamin-sensitive AHP currents. Sample traces showing AHP currents before (c) and after bath application of apamin (d,100 nM). Stimulating protocols for recording AHP currents are shown below. (e, f) Photomicrographs showing double labelling of the PreNMDARs NR1 subunits (panel e: arrowheads) and SK2 channels (panel f: arrowheads) in nociceptive terminals that are CGRP-positive in the superficial dorsal horn. Scale bar = 0.2 μm. (g, h) A typical example (g) and quantitative summary (h) of levels of phosphorylated NR1 and SK2 in L3/L4 dorsal roots of SNS-$NR1^{-/-}$ mice and $NR1^{fl/fl}$ littermates in the naive state or following CFA injection in the hindpaws (n = 4-6). $^{*}P < 0.05$, $^{***}P < 0.001$, $^{****}P < 0.0001$ by Brown-Forsythe ANOVA test and Uncorrected Fisher's LSD one-way ANOVA. (i, j) A typical example (i) and quantitative summary (j) of levels of PKG-I in L3/L4 dorsal roots of SNS-$NR1^{-/-}$ mice and $NR1^{fl/fl}$ littermates in the naive state or following CFA injection in the hindpaws (n = 3). $^{**}P < 0.01$, $^{***}P < 0.001$ by Uncorrected Fisher's LSD one-way ANOVA. Data are represented as mean ± S.E.M. See Supplemental Table 2 for detailed statistical information.

Supplementary figure 10

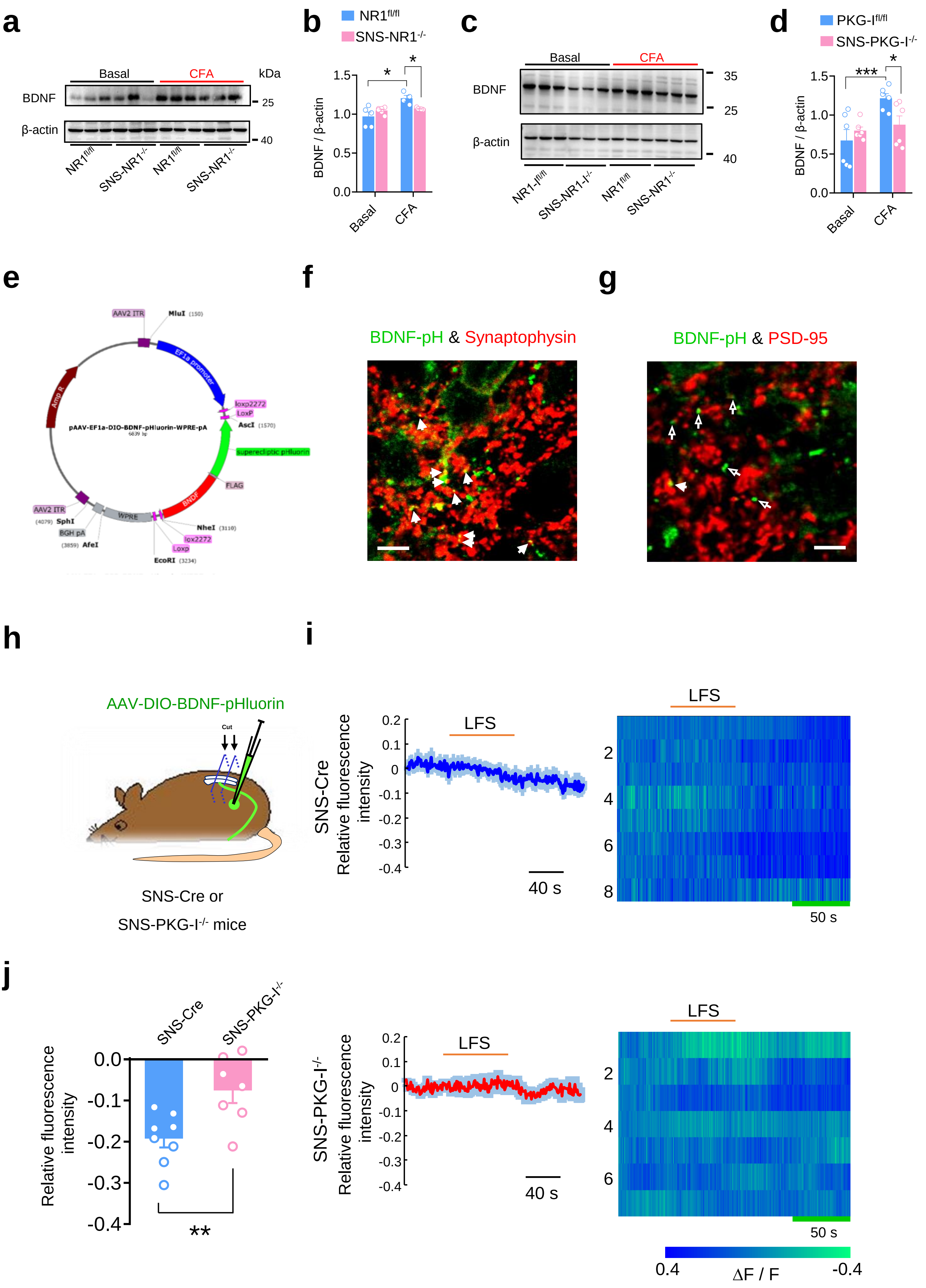

Supplementary Figure 10: Production and secretion of BDNF in lumbar dorsal roots following peripheral inflammation is dependent on PreNMDARs-PKG-I signalling in spinal terminals of nociceptors. (a, b) A typical example (a) and quantitative summary (b) showing levels of BDNF in L3/L4 dorsal roots of SNS-NR1$^{-/-}$ mice and NR1$^{fl/fl}$ littermates in the basal or CFA-inflamed state (n = 4-6). *P < 0.05 by Brown-Forsythe ANOVA test. (c, d) A typical example (c) and quantitative summary (d) showing levels of BDNF in L3/L4 dorsal roots of SNS-PKG-I$^{-/-}$ mice and PKG-I$^{fl/fl}$ littermates in the basal or CFA-inflamed state (n = 3). *P < 0.05, ***P < 0.001 by Uncorrected Fisher's LSD one-way ANOVA. (e) Schematic diagram showing the construction of AAV2/8-EF1a-DIO-BDNF-pHluorin. (f) Double immunofluorescence staining images showing colocalization of BDNF-pHluorin (BDNF-pH) puncta with the presynaptic marker synaptophysin (arrowheads), suggesting the presence of BDNF-pHluorin in presynaptic terminals. (g) Double immunofluorescence staining images showing juxtaposition of many BDNF-pHluorin (BDNF-pH) puncta with the postsynaptic marker PSD-95 (empty arrowheads) but very few colocalized BDNF-pH and PSD-95 puncta (arrowheads). Scale bar = 5 μm. (h-j) Presynaptic BDNF-pHluorin secretion depends on PKG-I localized in spinal terminals of nociceptors. (h) Scheme illustrating the experimental approach for imaging activity-induced changes in BDNF-pHluorin fluorescence from presynaptic terminals of nociceptors, using dorsal root-attached spinal cord slices. AAV2/8 expressing Cre-dependent BDNF-pHluorin was injected into lumbar DRGs of SNS-Cre or SNS-PKG-I$^{-/-}$ mice. (i, j) Sample traces (i) and quantitative summary (j) of BDNF-pHluorin fluorescence changes evoked by LFS in SNS-Cre mice (upper panels: blue) and SNS-PKG-I$^{-/-}$ mice (lower panels: red). Shown in right panels are changes of BDNF-pHluorin fluorescence

evoked by LFS in colour maps. Each row in y-axis represents a GCaMP6s-labelled presynaptic terminal puncta. A total of 7-8 puncta are illustrated for each agonist. **P < 0.01 by unpaired t test. Data are represented as mean ± S.E.M. See Supplemental Table 2 for detailed statistical information.

Supplementary figure 11

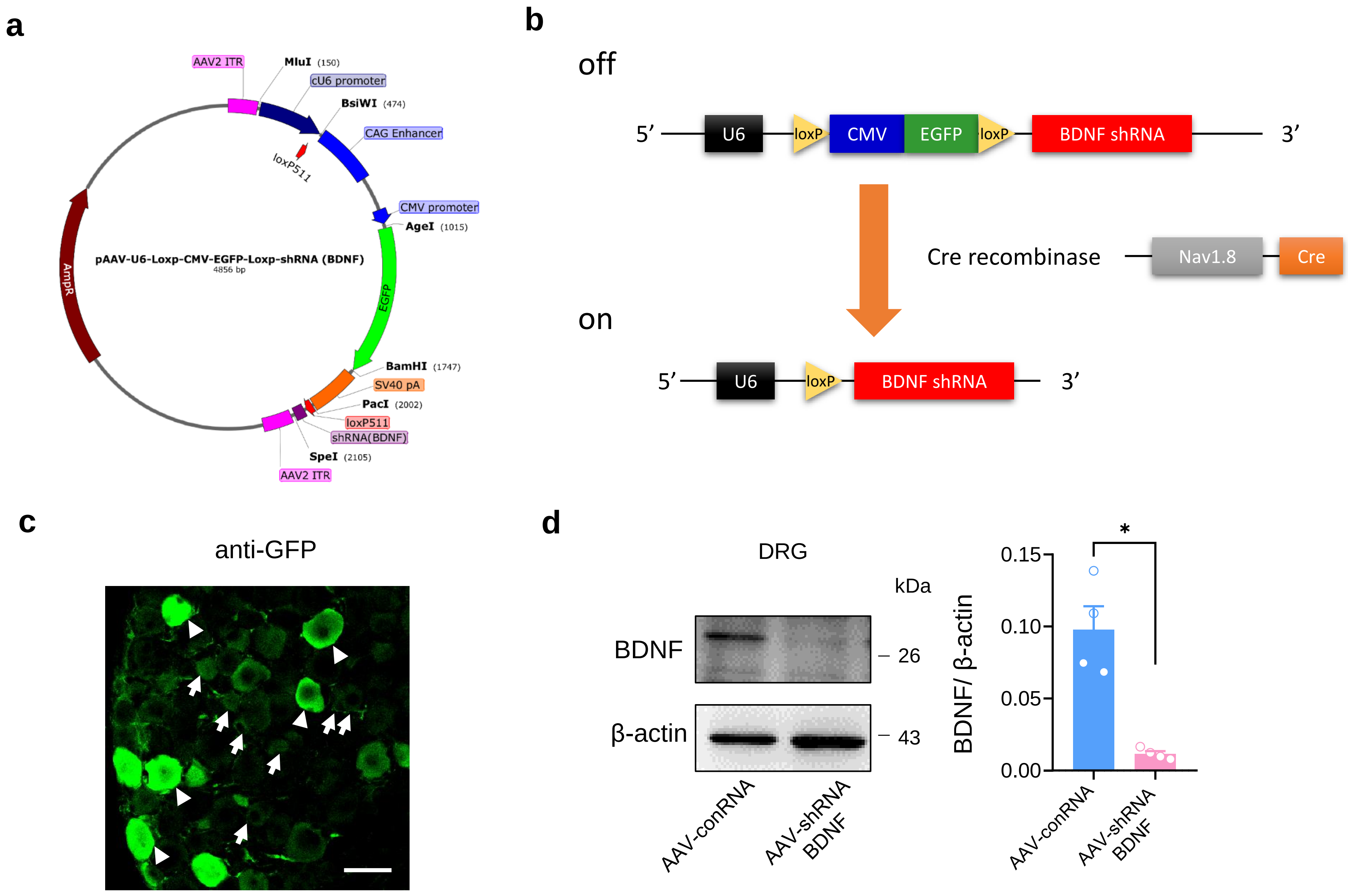

Supplementary Figure 11: Establishment and verification of BDNF knockdown specifically in nociceptive DRG neurons. (a) Schematic diagram showing the construction of Cre-dependent AAV2/8 expressing shRNA BDNF (AAV2/8-U6-Loxp-CMV-EGFP-Loxp-shRNA BDNF). (b) Injection of Loxp-BDNF shRNA-expressing AAV2/8 into SNS-Cre expressing mice enable BDNF shRNA transcription in a nociceptor-specific manner. (c) Immunostaining images showing nociceptive-specific knockdown of BDNF via injection of rAAV2/8-U6-Loxp-CMV-EGFP-Loxp-shRNA (BDNF) into L3/L4 DRGs of SNS-Cre mice. Note that GFP immunoreactivity is mainly absent in small- to medium-diameter DRG neurons, but remains intact in large-diameter DRG neurons. (d) Western blot analysis with anti-BDNF antibody confirmed that AAV2/8-shRNA BDNF expressing mice showed a dramatic loss of BDNF as compared to AAV2/8-conRNA expressing mice. Shown are typical example blots in left panels and quantitative summary in right panels (n = 4). $^{*}P < 0.05$ by unpaired *t* test with Welch's correction. Scale bar = 100 μm in (c). Data are represented as mean ± S.E.M. See Supplemental Table 2 for detailed statistical information.

Supplementary figure 12

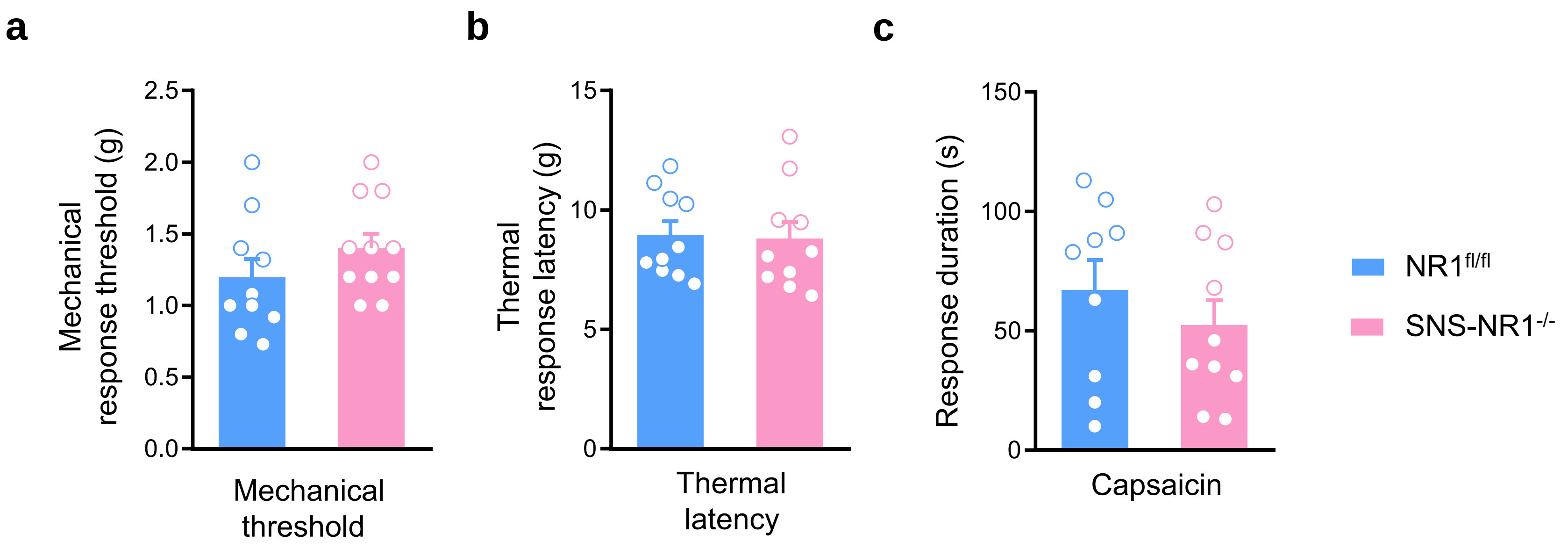

Supplementary Figure 12: Comparison of basal nociception and capsaicin-induced spontaneous nociception in $NR1^{fl/fl}$ mice and $SNS\text{-}NR1^{-/-}$ mice. (a, b) $SNS\text{-}NR1^{-/-}$ mice (n = 10) show comparable mechanical paw withdrawal threshold to punctuate pressure (a) and paw withdrawal latency to radiant heat (b) in comparison with $NR1^{fl/fl}$ mice (n = 10-11). (c) $SNS\text{-}NR1^{-/-}$ mice show no significant difference in the duration of acute nocifensive responses to intraplantar paw injection of capsaicin in comparison with $NR1^{fl/fl}$ mice (n = 10). Data are represented as mean ± S.E.M. See Supplemental Table 2 for detailed statistical information.

Supplementary figure 13

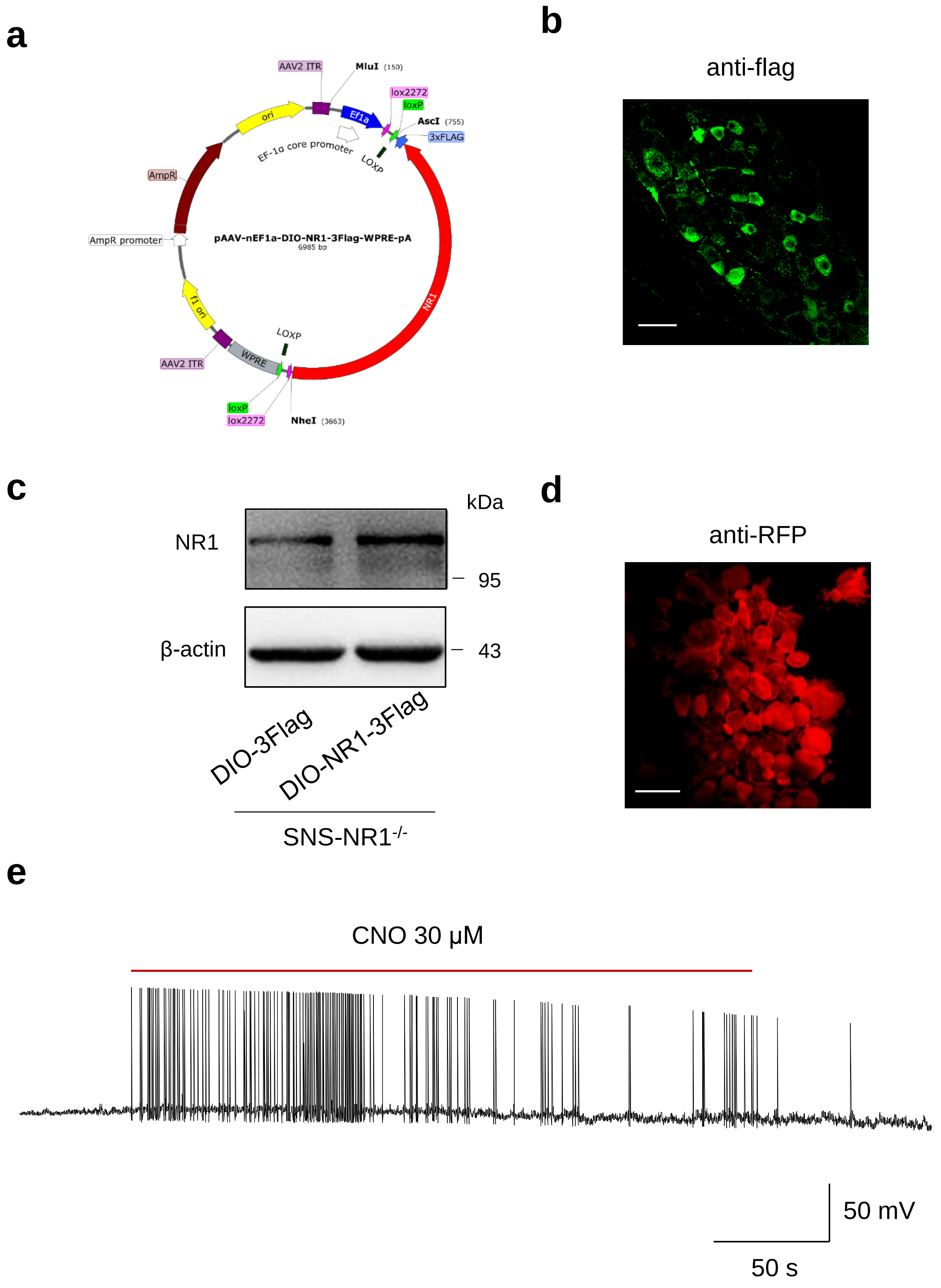

Supplementary Figure 13: Establishment and verification of NR1 expression specifically in nociceptive DRG neurons as well as activation of nociceptive DRG neurons via chemogenetic approaches. (a) Schematic diagram showing the construction of Cre-dependent AAV2/8 overexpressing NR1. (b, c) Immunostaining images (B) and Western blot analysis (c) showing NR1 is overexpressed via injection of AAV2/8-EF1α-DIO-NR1-3Flag into L3/L4 DRGs in SNS-NR1$^{-/-}$ mice. Scale bar = 100 μm. (d) Photomicrographs showing expression of DIO-hM3Dq-mcherry in the L3/L4 DRGs upon delivery via adeno-associated virions (AAV). (e) Whole-cell patch clamp recording from mcherry-positive DRG neurons revealed that bath application of CNO induces spike discharges. Scale bar = 50 μm in (b) and (d).

Supplementary Table 1

KEY RESOURCES TABLE

REAGENT or RESOURCE	SOURCE	IDENTIFIER	Dilution
Antibodies			
Biotinylated griffonia simplicifolia lectin I, Isolectin B4	Vector laboratories	B-1205	IHC 1:200
isolectin GS-IB4 from Griffonia simplicifolia, Alexa Fluor® 488 conjugate	Molecular Probes, Invitrogen	I21411	IHC 1:300
Neurobiotin	Vector laboratories	SP-1120	1%
Alexa Fluor® 488-conjugated	Invitrogen	S11223	IHC 1:500
Rabbit anti-CGRP	Calbiochem	PC250L	IHC 1:500
Goat anti-CGRP	Abcam	ab 36001	IHC 1:500
Mouse anti-NF200	Sigma-Aldrich	N2912	IHC 1:200
Rabbit anti-PSD-95	Abcam	ab18258	IHC 1:500
Rabbit anti-synaptophysin	Abcam	ab32127	IHC 1:500
Goat anti-GFP	Rockland	RK-600-102-[illegible]	IHC 1:500
Rabbit anti-RFP	Abcam	ab62341	IHC 1:500
Mouse anti-Flag	Abbkine	1B10	IHC 1:800
Rabbit anti-NR1	Abcam	ab109182	WB 1:1000
Goat anti-NR1	Santa Cruz Biotechnology	sc-1467	WB 1:500/IHC: 1:100
Rabbit anti-phosphoNR1	Santa Cruz Biotechnology	sc-31669	WB 1:500
Rabbit anti-SK2	Alomone Labs	APC-028	IHC 1:500/WB:1:200

Rabbit anti-BDNF	Novus	NB100-98682	WB 1:500
Rabbit anti-PKG-I	Gift from Prof. Robert Feil		WB 1:4000
Rabbit anti-β-actin	Sigma-Aldrich	A1978	WB 1:5000
Anti-rabbit IgG,HRP-linked Antibody	Cell Signaling Technology	7074	WB 1:5000
Donkey anti-Goat IgG H&L(Alexa Fluor®488)	Abcam	ab150129	IHC 1:300
Goat anti-Rabbit IgG (H+L) Highly Cross-Adsorbed	Invitrogen	A11034	IHC 1:500
Donkey anti-Mouse IgG (H+L) Highly Cross-	Invitrogen	A21202	IHC 1:500
Donkey anti-Mouse IgG (H+L) Highly Cross-	Invitrogen	A21203	IHC 1:500
Donkey anti-Rabbit IgG H&L (Alexa Fluor® 594)	Abcam	ab150076	IHC 1:300
Donkey anti-Goat IgG (H+L) Cross-	Invitrogen	A11058	IHC 1:500
Chemicals, Peptides and Recombinant Proteins			
NMDA	Sigma-Aldrich	M3262	
AP5	Sigma-Aldrich	A8054	
Gabazine	Sigma-Aldrich	S106	
Strychnine	Sigma-Aldrich	S8753	
Apamin	Sigma-Aldrich	A1289	
MK801	Sigma-Aldrich	M107	
QX314	Sigma-Aldrich	L5783	
BAPTA	Sigma-Aldrich	A1076	

CGP78608	TOCRIS	No.1493
UBP310	TOCRIS	No.3621
A-803467	Sigma-Aldrich	A3109
Tetrodotoxin (TTX)	TOCRIS	43F
collagenase	Sigma-Aldrich	C0130
trypsin	Sigma-Aldrich	T2600000
ketamine	Sigma-Aldrich	K-002
1.4 nm gold particles	Nanoprobes	06D232
TrkB-IgG	Alomone lab	PRC-001
Clozapine-N-Oxide	Sigma-Aldrich	C0832
Formalin	Sigma-Aldrich	HT5012
Freund’s Adjuvant, Complete	Sigma-Aldrich	F5881
KCl	Sigma-Aldrich	746436
Capsaicin	Tocris	0462
Glutamate	Sigma-Aldrich	G1626
BDNF	PeproTech	450-10
8-pCPT-cGMP	Sigma-Aldrich	C5438
DiI	Molecular [illegible]	D3911
^{10}panx1	Sigma-Aldrich	SML2152
Kainic acid	Sigma-Aldrich	420318
Virus Strains		
rAAV2/8-hSyn-DIO-hM3D(Gq)-mCherry-WPRE-pA	BrainVTA	PT-0019
rAAV2/8-CAG-DIO-GCaMP6s-WPRE-pA	BrainVTA	PT-0196

rAAV2/8-hSyn-DIO-mCherry-WPRE-pA	BrainVTA	PT-0115	
rAAV2/8-u6-Loxp-cmv-EGFP-Loxp-shRNA	BrainVTA	PT-0415	
rAAV2/8-u6-Loxp-cmv-EGFP-Loxp-shRNA control	BrainVTA	PT-0552	
rAAV2/8-EF1a-DIO-BDNF-pHluorin-WPRE-pA	BrainVTA	PT-0395	
rAAV2/8-nEF1a-DIO-NR1-3Flag-WPREs-pA	BrainVTA	PT-0298	
rAAV2/8-nEF1a--DIO-3Flag-WPREs-pA	BrainVTA	PT-0315	
rAAV2/8-Ef1a-DIO-GCaMp6s-WPRE-PA	BrainVTA	PT-0071	
rAAV2/8-CMV-DIO-shBDNF-EGFP-WPRE-pA	BrainVTA	PT-3833	
Experimental models: Organisms/Stains			
Mouse: SNS-Cre	Dr. Rohini Kuner	N/A	
Mouse: SNS-NR1$^{-/-}$	Dr. Rohini Kuner	N/A	
Mouse: SNS-PKG-I$^{-/-}$	Dr. Rohini Kuner	N/A	
Software			
Clampex 9.2	Axon	N/A	
Clampfit 10.6	Axon	N/A	

Olympus Fluoview version 3.1	Olympus	N/A
Other		
Microliter syringe pump	KD Scientific	LEGATO 130
Fine bore polythene tubing	Smith Medical	0.28mm ID, 0.61mm OD
FV1000 laser-scanning confocal microscope	Olympus	N/A
FV3000 laser-scanning confocal microscope	Olympus	N/A
Model 400 heated base	IITC Life Science	Model 400 Model 390G
von Frey Hairs	Bioseb	N/A
MultiClamp 700B	Axon Instrument	N/A
Primers		
Cre primer 1 Cre A	Sangon	GAAAGCAGCCATGTCCAATTTACTGACCGTAC
Cre primer 2 Cre E	Sangon	GCGCGCCTGAAGATATAGAAGA
PKG-I primer 1 RF 53	Sangon	CCTGGCTGTGATTTCACTCCA
PKG-I primer 2 RF118	Sangon	AAATTATAACTTGTCAAATTCTTG
PKG-I primer 3 RF125	Sangon	GTCAAGTGACCACTATG
NR1 primer 1 NR1Ex18 do 1	Sangon	CTGGGACTCAGCTGTGCTGG
NR1 primer 2 NR1In18 up 1	Sangon	AGGGAGGCAACACTGTGGAC
shRNA BDNF	BrainVTA	GCCCGACTACGCCGGTGCACC

NR1(NMDA1):<br>NM_008169.3<br>mouse	BrainVTA	GCTCTCCCTATGACGGGAACACAGCTGCAGCTGGCCCTCCTCCCTCT<br>CAATAGCGCGTCGCGGCAGCACTGTGTCTTTTTGGTTTTGCAAAGCGC<br>CGCGTCCACCCCCGGTGCTCGTGTCTTTGGAGGACCTACGTCTCTTGA<br>AGCTGGAGGCCAGGGTGGAGGTGATAGCCCTAAATGTGGCTTTCTTT<br>TTAGGGTCGGGCTCTGCTCTACCACTCTTTCTATCCTGCAGGTTCTTCC<br>TCCACACGTTCACGGCTGCAAAAGCCAGCTGCATCTGCTTCCTACGG<br>GCATCCTTGTGTCGCTTGTAGGCGATCTCGATGAAAATGAGGAAGAT<br>CCCAGCTACGATGCCTCCAGCCACCAGCATGAAGACCCCTGCCATGT<br>TCTCAAAAGTGAGGGTGGCAGGGGCATTGCTGCGGGAGTCACATTCT<br>TGATACCGAACCCATGTCTTATCCAGGTCTTCCATGAAGCCATTCTCA<br>TGGGACTTGAGTATGGACAGGGACACATTTTGCTTCCAGGGGCTGTC<br>CTTGCGCATGCCGATGCCAAAGCCGGAGCGGAAGAACAGCTCACCC<br>GTGGTCACCAGATCGCACTTCTGTGAAGCCTCAAACTCCAGCACAGC<br>TGAGTCCCAGATGAAGGCATGGAGCTTGTTGTCCCGCACAGCCTGGA<br>TGGCCTCAGCTGCACTCTCATAATTGTGCTTCTCCATGTGCCGGTACA<br>TGGTGCTCAACTCCACCTGCCTCCGGAAGTAGATATCCACAGAGCTCT<br>GTTTTACAGTTGCATAGATGAACTTGTCTGAGGGGTTTCTGAGCCTGG<br>GGTCATTGATGCCTGTGATGCGCTCCTCAGGCCTATCCAGCACCAGG<br>AAGGCTGCCAGGTTGGCAGTGTAGGAAGCCACGATGATCATGGCAA<br>AACCAGCCCACACCATGCCTAGGATACGAGCAGAGAAACTCCGGGG<br>GGCACCTTCCCCAATGCCAGAGTTGAGCAGGACGCCCCAGGAAAAC<br>CACATGGCAGAGGACAGGGTCAGTGCATCCTCCTCCTCCTCCTCGCT<br>GTTCACCTTAAATCGGCCAAAGGGACTGAAGCGGTCCAGCAGGTACA<br>GCATCACGGCCACCACATGAACTGACAGCCCCACCAGCAGCCACAG<br>TGTGCTCTGAAAGGGCTGCATGAATGAGTCCAGTGTGCTCCGAGGGA<br>TCTCCTTCTTGACCAGAATGGTCAGGCCCTGGTACTTGAAGGGCTTGG<br>AGAACTCTATGTACTGCGCACGCTCATTGTTAATGGTCAGTGGAGCCA<br>CGATCATGTCTGCTTGACCACTGAGCAGCTCTCCCATCATTCCGTTCC<br>ACTCCTTTTTGTTGCTGTTGTTTACCCGCTCCTGTGTGCCAAACTTGCC<br>ATCTGCCACAAGGTGCACCTCGTAGGTAAAATTCATGGTCCGTGCCA<br>GCTTGATGAGCAGGTCAACGCAGAAGCCATAACAGCACTGGGGCAC<br>TGTGTGACGTGGGCTTCCTGGGGATGTGTCATTAGGCCCCGTACAGA<br>TCACCTTCTTGACAGGGTCACCATTGACTGTGAACTCCTCTTTGCATGT<br>GCCATCACTCATTGTGGGCTTGACATACACGAAGGGTTCTTGGTGGAT<br>TGTCACTATCTTTAGTCTGGTGGACATCTGGTATCCTCGAGGCTTCTCT<br>GTCTCTCCTCCTGGCCAGATGATCTTCCTGTCATTTGGGATGACATGG<br>GTACCATTGTAGATGCCCACTTGCACCAGCTTGCGGTTCTGCAGGTTC<br>ATGATACTATAGTTGGCAAACTTCCGGTCCCCATCCTCATTGAATTCC<br>ACACGGCCAGTCACTCCATCTGCATACTTGGAAGACATCAGCACCCT<br>CTTGAACAGTGGTCCTGTCTTCCAGATGTTGGTGTTGCCCACGCAACC<br>CCGCGGTGGATCAGTGATGTTCTCCTTTTCTAGGAGCTCGTGGACTGC<br>CTGTGCCACCACGCCCACAGCGTCACTGATGTGGGCCGACTCGTTCT<br>TGCCGTTGATTAGCTGAAGTCCGATGATGCCGTCAGGAGCGTAGCGC

AGGGCATTCCCAGAGATCTCGCGCTCCCCGACGAGCCACACGTACCC
AGAGCCAGTCATGTTCAGCATCGCGGCTGCGCGGTATACGGTGGCAG
CGTCGTCCTCGCTTGCAGAAAGGATGATGACCCGGGCTTCCAGGTCC
CGGGCTTCCATCAGCAGAGCCGTCACATTCTTGGTTCCTGGGTCAAAC
TGCAGCACCTTCTCTGCCTTGGACTCACGCTCCTCCAGCAACGTCTCC
AGGCGCTTCTGCGCTGCCCGGCCCTCGTGGTCATCGCTGACCAGCAG
GATGATATGGTTCCAGTTGTAGACGCGCATCATCTCAAACCAGACGCT
GGACTGGTGGGAGTAGGGTGGTACGGTGCGAAGGAAGCTCAGGTGG
ATGCTCTTGTCAGAGTAGATGGACATTCGGGTAGTCAGCCCCAGGAC
GGGGATTCTGTAGAAGCCAGCTGTGTAGGAGACAGGGGTGGGAGTG
AAGTGGTCGTTGGGAGTAGGCGGGTGACTAACTAGGATAGCGTAGAC
CTGGCTAGAGATGAGGTCCTCACACACTGACAGGGCCATCTGTATGG
CGTTGGGCTTGTGGGTGACAGAAGTGGCGTTGAGCTGTATCTTCCAA
GAGCCGTGTCGCTTATTGGCCTGGTTTACTGCCTCGCGGAACATCTGC
TCGTGCTTGCGCGTGCTCAGCACCGCGCCGATGTTGACAATCTTGGG
GTCGCAGGCAGCGCGGGCGAAGGAGCAGGAGAAAAGCAGGGCGAA
TGTCAGCAGGTGCATGGTGCTCAT

BDNF-pH	BrainVTA	ATGACCATCCTTTTCCTTACTATGGTTATTTCATACTTTGGTTGCATGAA<br>GGCGGCCGCCGGAGGCTACCCCTACGACGTGCCCGACTACGCCGGT<br>GCACCCATGAAAGAAGCAAACATCCGAGGACAAGGTGGCTTGGCCT<br>ACCCAGGTGTGCGGACCCATGGGACTCTGGAGAGCGTGAATGGGCC<br>CAAGGCAGGTTCAAGAGGCTTGACATCATTGGCTGACACTTTCGAAC<br>ACGTGATAGAAGAGCTGTTGGATGAGGACCAGAAAGTTCGGCCCAAT<br>GAAGAAAACAATAAGGACGCAGACTTGTACACGTCCAGGGTGATGCT<br>CAGTAGTCAAGTGCCTTTGGAGCCTCCTCTTCTCTTTCTGCTGGAGGA<br>ATACAAAAATTACCTAGATGCTGCAAACATGTCCATGAGGGTCCGGC<br>GCCACTCTGACCCTGCCCGCCGAGGGGAGCTGAGCGTGTGTGACAG<br>TATTAGTGAGTGGGTAACGGCGGCAGACAAAAAGACTGCAGTGGAC<br>ATGTCGGGCGGGACGGTCACAGTCCTTGAAAAGGTCCCTGTATCAAA<br>AGGCCAACTGAAGCAATACTTCTACGAGACCAAGTGCAATCCCATGG<br>GTTACACAAAAGAAGGCTGCAGGGGCATAGACAAAAGGCATTGGAA<br>CTCCCAGTGCCGAACTACCCAGTCGTACGTGCGGGCCCTTACCATGG<br>ATAGCAAAAAGAGAATTGGCTGGCGATTCATAAGGATAGACACTTCT<br>TGTGTATGTACATTGACCATTAAAAGGGGAAGAGGATCCGACTACAA<br>GGATGACGATGACAAGAGTAAAGGAGAAGAACTTTTCACTGGAGTTG<br>TCCCAATTCTTGTTGAATTAGATGGTGATGTTAATGGGCACAAATTTTC<br>TGTCAGTGGAGAGGGTGAAGGTGATGCAACATACGGAAAACTTACCC<br>TTAAATTTATTTGCACTACTGGAAAACTACCTGTTCCTTGGCCAACACT<br>TGTCACTACTTTAACTTATGGTGTTCAATGCTTTTCAAGATACCCAGAT<br>CATATGAAACGGCATGACTTTTTCAAGAGTGCCATGCCCGAAGGTTAT<br>GTACAGGAAAGAACTATATTTTTCAAAGATGACGGGAACTACAAGAC<br>ACGTGCTGAAGTCAAGTTTGAAGGTGATACCCTTGTTAATAGAATCGA<br>GTTAAAAGGTATTGATTTTAAAGAAGATGGAAACATTCTTGGACACAA<br>ATTGGAATACAACTATAACGATCACCAGGTGTACATCATGGCAGACA<br>AACAAAAGAATGGAATCAAAGCTAACTTCAAAATTAGACACAACATT<br>GAAGATGGAGGCGTTCAACTAGCAGACCATTATCAACAAAATACTCC<br>AATTGGCGATGGGCCCGTCCTTTTACCAGACAACCATTACCTGTTTAC<br>AACTTCTACTCTTTCGAAAGATCCCAACGAAAAGAGAGACCACATGG<br>TCCTTCTTGAGTTTGTAACAGCTGCTGGGATTACACATGGCATGGATG<br>AACTATACAAATA

Supplementary Table 2

Data	Response variable	Groups	n define	Animal used	NormalityTest	Homogeneity of variance test (F (DFn, DFd))	Hypothesis test	Test value	P value	Summmary
Fig1e	Normalized C-eEPSC	NR1$^{fl/fl}$(n=12), SNS-NR1$^{-/-}$(n=9)	Neuron	6 mice (21 slices)	Not all subgroups meets p > 0.05	F=8.369,DFn=3,Dfd=38,P<0.0001	Kruskal-Wallis *H* test	Kruskal-Wallis statistic=9.889	0.0195	*
		NR1$^{fl/fl}$(Basal) vs. SNS-NR1$^{-/-}$(Basal)					Uncorrected Dunn's test		0.7766	ns
		NR1$^{fl/fl}$(Basal) vs. NR1$^{fl/fl}$(30 min after LFS)					Uncorrected Dunn's test		0.0244	*
		NR1$^{fl/fl}$(Basal) vs. SNS-NR1$^{-/-}$(30 min after LFS)					Uncorrected Dunn's test		0.2279	ns
		SNS-NR1$^{-/-}$(Basal) vs. NR1$^{fl/fl}$(30 min after LFS)					Uncorrected Dunn's test		0.1848	ns
		SNS-NR1$^{-/-}$(Basal) vs. SNS-NR1$^{-/-}$(30 min after LFS)					Uncorrected Dunn's test		0.2239	ns
		NR1$^{fl/fl}$(30 min after LFS) vs. SNS-NR1$^{-/-}$(30 min after LFS)					Uncorrected Dunn's test		0.0051	**
Fig1f	Normailized C-eEPSC at 30 min after LFS(%)	NR1$^{fl/fl}$(n=12), SNS-NR1$^{-/-}$(n=9)	Neuron	6 mice(21slices)	W=0.8717,P=0.8909,W=0.8909,P=0.2039	F=7.847,DFn=11,Dfd=8, P=0.0072	Unpaired t test with Welch's correction	t=3.533, df=14.48	0.0032	**
Fig1h	Failure rate at 30 min after LFS(%)	NR1$^{fl/fl}$(n=10), SNS-NR1$^{-/-}$(n=5)	Neuron	7 mice(10 slices for NR1$^{fl/fl}$ mice and 5 slices for SNS-NR1$^{-/-}$ mice)	W=0.9667,P=0.8591,W=0.9477,P=0.6411,W=0.8601,P=0.2285,W=0.8550,P=0.2107	F=0.7356,DFn=3,Dfd=26, P=0.5403	One-way ANOVA	F (3, 26) = 109.0	<0.0001	****
		NR1$^{fl/fl}$(Basal) vs. NR1$^{fl/fl}$(30 min after LFS)					Uncorrected Fisher's LSD		<0.0001	****
		NR1$^{fl/fl}$(Basal) vs. SNS-NR1$^{-/-}$(Basal)					Uncorrected Fisher's LSD		0.8814	ns
		NR1$^{fl/fl}$(Basal) vs. SNS-NR1$^{-/-}$(30 min after LFS)					Uncorrected Fisher's LSD		0.7013	ns
		NR1$^{fl/fl}$(30 min after LFS) vs. SNS-NR1$^{-/-}$(Basal)					Uncorrected Fisher's LSD		<0.0001	****
		NR1$^{fl/fl}$(30 min after LFS) vs. SNS-NR1$^{-/-}$(30 min after LFS)					Uncorrected Fisher's LSD		<0.0001	****
		SNS-NR1$^{-/-}$(Basal) vs. SNS-NR1$^{-/-}$(30 min after LFS)					Uncorrected Fisher's LSD		0.8389	ns
Fig1k	Vertical distance to the diagonal	NR1$^{fl/fl}$(n=22), SNS-NR1$^{-/-}$(n=35)	Neuron	15 mice(22 slices for NR1$^{fl/fl}$ mice and 35 slices for SNS-NR1$^{-/-}$ mice)	Not all subgroups meets p > 0.05	F=16.95,DFn=21,Dfd=34, P<0.0001	Mann Whitney U test	Mann-Whitney U=52	<0.0001	****
		NR1$^{flox/flox}$ (n=6), SNS-NR1$^{-/-}$(n=7)	Neuron	10 mice(6 slices for NR1$^{fl/fl}$ mice and 7 slices for SNS-NR1$^{-/-}$ mice)	Not all subgroups meets p > 0.05	F=6.360,DFn=5,Dfd=26, P=0.0006	Kruskal-Wallis *H* test	Kruskal-Wallis statistic=19.51	0.0015	**

Fig2b	Normalized C-eEPSC	NR1$^{flox/flox}$Basal vs.NR1$^{flox/flox}$NMDA					Uncorrected Dunn's test		0.0008	***
		NR1$^{flox/flox}$Basal vs. NR1$^{flox/flox}$Washout					Uncorrected Dunn's test		0.0938	ns
		NR1$^{flox/flox}$Basal vs. NR1$^{-/-}$Basal					Uncorrected Dunn's test		0.8192	ns
		NR1$^{flox/flox}$Basal vs. NR1$^{-/-}$NMDA					Uncorrected Dunn's test		0.1856	ns
		NR1$^{flox/flox}$Basal vs. NR1$^{-/-}$Washout					Uncorrected Dunn's test		0.879	ns
		NR1$^{flox/flox}$NMDA vs. NR1$^{flox/flox}$Washout					Uncorrected Dunn's test		0.9135	ns
		NR1$^{flox/flox}$NMDA vs. NR1$^{-/-}$Basal					Uncorrected Dunn's test		0.0002	***
		NR1$^{flox/flox}$NMDA vs. NR1$^{-/-}$NMDA					Uncorrected Dunn's test		0.0323	*
		NR1$^{flox/flox}$NMDA vs. NR1$^{-/}$Washout					Uncorrected Dunn's test		0.004	**
		NR1$^{flox/flox}$Washout vs. NR1$^{-/-}$Basal					Uncorrected Dunn's test		0.07	ns
		NR1$^{flox/flox}$Washout vs. NR1$^{-/-}$NMDA					Uncorrected Dunn's test		0.3154	ns
		NR1$^{flox/flox}$Washout vs. NR1$^{-/-}$Washout					Uncorrected Dunn's test		0.0968	ns
		NR1$^{-/-}$Basal vs. NR1$^{-/-}$NMDA					Uncorrected Dunn's test		0.1062	ns
		NR1$^{-/-}$Basal vs. NR1$^{-/-}$ Washout					Uncorrected Dunn's test		0.9774	ns
		NR1$^{-/-}$NMDA vs. NR1$^{-/-}$ Washout					Uncorrected Dunn's test		0.2213	ns
Fig2d	Normalized C-eEPSC	NR1$^{fl/fl}$(n=9), SNS-NR1$^{-/-}$(n=10)	neuron	6 mice(9 slices for NR1$^{fl/fl}$ mice and 10 slices for SNS-NR1$^{-/-}$ mice)	W=0.9223,P=0.4116,W=0.9017,P=0.2288	F=1.355,DFn=8,Dfd=9, P=0.6577	Unpaired t test	t=4.138, df=17	0.0007	***
		NR1$^{fl/fl}$ (n=5), SNS-NR1$^{-/-}$(n=7)	neuron	9 mice(5 slices for NR1$^{fl/fl}$ mice and 7 slices for SNS-NR1$^{-/-}$ mice)	Not all subgroups meets p > 0.05	F=6.360,DFn=5,Dfd=26, P=0.0005	Kruskal-Wallis *H* test	Kruskal-Wallis statistic=15.50	0.0084	**
		NR1$^{flox/flox}$ CFA Basal vs. NR1$^{flox/flox}$ CFA NMDA					Uncorrected Dunn's test		0.0144	*

Fig2f	Normalized C-eEPSC	$NR1^{flox/flox}$ CFA Basal vs. $NR1^{flox/flox}$ CFA Washout					Uncorrected Dunn's test		0.4244	ns
		$NR1^{flox/flox}$ CFA Basal vs. $NR1^{-/-}$ CFA Basal					Uncorrected Dunn's test		0.9495	ns
		$NR1^{flox/flox}$ CFA Basal vs. $NR1^{-/-}$ CFA NMDA					Uncorrected Dunn's test		0.343	ns
		$NR1^{flox/flox}$ CFA Basal vs. $NR1^{-/-}$ CFA Washout					Uncorrected Dunn's test		0.6226	ns
		$NR1^{flox/flox}$ CFA NMDA vs. $NR1^{flox/flox}$ CFA Washout					Uncorrected Dunn's test		0.0019	**
		$NR1^{flox/flox}$ CFA NMDA vs. $NR1^{-/-}$ CFA Basal					Uncorrected Dunn's test		0.0068	**
		$NR1^{flox/flox}$ CFA NMDA vs. $NR1^{-/-}$ CFA NMDA					Uncorrected Dunn's test		0.0003	***
		$NR1^{flox/flox}$ CFA NMDA vs. $NR1^{-/-}$ CFA Washout					Uncorrected Dunn's test		0.0694	ns
		$NR1^{flox/flox}$ CFA Washout vs. $NR1^{-/-}$ CFA Basal					Uncorrected Dunn's test		0.4262	ns
		$NR1^{flox/flox}$ CFA Washout vs. $NR1^{-/-}$ CFA NMDA					Uncorrected Dunn's test		0.9754	ns
		$NR1^{flox/flox}$ CFA Washout vs. $NR1^{-/-}$ CFA Washout					Uncorrected Dunn's test		0.2206	ns
		$NR1^{-/-}$ CFA Basal vs. $NR1^{-/-}$ CFA NMDA					Uncorrected Dunn's test		0.3324	ns
		$NR1^{-/-}$ CFA Basal vs. $NR1^{-/-}$ CFA Washout					Uncorrected Dunn's test		0.5579	ns
		$NR1^{-/-}$ CFA NMDA vs. $NR1^{-/-}$ CFA Washout					Uncorrected Dunn's test		0.1578	ns
Fig2h	Normalized C-eEPSC	$NR1^{fl/fl}$(n=5), SNS-$NR1^{-/-}$(n=10)	neuron	6 mice(5 slices for $NR1^{fl/fl}$ mice and 10 slices for SNS-$NR1^{-/-}$ mice)	W=0.9438,P=0.6927,W=0.9152,P=0.3186,	F=8.967,DFn=4,Dfd=9, P=0.0067	Unpaired t test with Welch's correction	t=3.393, df=4.452	0.0232	*
		Basal(n=12),NMDA(n=12),After LFS(n=12),NMDA after LFS(n=12)	neuron	6 mice(6 slices for wildtype mice)	Not all subgroups meets p > 0.05	F=1.808,DFn=3,Dfd=44, P=0.1597	Kruskal-Wallis *H* test	Kruskal-Wallis statistic=22.22	<0.0001	****
		Basal vs. NMDA					Uncorrected Dunn's test		0.0016	**
		Basal vs. After CFS					Uncorrected Dunn's test		0.5795	ns

Fig2k	Normalized C-eEPSC	Basal vs. NMDA after CFS					Uncorrected Dunn's test		0.1489	ns
		NMDA vs. After CFS					Uncorrected Dunn's test		0.0091	**
		NMDA vs. NMDA after CFS					Uncorrected Dunn's test		<0.0001	****
		After CFS vs. NMDA after CFS					Uncorrected Dunn's test		0.0458	*
Fig3c	C-eEPSC (pA)	NR1$^{fl/fl}$(n=6), SNS-NR1$^{-/-}$(n=10), CFA-NR1$^{fl/fl}$ (n=8), CFA-SNS-NR1$^{-/-}$ (n=9)	neuron	14 mice(14 slices for NR1$^{fl/fl}$ mice and 19 slices for SNS-NR1$^{-/-}$ mice)	Not all subgroups meets p > 0.05	F=2.196,DFn=7,Dfd=58, P=0.0476	Kruskal-Wallis *H* test	Kruskal-Wallis statistic=18.41	0.0103	*
Fig3c：NR1$^{fl/fl}$ v.s. NR1$^{fl/fl}$(AP5)	C-eEPSC (pA)	NR1$^{fl/f}$(n=6), NR1$^{fl/fl}$(AP5) (n=6),	neuron	3 mice(6 slices for NR1$^{fl/fl}$ mice)	Not all subgroups meets p > 0.05	F=2.145,DFn=5,Dfd=5, P=0.4222	Wilcoxon matched-pairs signed rank test		0.0313	*
Fig3c：SNS-NR1$^{-/-}$ v.s. SNS-NR1$^{-/-}$(AP5)	C-eEPSC (pA)	SNS-NR1$^{-/-}$(n=10), SNS-NR1$^{-/-}$(AP5) (n=10),	neuron	4 mice(10 slices for SNS-NR1$^{-/-}$ mice)	W=0.8957,P=0.1962,W=0.8520,P=0.0614,	F=1.434,DFn=9,Dfd=9, P=0.5936	Paired t test	t=2.139, df=9	0.0611	ns
Fig3c：CFA-NR1$^{fl/fl}$ v.s. CFA-NR1$^{fl/fl}$(AP5)	C-eEPSC (pA)	CFA-NR1$^{fl/fl}$ (n=8), CFA-NR1$^{fl/fl}$(AP5) (n=8)	neuron	3 mice(8 slices for NR1$^{fl/fl}$ mice)	Not all subgroups meets p > 0.05	F=5.986,DFn=7,Dfd=7, P=0.0308	Wilcoxon matched-pairs signed rank test		0.0078	**
Fig3c：CFA-SNS-NR1$^{-/-}$ v.s. CFA-SNS-NR1$^{-/-}$(AP5)	C-eEPSC (pA)	CFA-SNS-NR1$^{-/-}$ (n=9), CFA-SNS-NR1$^{-/-}$ (AP5) (n=9)	neuron	4 mice(9 slices for SNS-NR1$^{-/-}$ mice)	W=0.89529,P=0.7219,W=0.9564,P=0.7599,	F=1.386,DFn=8,Dfd=8, P=0.6553	Paired t test	t=0.3230, df=8	0.755	ns
Fig3e	Vertical distance to the diagonal	NR1$^{fl/fl}$(n=6), SNS-NR1$^{-/-}$(n=10),CFA-NR1$^{fl/fl}$(n=6), CFA-SNS-NR1$^{-/-}$(n=9)	neuron	14 mice(12 slices for NR1$^{fl/fl}$ mice and 19 slices for SNS-NR1$^{-/-}$ mice)	All subgroups meets p＞0.05	F=3.568,DFn=3,Dfd=27, P=0.0270	One-way ANOVA	F (3, 27) = 5.554	0.0042	**
		NR1$^{fl/fl}$ vs. SNS-NR1$^{-/-}$					Uncorrected Fisher's LSD		0.005	**
		NR1$^{fl/fl}$ vs. CFA-NR1$^{fl/fl}$					Uncorrected Fisher's LSD		0.6337	ns
		NR1$^{fl/fl}$ vs. CFA-SNS-NR1$^{-/-}$					Uncorrected Fisher's LSD		0.0034	**
		SNS-NR1$^{-/-}$ vs. CFA-NR1$^{fl/fl}$					Uncorrected Fisher's LSD		0.0181	*
		SNS-NR1$^{-/-}$ vs. CFA-SNS-NR1$^{-/-}$					Uncorrected Fisher's LSD		0.8091	ns
		CFA-NR1$^{fl/fl}$ vs. CFA-SNS-NR1$^{-/-}$					Uncorrected Fisher's LSD		0.0124	*
Fig3f-left	Freq. of mEPSCs (Hz)	Ctrl(n=5),AP5(n=5)	neuron	3 mice(5 slices)	All subgroups meets p＞0.05	F=1.335,DFn=4,Dfd=4, P=0.7860	Paired t test	t=6.135, df=4	0.0036	**
Fig3f-right	Amp. of mEPSCs (pA)	Ctrl(n=5),AP5(n=5)	neuron	3 mice(5 slices)	All subgroups meets p＞0.05	F=1.471,DFn=4,Dfd=4, P=0.7177	Paired t test	t=1.114, df=4	0.3277	ns

Fig3h-left	Freq. of mEPSCs (Hz)	Ctrl(n=4),AP5(n=4)	neuron	3 mice(4 slices)	All subgroups meets p＞0.05	F=3.868,DFn=3,Dfd=3, P=0.2958	Paired t test	t=6.752, df=3	0.0428	*
Fig3h-right	Amp. of mEPSCs (pA)	Ctrl(n=4),AP5(n=4)	neuron	3 mice(4 slices)	All subgroups meets p＞0.05	F=1.4008,DFn=3,Dfd=3, P=0.7886	Paired t test	t=0.3576, df=3	0.7443	ns
Fig3g-left	Freq. of mEPSCs (Hz)	Ctrl(n=5),AP5(n=5)	neuron	3 mice(5 slices)	Not all subgroups meets p＞0.05	F=1.075,DFn=4,Dfd=4, P=0.9456	Paired t test	t=1.489, df=4	0.2108	ns
Fig3g-right	Amp. of mEPSCs (pA)	Ctrl(n=5),AP5(n=5)	neuron	3 mice(5 slices)	All subgroups meets p＞0.05	F=1.032,DFn=4,Dfd=4, P=0.9765	Paired t test	t=2.077, df=4	0.1064	ns
Fig3l-left	Freq. of mEPSCs (Hz)	Ctrl(n=5),AP5(n=5)	neuron	3 mice(5 slices)	All subgroups meets p＞0.05	F=1.137,DFn=4,Dfd=4, P=0.9040	Paired t test	t=0.08013, df=4	0.94	ns
Fig3l-right	Amp. of mEPSCs (pA)	Ctrl(n=5),AP5(n=5)	neuron	3 mice(5 slices)	All subgroups meets p＞0.05	F=1.927,DFn=4,Dfd=4, P=0.5408	Paired t test	t=0.5214, df=4	0.6296	ns
Fig4d	Relative Ca^{2+} response	NMDA-NR1$^{fl/fl}$(n=20), Glut-NR1$^{fl/fl}$(n=12), KCl-NR1$^{fl/fl}$(n=8), CAP-NR1$^{fl/fl}$ (n=14), NMDA-SNS-NR1$^{-/-}$ (n=8), Glut-SNS-NR1$^{-/-}$ (n=11), KCl-SNS-NR1$^{-/-}$ (n=11), CAP-SNS-NR1$^{-/-}$ (n=14)	presynaptic terminal puncta	11 mice(20 slices)	Not all subgroups meets p＞0.05	F=4.468,DFn=7,Dfd=90, P=0.0003	Kruskal-Wallis *H* test	Kruskal-Wallis statistic=43.17	<0.0001	****
		NMDA-NR1$^{fl/fl}$ vs. NMDA-SNS-NR1$^{-/-}$					Uncorrected Dunn's test		<0.0001	****
		Glut-NR1$^{fl/fl}$ vs. Glut-SNS-NR1$^{-/-}$					Uncorrected Dunn's test		0.0009	***
		KCl-NR1$^{fl/fl}$ vs. KCl-SNS-NR1$^{-/-}$					Uncorrected Dunn's test		0.0677	ns
		CAP-NR1$^{fl/fl}$ vs. CAP-SNS-NR1$^{-/-}$					Uncorrected Dunn's test		0.1021	ns
Fig4g	Relative Ca^{2+} response	SNS-Cre(Control) (n=22), SNS-Cre(AP5) (n=17), SNS-NR1$^{-/-}$(n=20)	neuron	7 mice(11 slices)	W=0.9691,P=0.6898,W=0.9398,P=0.3158,W=0.9103,P=0.0647	F=22.03,DFn=2,Dfd=56, P<0.0001	Brown-Forsythe ANOVA test	F*=22.72 (2.000, 21.93)	<0.0001	****
		SNS-Cre(Control) vs. SNS-Cre(AP5)					Unpaired t with Welch's correction		<0.0001	****
		SNS-Cre(Control) vs. SNS-NR1$^{-/-}$					Unpaired t with Welch's correction		0.0002	***
Fig5b	Relative amplitude of C-eEPSC	Basal (n=5), Apamin (n=5), Apamin+NMDA (n=5)	neuron	3 mice(5 slices)	W=0.8835,P=0.3254,W=0.9243,P=0.5581,W=0.9937,P=0.9910	F=5.918,DFn=2,Dfd=12, P=0.0163	Brown-Forsythe ANOVA test	F*=18.21 (2.000, 4.571)	0.0066	**
		Basal vs. Apamin					Unpaired t with Welch's correction		0.0014	**
		Basal vs. Apamin+NMDA					Unpaired t with Welch's correction		0.0071	**
		Apamin vs. Apamin+NMDA					Unpaired t with Welch's correction		0.0394	*

Fig5d	Amplitude of AHP current(pA)	NR1$^{fl/fl}$-1st (n=5), NR1$^{fl/fl}$-2nd (n=5), SNS-NR1$^{-/-}$-1st (n=6), SNS-NR1$^{-/-}$-2nd (n=6)	neuron	6 mice(5 slices for NR1$^{fl/fl}$ mice and 6 slices for SNS-NR1$^{-/-}$ mice)	W=0.9103,P=0.4696,W=0.9943,P=0.9923,W=0.8533,P=0.1673,W=0.8638,P=0.2025	F=3.0041,DFn=3,Dfd=18, P=0.0576	One-way ANOVA	F (3, 18) = 19.02	<0.0001	****
		NR1$^{fl/fl}$-1st vs. NR1$^{fl/fl}$-2nd					Uncorrected Fisher's LSD		0.0831	**
		NR1$^{fl/fl}$-1st vs. SNS-NR1$^{-/-}$-1st					Uncorrected Fisher's LSD		0.0148	***
		NR1$^{fl/fl}$-1st vs. SNS-NR1$^{-/-}$-2nd					Uncorrected Fisher's LSD		0.0482	*
		NR1$^{fl/fl}$-2nd vs. SNS-NR1$^{-/-}$-1st					Uncorrected Fisher's LSD		0.0033	****
		NR1$^{fl/fl}$-2nd vs. SNS-NR1$^{-/-}$-2nd					Uncorrected Fisher's LSD		0.0068	****
		SNS-NR1$^{-/-}$-1st vs. SNS-NR1$^{-/-}$-2nd					Uncorrected Fisher's LSD		0.0029	ns
Fig5f	SK2/β-actin	NR1$^{fl/fl}$(n=3), SNS-NR1$^{-/-}$(n=3)	mouse	6 mice	W=0.7881,P=0.8531,W=0.0864,P=0.2487	F=25.90,DFn=2,Dfd=2, P=0.0744	Unpaired t test	t=6.820, df=4	0.0024	**
Fig5h-left	p-NR1/β-actin	NR1$^{fl/fl}$(n=3), SNS-NR1$^{-/-}$(n=3), NR1$^{fl/fl}$+CFA(n=3), SNS-NR1$^{-/-}$+CFA(n=3)	mouse	12 mice	W=0.9589,P=0.6102,W=0.9496,P=0.5675,W=0.8919,P=0.3600,W=0.9325,P=0.4982	F=0.8090,DFn=3,Dfd=8, P=0.5236	One-way ANOVA	F (3, 8) = 12.68	0.0021	**
		NR1$^{fl/fl}$ vs. SNS-NR1$^{-/-}$					Uncorrected Fisher's LSD		0.6359	ns
		NR1$^{fl/fl}$ vs. NR1$^{fl/fl}$+CFA					Uncorrected Fisher's LSD		0.0029	**
		NR1$^{fl/fl}$ vs. SNS-NR1$^{-/-}$+CFA					Uncorrected Fisher's LSD		0.1766	ns
		SNS-NR1$^{-/-}$ vs. NR1$^{fl/fl}$+CFA					Uncorrected Fisher's LSD		0.0015	**
		SNS-NR1$^{-/-}$ vs. SNS-NR1$^{-/-}$+CFA					Uncorrected Fisher's LSD		0.3511	ns
		NR1$^{fl/fl}$+CFA vs. SNS-NR1$^{-/-}$+CFA					Uncorrected Fisher's LSD		0.0005	***
		NR1$^{fl/fl}$(n=3), SNS-NR1$^{-/-}$(n=3), NR1$^{fl/fl}$+CFA(n=3), SNS-NR1$^{-/-}$+CFA(n=3)	mouse	12 mice	W=0.8532,P=0.2491,W=0.9695,P=0.6649,W=0.7921,P=0.0958,W=0.9675,P=0.6540	F=0.6249,DFn=3,Dfd=8, P=0.6188	One-way ANOVA	F (3, 8) = 13.74	0.0016	**
		NR1$^{fl/fl}$ vs. SNS-NR1$^{-/-}$					Uncorrected Fisher's LSD		0.0315	***
		NR1$^{fl/fl}$ vs. NR1$^{fl/fl}$+CFA					Uncorrected Fisher's LSD		0.1742	**

Fig5h-right	SK2/β-actin	$NR1^{fl/fl}$ vs. SNS-$NR1^{-/-}$+CFA					Uncorrected Fisher's LSD	0.0092	***
		SNS-$NR1^{-/-}$ vs. $NR1^{fl/fl}$+CFA					Uncorrected Fisher's LSD	0.428	ns
		SNS-$NR1^{-/-}$ vs. SNS-$NR1^{-/-}$+CFA					Uncorrected Fisher's LSD	0.6506	ns
		$NR1^{fl/fl}$+CFA vs. SNS-$NR1^{-/-}$+CFA					Uncorrected Fisher's LSD	0.2129	ns
Fig5j	Amplitude of AHP current(pA)	$NR1^{fl/fl}$ (n=5), SNS-$NR1^{-/-}$(n=7)	neuron	6 mice(5 slices for $NR1^{fl/fl}$ mice and 7 slices for SNS-$NR1^{-/-}$ mice)	W=0.9103,P=0.4696,W=0.9943,P=0.9923,W=0.8904,P=0.2765W=0.8488,P=0.1198	F=3.704,DFn=3,Dfd=20, P=0.0287	Brown-Forsythe ANOVA test	F*=17.59 (3.000, 8.414)	0.0006	***
		$NR1^{fl/fl}$-1st vs. SNS-$NR1^{-/-}$-1st					Unpaired t with Welch's correction	0.0148	*
		$NR1^{fl/fl}$-2nd vs. SNS-$NR1^{-/-}$-2nd					Unpaired t with Welch's correction	0.0066	**
Fig5l	PKG-I/β-actin	$NR1^{fl/fl}$(n=9), SNS-$NR1^{-/-}$(n=9), $NR1^{fl/fl}$+CFA(n=9), SNS-$NR1^{-/-}$+CFA(n=9)	mouse	36 mice	W=0.8874,P=0.1875,W=0.9665,P=0.8629,W=0.9519,P=0.7112,W=0.8843,P=0.1742	1.546 (3, 32), P=0.2216	One-way ANOVA	F (3, 32) = 4.982	0.006	**
		$NR1^{fl/fl}$ vs. SNS-$NR1^{-/-}$					Uncorrected Fisher's LSD	0.1084	ns
		$NR1^{fl/fl}$ vs. $NR1^{fl/fl}$-CFA					Uncorrected Fisher's LSD	0.0005	***
		SNS-$NR1^{-/-}$ vs. SNS-$NR1^{-/-}$-CFA					Uncorrected Fisher's LSD	0.9475	ns
		$NR1^{fl/fl}$-CFA vs. SNS-$NR1^{-/-}$-CFA					Uncorrected Fisher's LSD	0.0408	*
Fig5n	Normalized C-eEPSCs	Control(n=5), NMDA(n=5), cGMP(n=5), NMDA+cGMP(n=5)	neuron	4 mice(5 slices)	W=0.8810,P=0.3140,W=0.9168,P=0.5095,W=0.9390,P=0.6587,W=0.8895,P=0.3544	F=6.532,DFn=3,Dfd=16, P<0.0001	Brown-Forsythe ANOVA test	F*=49.72 (3.000, 6.181)	0.0001	***
		Control vs. NMDA					Unpaired t with Welch's correction	0.002	**
		Control vs. cGMP					Unpaired t with Welch's correction	0.0937	ns
		Control vs. NMDA+cGMP					Unpaired t with Welch's correction	0.0032	**
		NMDA vs. NMDA+cGMP					Unpaired t with Welch's correction	0.0002	***
		cGMP vs. NMDA+cGMP					Unpaired t with Welch's correction	0.0035	**

Fig5p	Normalized C-eEPSCs	PKGI$^{fl/fl}$Control(n=5), PKGI$^{fl/fl}$+NMDA(n=5), SNS-PKGI$^{-/-}$(n=5), SNS-PKGI$^{-/-}$+NMDA(n=5), SNS-PKGI$^{-/-}$	neuron	6 mice(10 slices)	Not all subgroups meets p > 0.05	F=2.113,DFn=4,Dfd=20, P=0.1169	Kruskal-Wallis *H* test	Kruskal-Wallis statistic=16.91	0.002	**
		PKGI$^{fl/fl}$Control vs. PKGI$^{fl/fl}$+NMDA					Uncorrected Dunn's test		0.0349	*
		PKGI$^{fl/fl}$ vs. SNS-PKGI$^{-/-}$					Uncorrected Dunn's test		>0.9999	ns
		PKGI$^{fl/fl}$+NMDA vs. SNS-PKGI$^{-/-}$+NMDA					Uncorrected Dunn's test		0.0034	**
		SNS-PKGI$^{-/-}$ vs. SNS-PKGI$^{-/-}$+NMDA					Uncorrected Dunn's test		0.4114	ns
		SNS-PKGI$^{-/-}$ vs. SNS-PKGI$^{-/-}$+NMDA+cGMP					Uncorrected Dunn's test		0.0654	ns
		SNS-PKGI$^{-/-}$+NMDA vs. SNS-PKGI$^{-/-}$+NMDA+cGMP					Uncorrected Dunn's test		0.3072	ns
Fig6b	BDNF/β-actin	NR1$^{fl/fl}$(n=3), SNS-NR1$^{-/-}$(n=3), NR1$^{fl/fl}$+CFA(n=3), SNS-NR1$^{-/-}$+CFA(n=3)	mouse	12 mice	W=0.8177,P=0.1574,W=0.7809,P=0.0697,W=0.9138,P=0.4310,W=0.9892,P=0.8014	F=0.60883,DFn=3,Dfd=8, P=0.6278	One-way ANOVA	F (3, 8) = 57.13	<0.0001	****
		NR1$^{fl/fl}$ vs. SNS-NR1$^{-/-}$					Uncorrected Fisher's LSD		0.9491	ns
		NR1$^{fl/fl}$ vs. NR1$^{fl/fl}$+CFA					Uncorrected Fisher's LSD		<0.0001	****
		SNS-NR1$^{-/-}$ vs. SNS-NR1$^{-/-}$+CFA					Uncorrected Fisher's LSD		0.3285	ns
		NR1$^{fl/fl}$l+CFA vs. SNS-NR1$^{-/-}$+CFA					Uncorrected Fisher's LSD		<0.0001	****
Fig6d	BDNF/β-actin	PKG$^{fl/fl}$(n=3), SNS-PKG$^{-/-}$(n=3), PKG$^{fl/fl}$+CFA(n=3), SNS-PKG$^{-/-}$ +CFA(n=3)	mouse	12 mice	W=0.9987,P=0.9101,W=0.8575,P=0.2608,W=0.8804,P=0.3257,W=0.9950,P=0.8654	F=1.300, DFn=3, Dfd=8, P=0.3395	One-way ANOVA	F (3, 8) = 14.35	0.0014	**
		PKG$^{fl/fl}$ vs. SNS-PKG$^{-/-}$					Uncorrected Dunn's test		0.4322	ns
		PKG$^{fl/fl}$ vs. PKG$^{fl/fl}$+CFA					Uncorrected Dunn's test		0.0009	***
		SNS-PKG$^{-/-}$ vs. SNS-PKG$^{-/-}$+CFA					Uncorrected Dunn's test		0.2083	ns
		PKG$^{fl/fl}$+CFA vs. SNS-PKG$^{-/-}$+CFA					Uncorrected Dunn's test		0.0018	**
		Basal(n=12), AP5(n=14), KO(n=12)	presynaptic terminal puncta	6 mice(8 slices)	W=0.9492,P=0.6250,W=0.9710,P=0.8899,W=0.8958,P=0.1400	F=1.220, DFn=2, Dfd=35, P=0.3075	One-way ANOVA	F (2, 35) = 5.852	0.0064	**

Fig6h	Relative fluorescence intensity	Basal vs. AP5					Uncorrected Fisher's LSD		0.0031	**
		Basal vs. KO					Uncorrected Fisher's LSD		0.0096	**
Fig6j	Amplitude of C-eEPSCs(pA)	Basal(n=8), NMDA(n=8), TrkB-IgG(n=7), TrkB-IgG + NMDA(n=7)	neuron	6 mice(15 slices)	W=0.8896,P=0.2322,W=0.8804,P=0.1900,W=0.9307,P=0.5573,W=0.9393,P=0.6328	F=0.2105, DFn=2, Dfd=26, P=0.8882	One-way ANOVA	F (3, 26) = 3.512	0.0292	*
		Basal vs. NMDA					Uncorrected Fisher's LSD		0.0306	*
		Basal vs. TrkB-IgG					Uncorrected Fisher's LSD		0.6225	ns
		Basal vs. TrkB-IgG + NMDA					Uncorrected Fisher's LSD		0.5863	ns
		NMDA vs. TrkB-IgG					Uncorrected Fisher's LSD		0.0118	*
		NMDA vs. TrkB-IgG + NMDA					Uncorrected Fisher's LSD		0.0105	*
		TrkB-IgG vs. TrkB-IgG + NMDA					Uncorrected Fisher's LSD		0.9596	ns
Fig6m	Relative amplitude of C-eEPSCs after NMDA	AAV-conRNA(n=5), AAV-shBDNF(n=7)	neuron	6 mice(12 slices)	W=0.9397,P=06640,W=0.9407,P=0.6451,	F=4.594, DFn=4, Dfd=6, P=0.0973	Unpaired t test	t=3.136, df=10	0.0106	*
Fig6p	Relative amplitude of C-eEPSCs after LFS	AAV-conRNA(n=5), AAV-shBDNF(n=5)	neuron	6 mice(10 slices)	W=0.9080,P=0.4557,W=0.8941,P=0.3782,	F=8.447, DFn=4, Dfd=4, P=0.0625	Unpaired t test	t=2.364, df=8	0.0457	*
Fig6q	SK2 / β-actin	Basal(n=4),BDNF(n=4)	mouse	8 mice	W=0.7971,P=0.0971,W=0.9899,P=0.9569	F=38.62,DFn=3,Dfd=3, P=0.0135	Unpaired t test with Welch's correction	t=4.595, df=3.155	0.0173	*
Fig6s	AHP current(pA)	1st(n=5), 1st+BDNF(n=5), 2nd(n=5), 2nd+BDNF(n=5)	neuron	3 mice(5 slices)	W=0.9103,P=0.4696,W=0.8987,P=0.4028,W=0.9943,P=0.9923,W=0.8767,P=0.2946	F=2.311, DFn=3, Dfd=16, P=0.1150	One-way ANOVA	F (3, 16) = 23.30	<0.0001	****
		1st vs. 1st+BDNF					Uncorrected Fisher's LSD		0.0002	***
		2nd vs. 2nd+BDNF					Uncorrected Fisher's LSD		<0.0001	****
		$NR1^{fl/fl}$ (n=8), SNS-$NR1^{-/-}$(n=8)	mouse	16 mice	Not all subgroups meets p > 0.05	F=2.898, DFn=5, Dfd=42, P=0.0246	Kruskal-Wallis *H* test	Kruskal-Wallis statistic=34.16	<0.0001	****
		Basal:$NR1^{fl/fl}$ vs. Basal:SNS-$NR1^{-/-}$					Uncorrected Dunn's test		0.8501	ns
		Basal:$NR1^{fl/fl}$ vs. 15 min:$NR1^{fl/fl}$					Uncorrected Dunn's test		0.0004	***

Fig7b	Mechanical response threshold(g)	Basal:NR1$^{fl/fl}$ vs. 15 min:SNS-NR1$^{-/-}$					Uncorrected Dunn's test		0.1132	ns
		Basal:NR1$^{fl/fl}$ vs. 40 min:NR1$^{fl/fl}$					Uncorrected Dunn's test		<0.0001	****
		Basal:NR1$^{fl/fl}$ vs. 40 min:SNS-NR1$^{-/-}$					Uncorrected Dunn's test		0.0856	ns
		Basal:SNS-NR1$^{-/-}$ vs. 15 min:NR1$^{fl/fl}$					Uncorrected Dunn's test		0.0002	***
		Basal:SNS-NR1$^{-/-}$ vs. 15 min:SNS-NR1$^{-/-}$					Uncorrected Dunn's test		0.0762	ns
		Basal:SNS-NR1$^{-/-}$ vs. 40 min:NR1$^{fl/fl}$					Uncorrected Dunn's test		<0.0001	****
		Basal:SNS-NR1$^{-/-}$ vs. 40 min:SNS-NR1$^{-/-}$					Uncorrected Dunn's test		0.0564	ns
		15 min:NR1$^{fl/fl}$ vs. 15 min:SNS-NR1$^{-/-}$					Uncorrected Dunn's test		0.0487	*
		15 min:NR1$^{fl/fl}$ vs. 40 min:NR1$^{fl/fl}$					Uncorrected Dunn's test		0.3976	ns
		15 min:NR1$^{fl/fl}$ vs. 40 min:SNS-NR1$^{-/-}$					Uncorrected Dunn's test		0.0664	ns
		15 min:SNS-NR1$^{-/-}$ vs. 40 min:NR1$^{fl/fl}$					Uncorrected Dunn's test		0.0048	**
		15 min:SNS-NR1$^{-/-}$ vs. 40 min:SNS-NR1$^{-/-}$					Uncorrected Dunn's test		0.8926	ns
		40 min:NR1$^{fl/fl}$ vs. 40 min:SNS-NR1$^{-/-}$					Uncorrected Dunn's test		0.0073	**
		NR1$^{fl/fl}$(n=10), SNS-NR1$^{-/-}$ (n=10)	mouse	20 mice	Not all subgroups meets p > 0.05	F=3.102, DFn=5, Dfd=54, P=0.0156	Kruskal-Wallis *H* test	Kruskal-Wallis statistic=39.87	<0.0001	****
		Basal:NR1$^{fl/fl}$ vs. Basal:SNS-NR1$^{-/-}$					Uncorrected Dunn's test		0.8612	ns
		Basal:NR1$^{fl/fl}$ vs. Cap:NR1$^{fl/fl}$					Uncorrected Dunn's test		<0.0001	****
		Basal:NR1$^{fl/fl}$ vs. Cap:SNS-NR1$^{-/-}$					Uncorrected Dunn's test		0.0227	*
		Basal:NR1$^{fl/fl}$ vs. Cap + AP5:NR1$^{fl/fl}$					Uncorrected Dunn's test		0.0031	**

FIg7d	Mechanical response threshold(g)	Basal:NR1$^{fl/fl}$ vs. Cap + AP5:SNS-NR1$^{-/-}$					Uncorrected Dunn's test		0.4334	ns
		Basal:SNS-NR1$^{-/-}$ vs. Cap:NR1$^{fl/fl}$					Uncorrected Dunn's test		<0.0001	****
		Basal:SNS-NR1$^{-/-}$ vs. Cap:SNS-NR1$^{-/-}$					Uncorrected Dunn's test		0.0141	*
		Basal:SNS-NR1$^{-/-}$ vs. Cap + AP5:NR1$^{fl/fl}$					Uncorrected Dunn's test		0.0017	**
		Basal:SNS-NR1$^{-/-}$ vs. Cap + AP5:SNS-NR1$^{-/-}$					Uncorrected Dunn's test		0.3379	ns
		Cap:NR1$^{fl/fl}$ vs. Cap:SNS-NR1$^{-/-}$					Uncorrected Dunn's test		0.0067	**
		Cap:NR1$^{fl/fl}$ vs. Cap + AP5:NR1$^{fl/fl}$					Uncorrected Dunn's test		0.042	*
		Cap:NR1$^{fl/fl}$ vs. Cap + AP5:SNS-NR1$^{-/-}$					Uncorrected Dunn's test		<0.0001	****
		Cap:SNS-NR1$^{-/-}$ vs. Cap + AP5:NR1$^{fl/fl}$					Uncorrected Dunn's test		0.4966	ns
		Cap:SNS-NR1$^{-/-}$ vs. Cap + AP5:SNS-NR1$^{-/-}$					Uncorrected Dunn's test		0.1347	ns
		Cap + AP5:NR1$^{fl/fl}$ vs. Cap + AP5:SNS-NR1$^{-/-}$					Uncorrected Dunn's test		0.0296	*
		NR1$^{fl/fl}$(n=4), SNS-NR1$^{-/-}$ (n=5)	mouse	9 mice	Not all subgroups meets p > 0.05		Friedman *M* test	χ2=20.167, df=1	<0.0001	****
		Basal:floxed vs. Basal:NR1 ko					Mann Whitney test		0.7937	ns
		Basal:floxed vs. 24h:floxed					Mann Whitney test		0.0286	*
		Basal:floxed vs. 72h:floxed					Mann Whitney test		0.0286	*
		Basal:floxed vs. 1w:floxed					Mann Whitney test		0.0286	*
		Basal:floxed vs. 2w:floxed					Mann Whitney test		0.0286	*
		Basal:floxed vs. 3w:floxed					Mann Whitney test		0.0286	*

		Basal:NR1 ko vs. 24h:NR1 ko					Mann Whitney test		>0.9999	ns
Fig7f-ipsilateral		Basal:NR1 ko vs. 72h:NR1 ko					Mann Whitney test		>0.9999	ns
		Basal:NR1 ko vs. 1w:NR1 ko					Mann Whitney test		0.5952	ns
		Basal:NR1 ko vs. 2w:NR1 ko					Mann Whitney test		0.3413	ns
		Basal:NR1 ko vs. 3w:NR1 ko					Mann Whitney test		0.0476	*
		24h:floxed vs. 24h:NR1 ko					Mann Whitney test		0.0159	†
		72h:floxed vs. 72h:NR1 ko					Mann Whitney test		0.0159	†
		1w:floxed vs. 1w:NR1 ko					Mann Whitney test		0.0159	†
		2w:floxed vs. 2w:NR1 ko					Mann Whitney test		0.0159	†
		3w:floxed vs. 3w:NR1 ko					Mann Whitney test		0.0079	††
		$NR1^{fl/fl}$(n=4), SNS-$NR1^{-/-}$ (n=5)	mouse	9 mice	Not all subgroups meets p > 0.05		Friedman M test	$\chi 2=20.167$, df=1	<0.0001	****
		Basal:floxed vs. Basal:NR1 ko					Mann Whitney test		0.5556	ns
		Basal:floxed vs. 24h:floxed					Mann Whitney test		0.0286	*
		Basal:floxed vs. 72h:floxed					Mann Whitney test		0.0286	*
		Basal:floxed vs. 1w:floxed					Mann Whitney test		0.0286	*
		Basal:floxed vs. 2w:floxed					Mann Whitney test		0.0286	*
		Basal:floxed vs. 3w:floxed					Mann Whitney test		0.0286	*
		Basal:NR1 ko vs. 24h:NR1 ko					Mann Whitney test		>0.9999	ns

Fig7f-contralateral		Basal:NR1 ko vs. 72h:NR1 ko					Mann Whitney test		>0.9999	ns
		Basal:NR1 ko vs. 1w:NR1 ko					Mann Whitney test		0.5952	ns
		Basal:NR1 ko vs. 2w:NR1 ko					Mann Whitney test		>0.9999	ns
		Basal:NR1 ko vs. 3w:NR1 ko					Mann Whitney test		>0.9999	ns
		24h:floxed vs. 24h:NR1 ko					Mann Whitney test		0.0159	†
		72h:floxed vs. 72h:NR1 ko					Mann Whitney test		0.0159	†
		1w:floxed vs. 1w:NR1 ko					Mann Whitney test		0.0159	†
		2w:floxed vs. 2w:NR1 ko					Mann Whitney test		0.0159	†
		3w:floxed vs. 3w:NR1 ko					Mann Whitney test		0.0159	†
Fig7g-left	Basal	Control (n=4), shRNA BDNF(n=4)	mouse	8 mice	Not all subgroups meets p > 0.05		Friedman M test	χ2=1.286, df=1	0.257	ns
Fig7g-middle	20 min after Cap	Control (n=4), shRNA BDNF(n=4)	mouse	8 mice	Not all subgroups meets p > 0.05		Friedman M test	χ2=6.000, df=1	0.014	*
Fig7g-right	40 min after Cap	Control (n=4), shRNA BDNF(n=4)	mouse	8 mice	Not all subgroups meets p > 0.05		Friedman M test	χ2=12, df=1	0.001	***
Fig8b	Mechanical response frequency (%)	AAV-EF1-DIO-NR1-3Flag-SNS-NR1-/- (n=7), AAV-EF1-DIO-NR1-3Flag-SNS Cre (n=7), AAV- EF1-DIO-3Flag-SNS-NR1-/- (n=5), AAV- EF1-DIO-3Flag-SNS-Cre (n=8)	mouse	27 mice	Not all subgroups meets p > 0.05		Friedman M test	χ2=6.896, df=3	0.075	ns
Fig8c	Mechanical response frequency (%)	AAV-EF1-DIO-NR1-3Flag-SNS Cre (n=7), AAV- EF1-DIO-3Flag-SNS-Cre (n=7)	mouse	14 mice	Not all subgroups meets p > 0.05		Friedman M test	χ2=1.636, df=1	0.201	ns
Fig8d	Mechanical response frequency (%)	AAV-EF1-DIO-NR1-3Flag-SNS-NR1-/- (n=6), AAV- EF1-DIO-3Flag-SNS-NR1-/- (n=5), AAV- EF1-DIO-3Flag-SNS-Cre (n=7)	mouse	18 mice	Not all subgroups meets p > 0.05		Friedman M test	χ^2=30.484,df=2	<0.0001	****
		Group A vs. Group B					Student-Newman-Keuls q test		<0.0001	****
		Group A vs. Group C					Student-Newman-Keuls q test		0.302	ns
		Group B vs. Group C					Student-Newman-Keuls q test		<0.0001	****

Fig8f		AAV-EF1-DIO-NR1-3Flag-SNS Cre (n=5), AAV- EF1-DIO-3Flag-SNS-Cre (n=5)	mouse	10 mice	Not all subgroups meets p > 0.05		Friedman M test	χ^2=0.333,df=1	0.564	ns
Fig8g		AAV-EF1-DIO-NR1-3Flag-SNS Cre (n=5), AAV- EF1-DIO-3Flag-SNS-Cre (n=5)	mouse	10 mice	Not all subgroups meets p > 0.05		Friedman M test	χ^2=6.250,df=1	0.012	*
Fig8h		AAV-EF1-DIO-NR1-3Flag-SNS Cre (n=6), AAV- EF1-DIO-3Flag-SNS-Cre (n=6)	mouse	12 mice	Not all subgroups meets p > 0.05		Friedman M test	χ^2=25.000,df=1	<0.0001	****
Fig8i	Mechanical response threshold(g)	Basal(n=13), Cap(n=13), 2μM(n=5), 10μM(n=8)	mouse	26 mice	W=0.9499,P=0.5966,W=0.9715,P=0.9114,W=0.9440,P=0.6946,W=0.9862,P=0.9868	F=1.295, DFn=3, Dfd=35, P=0.2915	One-way ANOVA	F (3, 35) = 39.67	<0.0001	****
		Basal vs. Cap					Uncorrected Fisher's LSD		<0.0001	****
		Basal vs. 2μM					Uncorrected Fisher's LSD		0.009	**
		Basal vs. 10μM					Uncorrected Fisher's LSD		0.546	ns
		Cap vs. 2μM					Uncorrected Fisher's LSD		<0.0001	****
		Cap vs. 10μM					Uncorrected Fisher's LSD		<0.0001	****
		2μM vs. 10μM					Uncorrected Fisher's LSD		0.0455	*
sup. Fig1b	NR1-expressing cells (% total)	12-15 DRG sections each	section	3 mice	Not all subgroups meets p > 0.05	F=7.620, DFn=5, Dfd=117, P<0.0001	Kruskal-Wallis *H* test	Kruskal-Wallis statistic=100.1	<0.0001	****
		<10μm-NR1$^{fl/fl}$ vs. <10μm-SNS NR1$^{-/-}$							0.0186	*
		10-20μm-NR1$^{fl/fl}$ vs. 10-20μm-SNS NR1$^{-/-}$							0.0014	**
		10-20μm-NR1$^{fl/fl}$ vs. ≥20μm-NR1$^{fl/fl}$							0.2322	ns
		≥20μm-NR1$^{fl/fl}$ vs. ≥20μm-SNS NR1$^{-/-}$							0.3134	ns
sup. Fig1c	% of cells expressing NR1 in a given population	12-15 DRG sections each	section	3 mice	Not all subgroups meets p > 0.05	F=0.7939, DFn=5, Dfd=75, P=0.7223	Kruskal-Wallis *H* test	Kruskal-Wallis statistic=57.82	<0.0001	****
		IB_4-NR1$^{fl/fl}$ vs. IB_4-SNS NR1$^{-/-}$							0.0003	***
		CGRP-NR1$^{fl/fl}$ vs. CGRP-SNS NR1$^{-/-}$							<0.0001	****

		NF200-NR1$^{fl/fl}$ vs. NF200-SNS NR1$^{-/-}$							0.9465	ns
sup.Fig1d	The fluorescence intensity of NR1 inspinal dorsal horn	NR1$^{fl/fl}$(n=6), SNS-NR1$^{-/-}$(n=6)	section	6 mice	W=0.9608,P=0.8262,W=0.9271,P=0.5581	F=4.088, DFn=5, Dfd=5, P=0.1484	Unpaired t test	t=2.318, df=10	0.0429	*
sup.Fig1e	NR1/β-actin	NR1$^{fl/fl}$(n=4), SNS-NR1$^{-/-}$(n=4)	mouse	8 mice	W=0.8639,P=0.2744,W=0.9028,P=0.4450	F=3.010, DFn=3, Dfd=3, P=0.3897	Unpaired t test	t=11.16, df=6	<0.0001	****
sup.Fig1f	NR1/β-actin	NR1$^{fl/fl}$(n=3), SNS-NR1$^{-/-}$(n=3)	mouse	6 mice	W=0.9976,P=0.9058,W=0.9689,P=0.6612	F=34.08, DFn=2, Dfd=2, P=0.0570	Unpaired t test	t=1.153, df=4	0.3131	ns
sup. Fig1g	NR1/β-actin	NR1$^{fl/fl}$(n=3), SNS-NR1$^{-/-}$(n=3)	mouse	6 mice	Not all subgroups meets p > 0.05	F=2.601, DFn=2, Dfd=2, P=0.5554	Mann Whitney U test		0.7	ns
sup. Fig2c	Amplitude of NMDA current (pA)	NR1$^{fl/fl}$(n=5), SNS-NR1$^{-/-}$(n=5)	neuron	6 mice(10 slices)	W=0.8785,P=0.3024,W=0.90869,P=0.4595	F=224.0, DFn=4, Dfd=4, P=0.0001	Unpaired t test with Welch's correction	t=3.103, df=4.036	0.0357	*
sup. Fig2g	NMDA-induced current (pA)	NR1$^{fl/fl}$(n=6), SNS-NR1$^{-/-}$(n=5)	neuron	6 mice(11 slices)	W=0.9529,P=0.7639,W=0.9160,P=0.5042	F=7.291, DFn=4, Dfd=5, P=0.0514	Unpaired t test	t=0.1608, df=9	0.8758	ns
sup. Fig2i	IB4/CGRP-expressing cells (% total)	IB$_4$-NR1^{fl}/^{fl}(n=10), IB$_4$-SNS NR1$^{-/-}$(n=10),CGRP-NR1$^{fl/fl}$(n=9), CGRP-SNS NR1$^{-/-}$(n=8),	section	6 mice	W=0.9624,P=0.8128,W=0.7523,P=0.0038,W=0.8460,P=0.0673,W=0.9471,P=0.6816	F=2.173, DFn=3, Dfd=33, P=0.1098	One-way ANOVA	F (3, 33) = 0.2128	0.8868	ns
sup. Fig2j	Fluorescence intensity of IB4 in SC	NR1$^{fl/fl}$(n=6), SNS-NR1$^{-/-}$(n=6)	section	6 mice	W=0.9993,P=0.9501,W=0.8151,P=0.1510	F=1.058, DFn=5, Dfd=5, P=0.9521	Unpaired t test	t=0.3535, df=10	0.7311	ns
	Fluorescence intensity of CGRP in SC	NR1$^{fl/fl}$(n=6), SNS-NR1$^{-/-}$(n=6)	section	6 mice	W=0.9630,P=0.8289,W=0.8430,P=0.17873	F=1.655, DFn=4, Dfd=4, P=0.6375	Unpaired t test	t=0.4808, df=8	0.6435	ns
sup.Fig3b	NMDA-induced current (pA)	Control(n=3),MK801(n=3)	neuron	2 mice(3 slices)	W=0.9993,P=0.9501,W=0.8151,P=0.1510	F=35.80, DFn=2, Dfd=2, P=0.0543	Unpaired t test	t=7.553, df=4	0.0016	**
sup.Fig3d	Normalized C-eEPSCs （%）	Basal(n=5),30min after LFS(n=5)	neuron	3 mice(5 slices)	W=0.9782,P=0.9248,W=0.8331,P=0.8331	no need for Paired t tests	Paired t test	t=0.7544, df=4	0.4926	ns
sup.Fig3f	Normalized C-eEPSCs （%）	Basal(n=5),30min after LFS(n=5)	neuron	3 mice(5 slices)	W=0.8346,P=0.1506,W=0.9631,P=0.8292	no need for Paired t tests	Paired t test	t=0.6853, df=4	0.5308	ns
sup.Fig3h	Normalized C-eEPSCs （%）	Basal(n=5),30min after LFS(n=5)	neuron	3 mice(5 slices)	W=0.8837,P=0.3266,W=0.8111,P=0.0994	no need for Paired t tests	Paired t test	t=6.421, df=4	0.003	**
sup.Fig4a-left	PPR	Before CFS(n=13),After CFS(n=13)	neuron	9 mice(13 slices)	Not all subgroups meets p > 0.05	F=1.058, DFn=12, Dfd=12, P=0.2194	Mann Whitney U test	Mann-Whitney U=42	0.0295	*
sup.Fig4a-middle	PPR	Before CFS(n=7),After CFS(n=7)	neuron	5 mice(7 slices)	W=0.9292,P=0.5442,W=0.8676,P=0.1770	F=1.751, DFn=6, Dfd=6, P=0.3458	Paired t test	t=4.945, df=6	0.0026	**
sup.Fig4a-right	PPR	Before CFS(n=11),After CFS(n=11)	neuron	8 mice(11 slices)	W=0.9614,P=0.7888,W=0.9278,P=0.3895	F=1.395, DFn=10, Dfd=10, P=0.6086	Paired t test	t=5.685, df=10	0.002	***
sup.Fig4c-left	PPR	Before CFS(n=9),After CFS(n=9)	neuron	6 mice(9 slices)	Not all subgroups meets p > 0.05	F=1.706, DFn=8, Dfd=8, P=0.4668	Mann Whitney U test	Mann-Whitney U=35	0.6665	ns

sup.Fig4c-middle	PPR	Before CFS(n=8),After CFS(n=8)	neuron	5 mice(8 slices)	W=0.8857,P=0.2134,W=0.8353,P=0.0674	F=1.076, DFn=7, Dfd=7, P=0.9251	Unpaired t test	t=1.111, df=14	0.2855	ns
sup.Fig4c-right	PPR	Before CFS(n=13),After CFS(n=13)	neuron	10 mice(13 slices)	W=0.9540,P=0.6605,W=0.9330,P=0.3724	F=1.188, DFn=12, Dfd=12, P=0.7706	Unpaired t test	t=0.7626, df=24	0.4531	ns
sup.Fig5c	Vertical distance to the diagonal	NR1$^{fl/fl}$(n=9), SNS-NR1$^{-/-}$(n=6)	neuron	6 mice(15 slices)	Not all subgroups meets p > 0.05	F=982.9, DFn=8, Dfd=5, P<0.0001	Mann Whitney U test	Mann-Whitney U=0	0.0004	***
sup.Fig5f	Vertical distance to the diagonal	NR1$^{fl/fl}$(n=12), SNS-NR1$^{-/-}$(n=8)	neuron	7 mice(20 slices)	W=0.9372,P=0.4627,W=0.8640,P=0.1317	F=18.33, DFn=11, Dfd=7, P=0.0008	Unpaired t test with Welch's correction	t=5.340, df=12.74	0.0001	***
sup.Fig5i	Vertical distance to the diagonal	NR1$^{fl/fl}$(n=8), SNS-NR1$^{-/-}$(n=6)	neuron	5 mice(14 slices)	Not all subgroups meets p > 0.05	F=1180, DFn=7, Dfd=5, P<0.0001	Mann Whitney U test	Mann-Whitney U=1	0.0013	**
sup.Fig5l	Vertical distance to the diagonal	NR1$^{fl/fl}$(n=10), SNS-NR1$^{-/-}$(n=8)	neuron	6 mice(18 slices)	Not all subgroups meets p > 0.05	F=13.26, DFn=9, Dfd=7, P=0.0026	Mann Whitney U test	Mann-Whitney U=11	0.0085	**
sup.Fig6a-left	PPR	WT(n=6), AP5(n=6)	neuron	4 mice(6 slices)	W=0.9583,P=0.8063,W=0.8846,P=0.2907	F=5.596, DFn=5, Dfd=5, P=0.0819	Paired t test	t=3.920, df=5	0.0112	*
sup.Fig6a-right	PPR	KO(n=6), AP5(n=6)	neuron	4 mice(6 slices)	W=0.9725,P=0.9089,W=0.8998,P=0.3728	F=1.665, DFn=5, Dfd=5, P=0.5894	Paired t test	t=0.8917, df=5	0.4134	ns
sup.Fig6b-left	PPR	NR1$^{fl/fl}$-CFA(n=5), AP5(n=5)	neuron	3 mice(5 slices)	W=0.9431,P=0.6881,W=0.8869,P=0.3419	F=5.192, DFn=4, Dfd=4, P=0.1397	Paired t test	t=3.767, df=4	0.0197	*
sup.Fig6b-right	PPR	SNS-NR1$^{-/-}$-CFA(n=6), AP5(n=6)	neuron	4 mice(5 slices)	W=0.9258,P=0.5481,W=0.9088,P=0.4285	F=1.662, DFn=5, Dfd=5, P=0.5909	Paired t test	t=0.7207, df=5	0.5034	ns
sup.Fig7a-left	Freq. of mEPSCs (Hz)	Ctrl(n=7),AP5(n=7)	neuron	3 mice(7 slices)	W=0.8086,P=0.0498,W=0.9229,P=0.4932	no need for Paired t tests	Paired t test	t=3.515, df=6	0.0126	*
sup.Fig7a-right	Amp. of mEPSCs (pA)	Ctrl(n=7),AP5(n=7)	neuron	3 mice(7 slices)	W=0.9638,P=0.8506,W=0.9525,P=0.7524	no need for Paired t tests	Paired t test	t=0.8767, df=6	0.4144	ns
sup.Fig7b-left	Freq. of mEPSCs (Hz)	Ctrl(n=5),AP5(n=5)	neuron	3 mice(5 slices)	W=0.9323,P=0.6122,W=0.8520,P=0.2008	no need for Paired t tests	Paired t test	t=88201 df=4	0.4582	ns
sup.Fig7b-right	Amp. of mEPSCs (pA)	Ctrl(n=5),AP5(n=5)	neuron	3 mice(5 slices)	W=0.7533,P=0.0319,W=0.8838,P=0.3269	no need for Paired t tests	Paired t test	t=1.624, df=4	0.1796	ns
sup.Fig7c-left	Freq. of mEPSCs (Hz)	Ctrl(n=7),AP5(n=7)	neuron	3 mice(7 slices)	W=0.9249,P=0.5083,W=0.8634,P=0.1625	no need for Paired t tests	Paired t test	t=4.894, df=6	0.0027	**
sup.Fig7c-right	Amp. of mEPSCs (pA)	Ctrl(n=7),AP5(n=7)	neuron	3 mice(7 slices)	W=0.9024,P=0.3457,W=0.9512,P=0.7402	no need for Paired t tests	Paired t test	t=1.159, df=6	0.2905	ns
sup.Fig7d-left	Freq. of mEPSCs (Hz)	Ctrl(n=5),AP5(n=5)	neuron	3 mice(5 slices)	W=0.8545,P=0.2090,W=0.8839,P=0.3272	no need for Paired t tests	Paired t test	t=1.870, df=4	0.1348	ns
sup.Fig7d-right	Amp. of mEPSCs (pA)	Ctrl(n=5),AP5(n=5)	neuron	3 mice(5 slices)	W=0.8646,P=0.2453,W=0.9640,P=0.8353	no need for Paired t tests	Paired t test	t=0.2133, df=4	0.8415	ns

sup.Fig9b	SK2/ β-actin	NR1$^{fl/fl}$(n=6), SNS-NR1$^{-/-}$(n=6)	mouse	12 mice	W=0.8709,P=0.2300,W=0.9000,P=0.3738	F=2.881, DFn=5, Dfd=5, P=0.2695	Unpaired t test	t=4.461, df=10	0.0012	**
sup.Fig9h-left	p-NR1 / NR1	NR1$^{fl/fl}$(n=6), SNS-NR1$^{-/-}$(n=6),NR1$^{fl/fl}$CFA(n=6), SNS-NR1$^{-/-}$CFA(n=6),	mouse	18 mice	W=0.9235,P=0.5306,W=0.8978,P=0.3612,W=0.9394,P=0.6546,W=0.9395,P=0.6555	3.971 (3, 20),p=0.0226	Brown-Forsythe ANOVA test	F*=5.263 (3.000, 7.212)	0.0312	*
		NR1$^{fl/fl}$ vs. SNS-NR1$^{-/-}$					Unpaired t with Welch's correction		0.9444	ns
		NR1$^{fl/fl}$ vs. NR1$^{fl/fl}$CFA					Unpaired t with Welch's correction		<0.0001	****
		NR1$^{fl/fl}$ vs. SNS-NR1$^{-/-}$CFA					Unpaired t with Welch's correction		0.8037	ns
		SNS-NR1$^{-/-}$ vs. NR1$^{fl/fl}$CFA					Unpaired t with Welch's correction		0.0535	ns
		SNS-NR1$^{-/-}$ vs. SNS-NR1$^{-/-}$CFA					Unpaired t with Welch's correction		0.9721	ns
		NR1$^{fl/fl}$CFA vs. SNS-NR1$^{-/-}$CFA					Unpaired t with Welch's correction		0.0004	***
sup.Fig9h-right	SK2 / β-actin	NR1$^{fl/fl}$(n=5), SNS-NR1$^{-/-}$(n=6),NR1$^{fl/fl}$CFA(n=4), SNS-NR1$^{-/-}$CFA(n=6),	mouse	20 mice	W=0.9030,P=0.4269,W=0.8017,P=0.0609,W=0.8743,P=0.3150,W=0.9869,P=0.9804	2.372 (3, 17),P=0.1064	One-way ANOVA	F (3, 17) = 83.79	<0.0001	****
		NR1$^{fl/fl}$ vs. SNS-NR1$^{-/-}$					Uncorrected Fisher's LSD		<0.0001	****
		NR1$^{fl/fl}$ vs. NR1$^{fl/fl}$-CFA					Uncorrected Fisher's LSD		0.0313	*
		NR1$^{fl/fl}$ vs. SNS-NR1$^{-/-}$-CFA					Uncorrected Fisher's LSD		<0.0001	****
		SNS-NR1$^{-/-}$ vs. NR1$^{fl/fl}$-CFA					Uncorrected Fisher's LSD		<0.0001	****
		SNS-NR1$^{-/-}$ vs. SNS-NR1$^{-/-}$-CFA					Uncorrected Fisher's LSD		<0.0001	****
		NR1$^{fl/fl}$-CFA vs. SNS-NR1$^{-/-}$-CFA					Uncorrected Fisher's LSD		0.0001	***
		NR1$^{fl/fl}$(n=3), SNS-NR1$^{-/-}$(n=3),NR1$^{fl/fl}$CFA(n=3), SNS-NR1$^{-/-}$CFA(n=3),	mouse	12 mice	W=0.9954,P=0.8701,W=0.9559,P=0.5959,W=0.9972,P=0.8996,W=0.8929,P=0.3631	0.1614 (3, 8),P=0.9194	One-way ANOVA	F (3, 8) = 10.09	0.0043	**
		NR1$^{fl/fl}$ vs. SNS-NR1$^{-/-}$					Uncorrected Fisher's LSD		0.5138	ns
		NR1$^{fl/fl}$ vs. NR1$^{fl/fl}$-CFA					Uncorrected Fisher's LSD		0.0047	**

sup.Fig9j	PKG-I / β-actin	NR1$^{fl/fl}$ vs. SNS-NR1$^{-/-}$-CFA					Uncorrected Fisher's LSD		0.1903	ns
		SNS-NR1$^{-/-}$ vs. NR1$^{fl/fl}$-CFA					Uncorrected Fisher's LSD		0.0126	*
		SNS-NR1$^{-/-}$ vs. SNS-NR1$^{-/-}$-CFA					Uncorrected Fisher's LSD		0.0674	ns
		NR1$^{fl/fl}$-CFA vs. SNS-NR1$^{-/-}$-CFA					Uncorrected Fisher's LSD		0.0007	***
sup.Fig10b	BDNF / β-actin	NR1$^{fl/fl}$(n=5), SNS-NR1$^{-/-}$ (n=6),NR1$^{fl/fl}$CFA(n=4), SNS-NR1$^{-/-}$ CFA(n=5),	mouse	20 mice	W=0.8564,P=0.2158,W=0.8680,P=0.2183,W=0.9949,P=0.9810,W=0.8221,P=0.1212	3.422 (3, 16), P=0.0428	Brown-Forsythe ANOVA test	F*=6.888 (3.000, 7.340)	0.0155	*
		NR1$^{fl/fl}$ vs. SNS-NR1$^{-/-}$					Unpaired t with Welch's correction		0.2541	ns
		NR1$^{fl/fl}$ vs. NR1$^{fl/fl}$-CFA					Unpaired t with Welch's correction		0.0122	*
		NR1$^{fl/fl}$ vs. SNS-NR1$^{-/-}$-CFA					Unpaired t with Welch's correction		0.1578	ns
		SNS-NR1$^{-/-}$ vs. NR1$^{fl/fl}$-CFA					Unpaired t with Welch's correction		0.0174	*
		SNS-NR1$^{-/-}$ vs. SNS-NR1$^{-/-}$-CFA					Unpaired t with Welch's correction		0.2651	ns
		NR1$^{fl/fl}$-CFA vs. SNS-NR1$^{-/-}$-CFA					Unpaired t with Welch's correction		0.0357	*
sup.Fig10d	BDNF / β-actin	NR1$^{fl/fl}$(n=6), SNS-NR1$^{-/-}$ (n=6),NR1$^{fl/fl}$CFA(n=6), SNS-NR1$^{-/-}$ CFA(n=6),	mouse	12 mice	W=0.8239,P=0.0954,W=0.9449,P=0.6990,W=0.8739,P=0.2423,W=0.8145,P=0.0790	11.11 (3, 20), P=0.0002	One-way ANOVA	F (3, 20) = 5.562	0.0061	**
		PKG-I$^{fl/fl}$ vs. SNS-PKG-I$^{-/-}$					Uncorrected Fisher's LSD		0.3773	ns
		PKG-I$^{fl/fl}$ vs. PKG-1$^{fl/fl}$-CFA					Uncorrected Fisher's LSD		0.0009	***
		PKG-I$^{fl/fl}$ vs. SNS-PKG-I$^{-/-}$-CFA					Uncorrected Fisher's LSD		0.1644	ns
		SNS-PKG-I$^{-/-}$ vs. PKG-1$^{fl/fl}$-CFA					Uncorrected Fisher's LSD		0.0072	**
		SNS-PKG-I$^{-/-}$ vs. SNS-PKG-I$^{-/-}$-CFA					Uncorrected Fisher's LSD		0.5948	ns
		PKG-1$^{fl/fl}$-CFA vs. SNS-PKG-I$^{-/-}$-CFA					Uncorrected Fisher's LSD		0.0234	*

sup.Fig10j	Relative fluorescence intensity	SNS-Cre(n=8), SNS-NR1$^{-/-}$(n=7)	presynaptic terminal puncta	5 mice(9 slices)	W=0.9540,P=0.7511,W=0.7511,P=0.8120	F=1.721, DFn=6, Dfd=7, P=0.4932	Unpaired t test	t=3.137, df=13	0.0079	**
sup.Fig11d	BDNF/ β-actin	AAV-conRNA(n=4), AAV-shRNA BDNF (n=4)	mouse	8 mice	W=0.9125,P=0.4959,W=0.9597,P=0.7769	F=74.65, DFn=3, Dfd=3, P=0.0051	Unpaired t test with Welch's correction	t=5.239, df=3.080	0.0127	*
sup.Fig12a	Mechanical response threshold (g)	NR1$^{fl/fl}$(n=10), SNS-NR1$^{-/-}$(n=11)	mouse	21 mice	W=0.9140,P=0.3099,W=0.8990,P=0.1798	F=1.486, DFn=9, Dfd=10, P=0.5449	Unpaired t test	t=1.264, df=19	0.2215	ns
sup.Fig12b	Thermal response latency (g)	NR1$^{fl/fl}$(n=10), SNS-NR1$^{-/-}$(n=10)	mouse	21 mice	W=0.8875,P=0.2815,W=0.9102,P=0.2825	F=1.486, DFn=9, Dfd=9, P=0.5629	Unpaired t test	t=0.1725, df=18	0.8649	ns
sup.Fig12c	Response duration (s)	NR1$^{fl/fl}$(n=9), SNS-NR1$^{-/-}$(n=10)	mouse	21 mice	W=0.9048,P=0.1590,W=0.8994,P=0.2160	F=1.361, DFn=8, Dfd=9, P=0.6527	Unpaired t test	t=0.9069, df=17	0.3772	ns
