# Supplementary material for: Presynaptic NMDARs on spinal nociceptor terminals state-dependently modulate synaptic transmission and pain
Source: Nat Commun. 2022 Feb 7;13:728. doi: 10.1038/s41467-022-28429-y (PMC8821657; doi:10.1038/s41467-022-28429-y)
Supplement: Supplementary file 3 — Reporting Summary [file 41467_2022_28429_MOESM3_ESM.pdf]

nature portfolio

Corresponding author(s): Ceng Luo, DBPR (NCOMMS-20-19861B-Z)

Last updated by author(s): Nov 19, 2021

# Reporting Summary

Nature Portfolio wishes to improve the reproducibility of the work that we publish. This form provides structure for consistency and transparency in reporting. For further information on Nature Portfolio policies, see our Editorial Policies and the Editorial Policy Checklist.

## Statistics

For all statistical analyses, confirm that the following items are present in the figure legend, table legend, main text, or Methods section.

| n/a | Confirmed | |
|---|---|---|
| ☐ | ☒ | The exact sample size ($n$) for each experimental group/condition, given as a discrete number and unit of measurement |
| ☐ | ☒ | A statement on whether measurements were taken from distinct samples or whether the same sample was measured repeatedly |
| ☐ | ☒ | The statistical test(s) used AND whether they are one- or two-sided<br>*Only common tests should be described solely by name; describe more complex techniques in the Methods section.* |
| ☐ | ☒ | A description of all covariates tested |
| ☐ | ☒ | A description of any assumptions or corrections, such as tests of normality and adjustment for multiple comparisons |
| ☐ | ☒ | A full description of the statistical parameters including central tendency (e.g. means) or other basic estimates (e.g. regression coefficient) AND variation (e.g. standard deviation) or associated estimates of uncertainty (e.g. confidence intervals) |
| ☐ | ☒ | For null hypothesis testing, the test statistic (e.g. $F$, $t$, $r$) with confidence intervals, effect sizes, degrees of freedom and $P$ value noted<br>*Give P values as exact values whenever suitable.* |
| ☒ | ☐ | For Bayesian analysis, information on the choice of priors and Markov chain Monte Carlo settings |
| ☒ | ☐ | For hierarchical and complex designs, identification of the appropriate level for tests and full reporting of outcomes |
| ☒ | ☐ | Estimates of effect sizes (e.g. Cohen's $d$, Pearson's $r$), indicating how they were calculated |

*Our web collection on statistics for biologists contains articles on many of the points above.*

## Software and code

Policy information about availability of computer code

| | |
|---|---|
| Data collection | Softwares used to collect the data in this study include CLampex 9.2, Olympus Fluoview version 3.1 etc. Specifically, Clampex 9.2 was used for eletrophysiological data acquisition. Confocal images were collected with Olympus Fluoview version 3.1 in Olympus FV1200 microscope. Electron micrographs were captured with a Gatan digital 904 camera and its application software (832 SC1000). |
| Data analysis | Softwares used to analyze the data in this study include Microsoft Excel, GraphPad, Clampfit, ImageJ, Matlab. Specifically, the western images were quantified using ImageJ 1.50d software. Clampfit 10.6 was used for eletrophysiological data off-line analysis. Matlab 2012 was used to analyze calcium imaging data. Data were graphed and analyzed using Graphpad software Prism 8.0, Microsoft Excel 2013. All statistical analyses were performed in Prism 8.0 and SPSS 21.0. |

For manuscripts utilizing custom algorithms or software that are central to the research but not yet described in published literature, software must be made available to editors and reviewers. We strongly encourage code deposition in a community repository (e.g. GitHub). See the Nature Portfolio guidelines for submitting code & software for further information.

## Data

Policy information about availability of data

All manuscripts must include a data availability statement. This statement should provide the following information, where applicable:

- Accession codes, unique identifiers, or web links for publicly available datasets
- A description of any restrictions on data availability
- For clinical datasets or third party data, please ensure that the statement adheres to our policy

All data generated or analysed during this study are included in the main text and supplementary information files, and are available from the corresponding author upon reasonable request. Source data are provided with this paper. The source data underlying Figs. 1e, f, h, k; 2b, d, f, h, k; 3c, e-i; 4d, g; 5b, d, f, h, j, l, n, p; 6b, d,

h, j, m,p, q, s; 7b, d, f,g; 8b-d, f-i; Supplementary Figs. 1b-g; 2c, g, i, j; 3b, d, f, h; 4a, c; 5c, f, i, l; 6a, b; 7a-d; 9b, h, j; 10b, d, j; 11d; 12a-c are available as a Source Data file.

# Field-specific reporting

Please select the one below that is the best fit for your research. If you are not sure, read the appropriate sections before making your selection.

☒ Life sciences ☐ Behavioural & social sciences ☐ Ecological, evolutionary & environmental sciences

For a reference copy of the document with all sections, see nature.com/documents/nr-reporting-summary-flat.pdf

# Life sciences study design

All studies must disclose on these points even when the disclosure is negative.

| | |
|---|---|
| Sample size | Sample size for each experiment is clearly indicated in the figure legends for each experiment. The sample size was chosen based on previous experience for each experiment to yield high power to detect specific effects. PMID: 34156983, PMID: 16778058, PMID: 32773598, PMID: 31937758. No statistical methods were used to predetermine sample size. |
| Data exclusions | The sites of virus injection and expression were confirmed at the end of each experiment, animals displaying incorrect expression sites were excluded from analysis. For electrophysiological recordings, neurons were excluded when the resting membrane potential were positive than -55 mV and action potentials did not have overshoot or the series resistance changed by >20%. |
| Replication | All experiments were reliably reproduced in independent cells from independent mice (in vitro electrophysiology, calcium imaging) or mice (behaviour, immunofluorescence, western blot). Numbers of replicates (n) are indicated in the figure legends and Supplementary Table 2 for detailed statistical information. |
| Randomization | Animals from the littermates were used to decrease variance in age and rearing. Groups were randomized and mice were allocated to experimental groups by a researcher different from the experimentor. |
| Blinding | Experimentor was blinded to the identity of mice being analyzed in behavioral tests, electrophysiological recording, calcium imaging experiments and immunohistochemical staining experiments. |

# Reporting for specific materials, systems and methods

We require information from authors about some types of materials, experimental systems and methods used in many studies. Here, indicate whether each material, system or method listed is relevant to your study. If you are not sure if a list item applies to your research, read the appropriate section before selecting a response.

## Materials & experimental systems

| n/a | Involved in the study | |
|---|---|---|
| ☐ | ☒ | Antibodies |
| ☒ | ☐ | Eukaryotic cell lines |
| ☒ | ☐ | Palaeontology and archaeology |
| ☐ | ☒ | Animals and other organisms |
| ☒ | ☐ | Human research participants |
| ☒ | ☐ | Clinical data |
| ☒ | ☐ | Dual use research of concern |

## Methods

| n/a | Involved in the study | |
|---|---|---|
| ☒ | ☐ | ChIP-seq |
| ☒ | ☐ | Flow cytometry |
| ☒ | ☐ | MRI-based neuroimaging |

## Antibodies

| | |
|---|---|
| Antibodies used | The following primary antibodies were used: goat anti-NR1 antibody (Santa Cruz, 1:100, sc-31669), rabbit anti-NR1 antibody (Abcam, 1:500, ab109182), biotinylated griffonia simplicifolia lectin I, Isolectin B4 (vector laboratories, 1:200, B-1205), isolectin GS-IB4 from Griffonia simplicifolia, Alexa Fluor® 488 conjugate (Molecular Probes, Invitrogen, 10 microg/ml, I21411), rabbit anti-CGRP (Calbiochem, 1:500, PC250L ), mouse anti-NF200 (Sigma-Aldrich, 1:3000, N2912), rabbit anti-PSD-95 (Abcam, 1:200, ab18258), rabbit anti-synaptophysin antibody (Abcam, 1:200, ab32127), goat anti-GFP antibody (Rockland, 1:300, RK-600-102-215), rabbit anti-RFP antibody (Abcam, 1:300, ab62341), anti-Flag (Abbkine, 1:500, 1B10), rabbit anti-NR1 (Abcam, 1:1000, ab109182), goat anti-NR1 (Santa Cruz, 1:100, sc-1467), rabbit anti-phosphoNR1 (Santa Cruz, 1:1000, sc-31669), rabbit anti-SK2, (Alomone, 1:1000, APC-028), rabbit anti-BDNF (Novus, 1:500, NB100-98682), rabbit anti-PKG-I (Gift from Prof. Robert Feil), rabbit anti-beta-actin (HuaBio, R1207-1). The following secondary antibodies were used: anti-rabbit IgG, HRP-linked antibody (Cell Signaling Technology, 1: 1000, 7074), donkey anti-Goat IgG H&L, Alexa Fluor 488, (Abcam, 1:500, ab150129), goat anti-rabbit IgG (H+L), Alexa Fluor 488 (Invitrogen, 1:500, A11034 ), donkey anti-mouse IgG (H+L), Alexa Fluor 488 (Invitrogen, 1:500, A21202), donkey anti-mouse IgG (H+L), Alexa Fluor 594 (Invitrogen, 1:500, A21203), donkey anti-rabbit IgG H&L, Alexa Fluor 594 (Abcam, 1:500, ab150076), donkey anti-goat IgG (H+L), Alexa Fluor 594 (Invitrogen, 1:500, A11058). |
| Validation | The antibody was optimized and validated (i.e assay and species) by the company. |

## Animals and other organisms

Policy information about studies involving animals; ARRIVE guidelines recommended for reporting animal research

| | |
|---|---|
| Laboratory animals | Adult mice with females and males (8-14 weeks) were used in the study. We used mixed mice with identical number of females and males in each group to minimize the possible gender difference. The following strains were used: C57Bl6 wild-type mice, NR1fl/fl mice, SNS-NR1-/- mice, PKG-Ifl/fl mice, SNS-PKG-I-/- mice. Mice were housed up to 5 per cage and maintained on a 12 hr light/dark cycle with ad libitum access to food and water under the ambient temperature at 22-26 degree and humidity at 40-70%. |
| Wild animals | No wild animals were used. |
| Field-collected samples | No field-collected samples were used. |
| Ethics oversight | All experimental protocols were approved by Institutional Animal Care and Use Committee of the Fourth Military Medical University (FMMU). |

Note that full information on the approval of the study protocol must also be provided in the manuscript.
