# Supplementary material for: Presynaptic NMDARs on spinal nociceptor terminals state-dependently modulate synaptic transmission and pain
Source: Nat Commun. 2022 Feb 7;13:728. doi: 10.1038/s41467-022-28429-y (PMC8821657; doi:10.1038/s41467-022-28429-y)
Supplement: Supplementary file 4 — Source Data [file 41467_2022_28429_MOESM4_ESM.zip › NCOMMS-20-19861C Source Data/NCOMMS-20-19861C update Full unedited gel for Figures.pptx]

## Slide 1
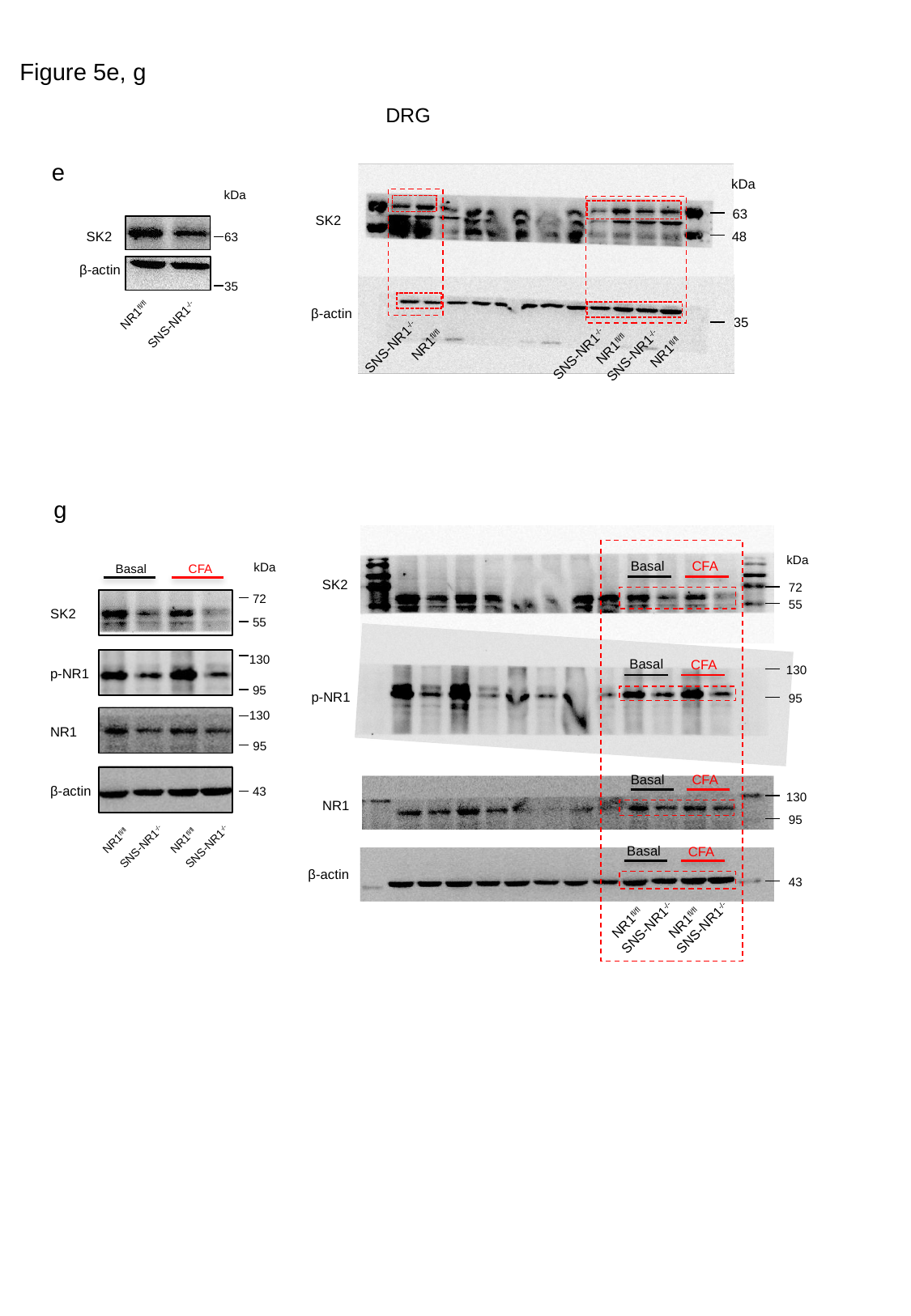

Figure 5e, g
DRG
e
kDa
63
SK2
48
β-actin
35
NR1fl/fl
SNS-NR1-/-
kDa
SK2
β-actin
NR1fl/fl
SNS-NR1-/-
63
35
NR1fl/fl
SNS-NR1-/-
NR1fl/fl
SNS-NR1-/-
g
kDa
Basal
CFA
kDa
Basal
CFA
SK2
72
72
55
SK2
55
130
Basal
CFA
130
p-NR1
95
p-NR1
95
130
NR1
95
Basal
CFA
β-actin
43
130
NR1
95
NR1fl/fl
NR1fl/fl
Basal
CFA
SNS-NR1-/-
SNS-NR1-/-
β-actin
43
NR1fl/fl
NR1fl/fl
SNS-NR1-/-
SNS-NR1-/-

## Slide 2
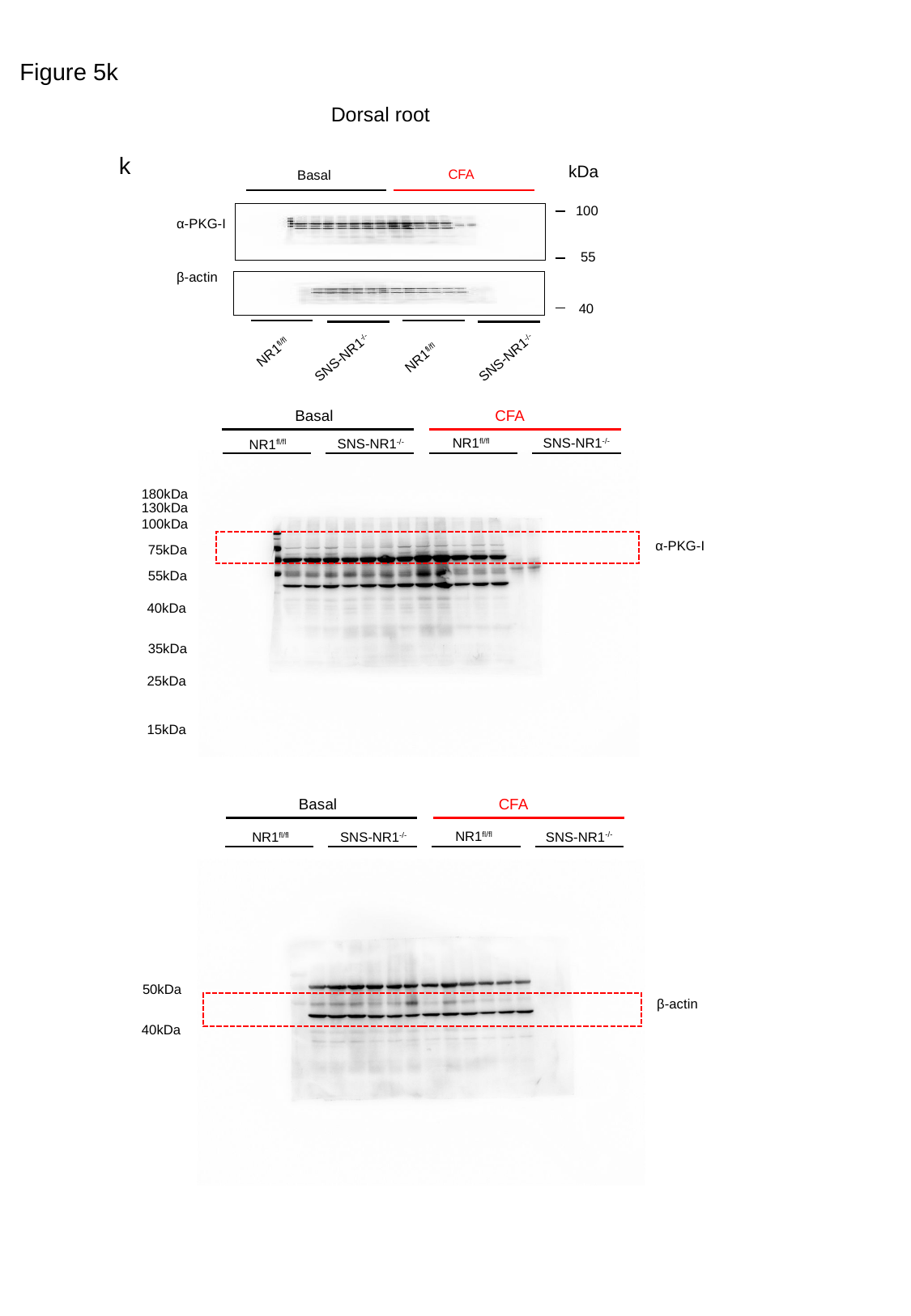

Figure 5k
Dorsal root
k
kDa
CFA
Basal
100
α-PKG-I
55
β-actin
40
NR1fl/fl
NR1fl/fl
SNS-NR1-/-
SNS-NR1-/-
Basal
CFA
NR1fl/fl
SNS-NR1-/-
SNS-NR1-/-
NR1fl/fl
180kDa
130kDa
100kDa
α-PKG-I
75kDa
55kDa
40kDa
35kDa
25kDa
15kDa
Basal
CFA
NR1fl/fl
SNS-NR1-/-
SNS-NR1-/-
NR1fl/fl
50kDa
β-actin
40kDa

## Slide 3
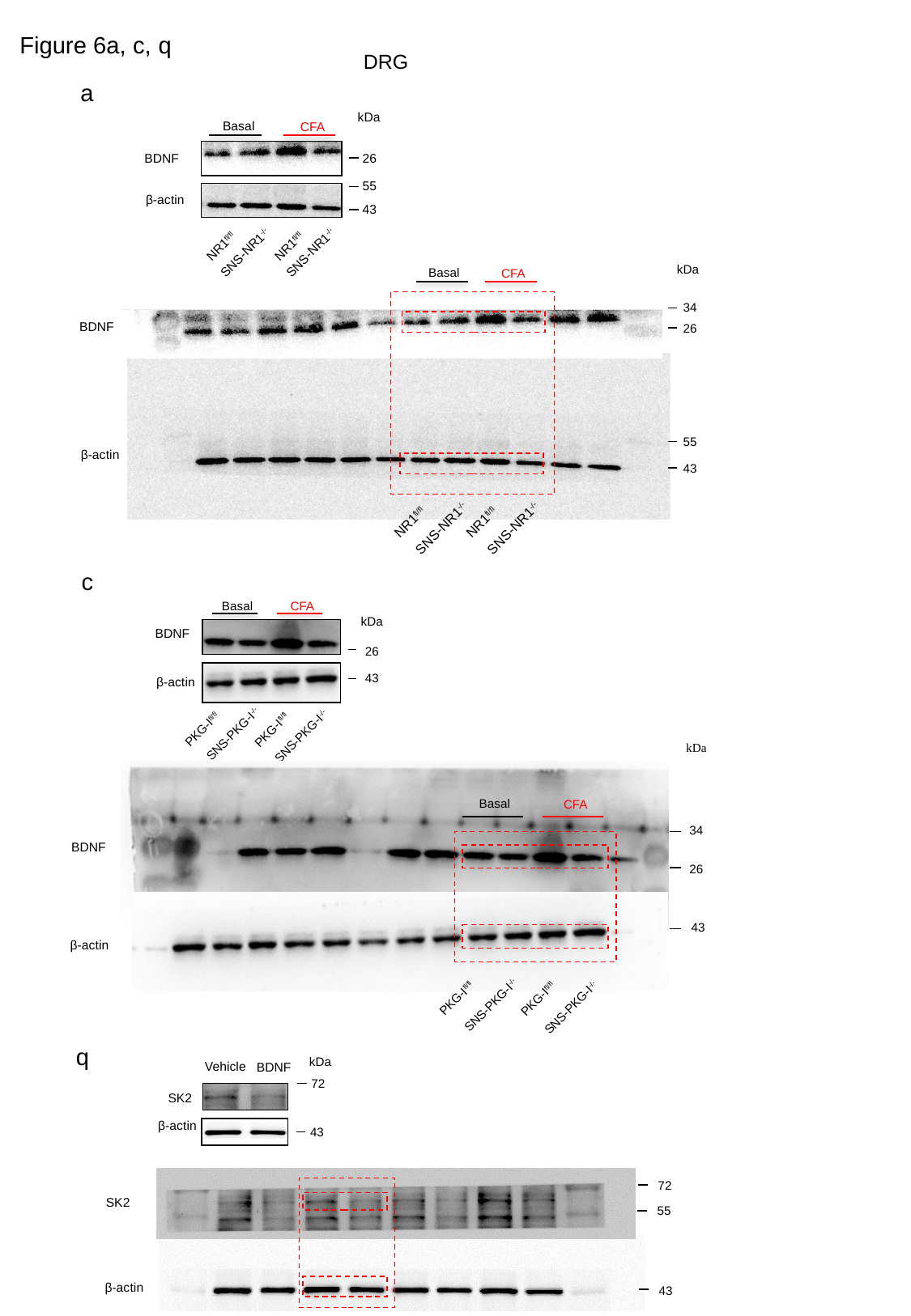

Figure 6a, c, q
DRG
a
kDa
Basal
CFA
26
BDNF
55
β-actin
43
NR1fl/fl
NR1fl/fl
SNS-NR1-/-
SNS-NR1-/-
kDa
Basal
CFA
34
BDNF
26
55
β-actin
43
NR1fl/fl
NR1fl/fl
SNS-NR1-/-
SNS-NR1-/-
c
Basal
CFA
kDa
BDNF
26
43
β-actin
SNS-PKG-I-/-
PKG-Ifl/fl
PKG-Ifl/fl
SNS-PKG-I-/-
kDa
Basal
CFA
34
BDNF
26
43
β-actin
PKG-Ifl/fl
PKG-Ifl/fl
SNS-PKG-I-/-
SNS-PKG-I-/-
q
kDa
Vehicle
BDNF
72
SK2
β-actin
43
72
SK2
55
β-actin
43

## Slide 4
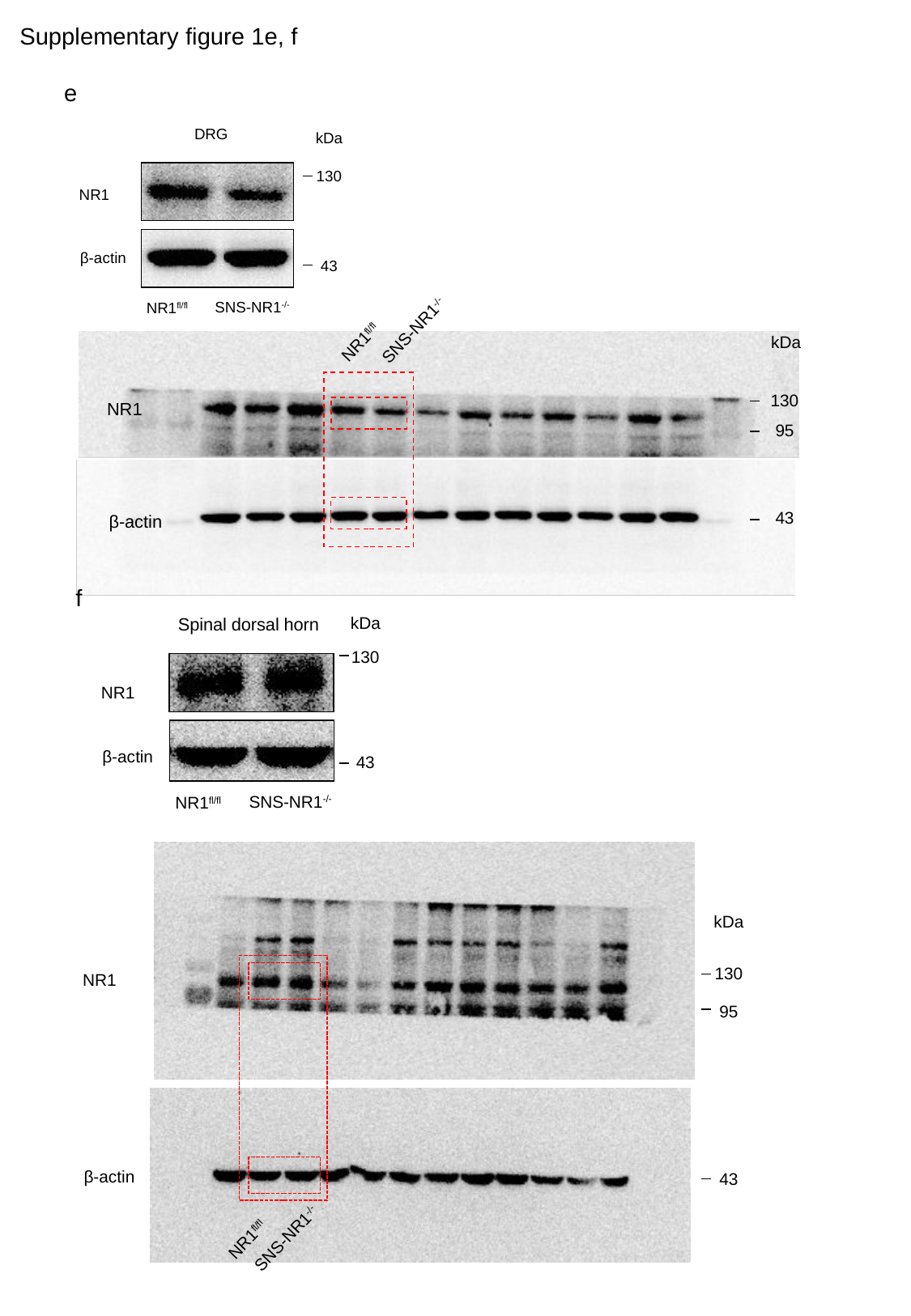

Supplementary figure 1e, f
e
DRG
kDa
130
NR1
β-actin
SNS-NR1-/-
NR1fl/fl
43
SNS-NR1-/-
kDa
NR1fl/fl
130
NR1
95
43
β-actin
f
Spinal dorsal horn
NR1
β-actin
SNS-NR1-/-
NR1fl/fl
kDa
130
43
kDa
130
NR1
95
β-actin
43
SNS-NR1-/-
NR1fl/fl

## Slide 5
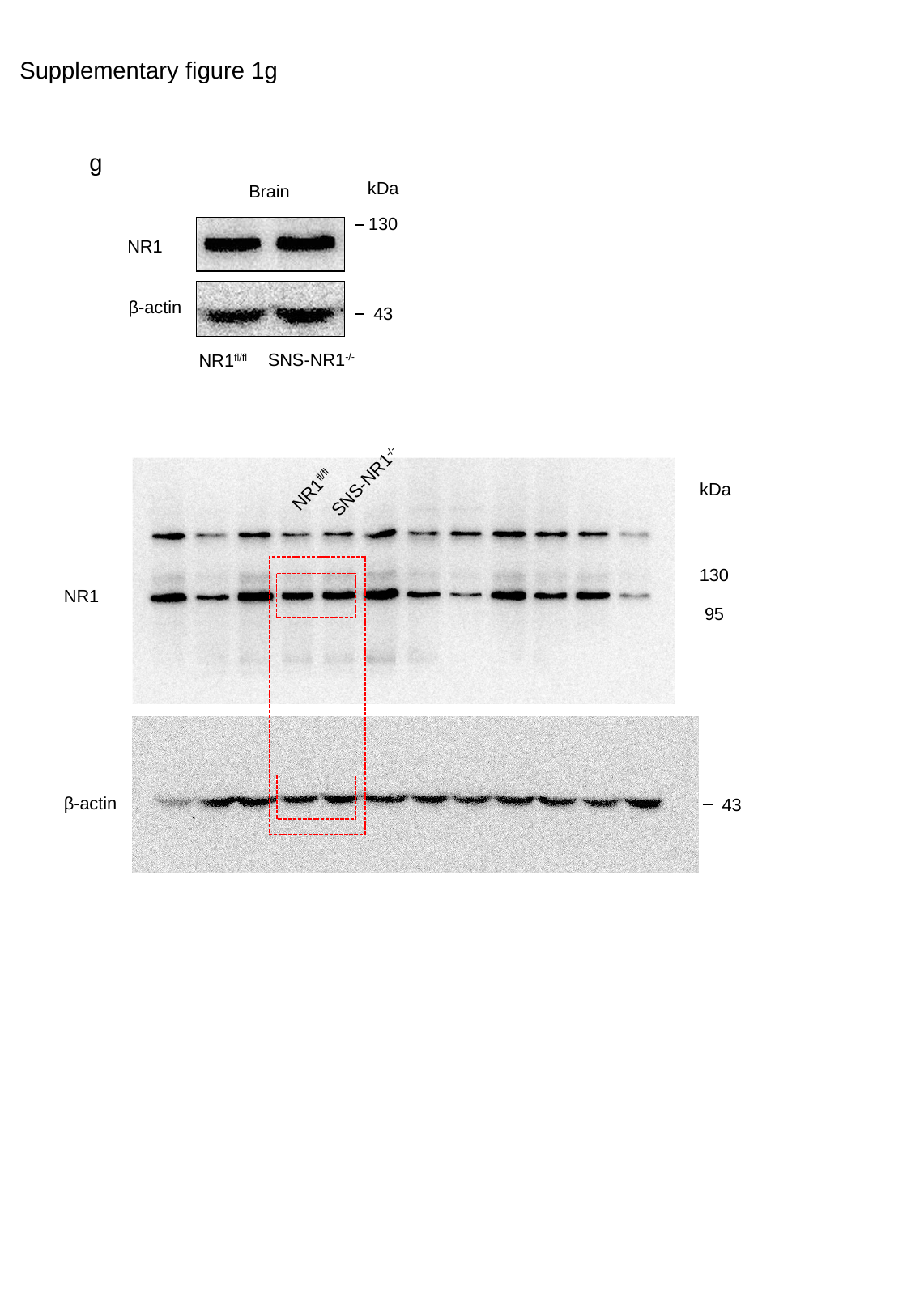

Supplementary figure 1g
g
kDa
Brain
130
NR1
β-actin
SNS-NR1-/-
NR1fl/fl
43
SNS-NR1-/-
kDa
NR1fl/fl
130
NR1
95
β-actin
43

## Slide 6
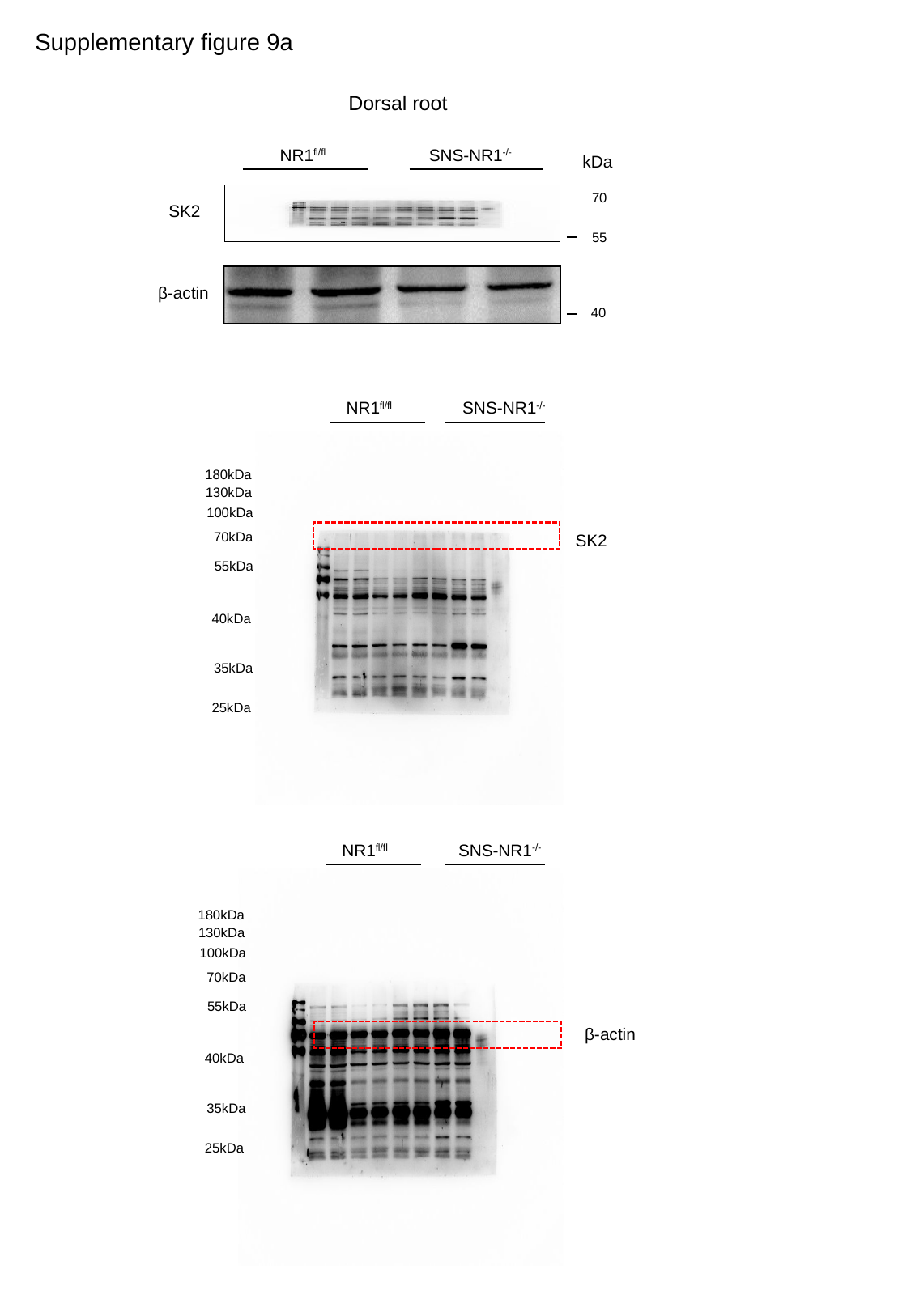

Supplementary figure 9a
Dorsal root
NR1fl/fl
SNS-NR1-/-
kDa
70
SK2
55
β-actin
40
NR1fl/fl
SNS-NR1-/-
180kDa
130kDa
100kDa
70kDa
SK2
55kDa
40kDa
35kDa
25kDa
NR1fl/fl
SNS-NR1-/-
180kDa
130kDa
100kDa
70kDa
55kDa
β-actin
40kDa
35kDa
25kDa

## Slide 7
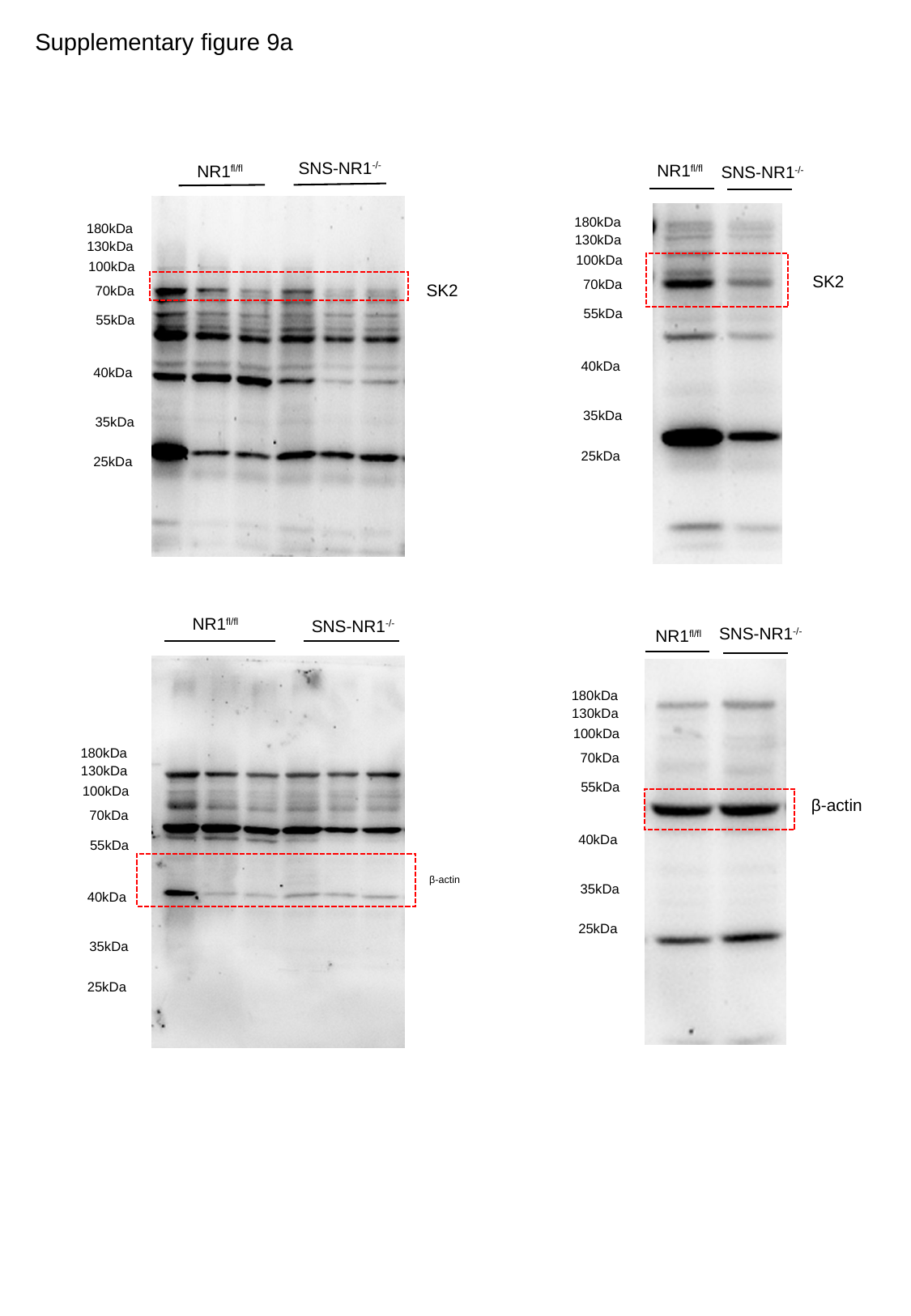

Supplementary figure 9a
SNS-NR1-/-
NR1fl/fl
NR1fl/fl
SNS-NR1-/-
180kDa
130kDa
100kDa
70kDa
55kDa
40kDa
35kDa
25kDa
180kDa
130kDa
100kDa
70kDa
55kDa
40kDa
35kDa
25kDa
SK2
SK2
NR1fl/fl
SNS-NR1-/-
SNS-NR1-/-
NR1fl/fl
180kDa
130kDa
100kDa
70kDa
55kDa
40kDa
35kDa
25kDa
180kDa
130kDa
100kDa
70kDa
55kDa
40kDa
35kDa
25kDa
β-actin
β-actin

## Slide 8
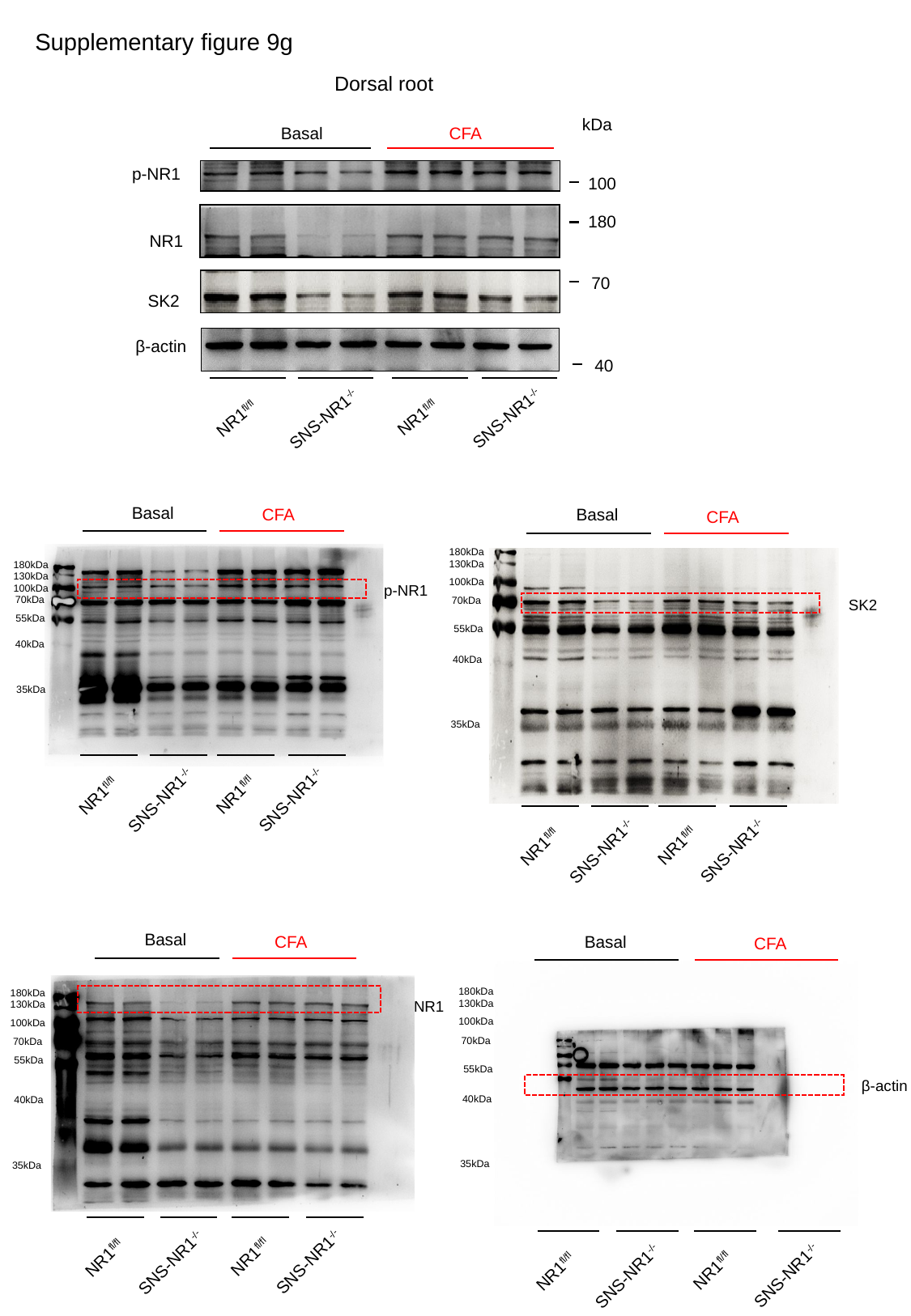

Supplementary figure 9g
Dorsal root
kDa
Basal
CFA
p-NR1
 100
180
NR1
70
SK2
β-actin
40
NR1fl/fl
SNS-NR1-/-
NR1fl/fl
SNS-NR1-/-
Basal
CFA
Basal
CFA
SK2
NR1fl/fl
NR1fl/fl
SNS-NR1-/-
SNS-NR1-/-
180kDa
130kDa
180kDa
130kDa
100kDa
p-NR1
100kDa
70kDa
70kDa
55kDa
55kDa
40kDa
40kDa
35kDa
35kDa
NR1fl/fl
NR1fl/fl
SNS-NR1-/-
SNS-NR1-/-
Basal
CFA
Basal
CFA
180kDa
180kDa
NR1
130kDa
130kDa
100kDa
100kDa
70kDa
70kDa
55kDa
55kDa
β-actin
40kDa
40kDa
35kDa
35kDa
NR1fl/fl
NR1fl/fl
SNS-NR1-/-
SNS-NR1-/-
NR1fl/fl
NR1fl/fl
SNS-NR1-/-
SNS-NR1-/-

## Slide 9
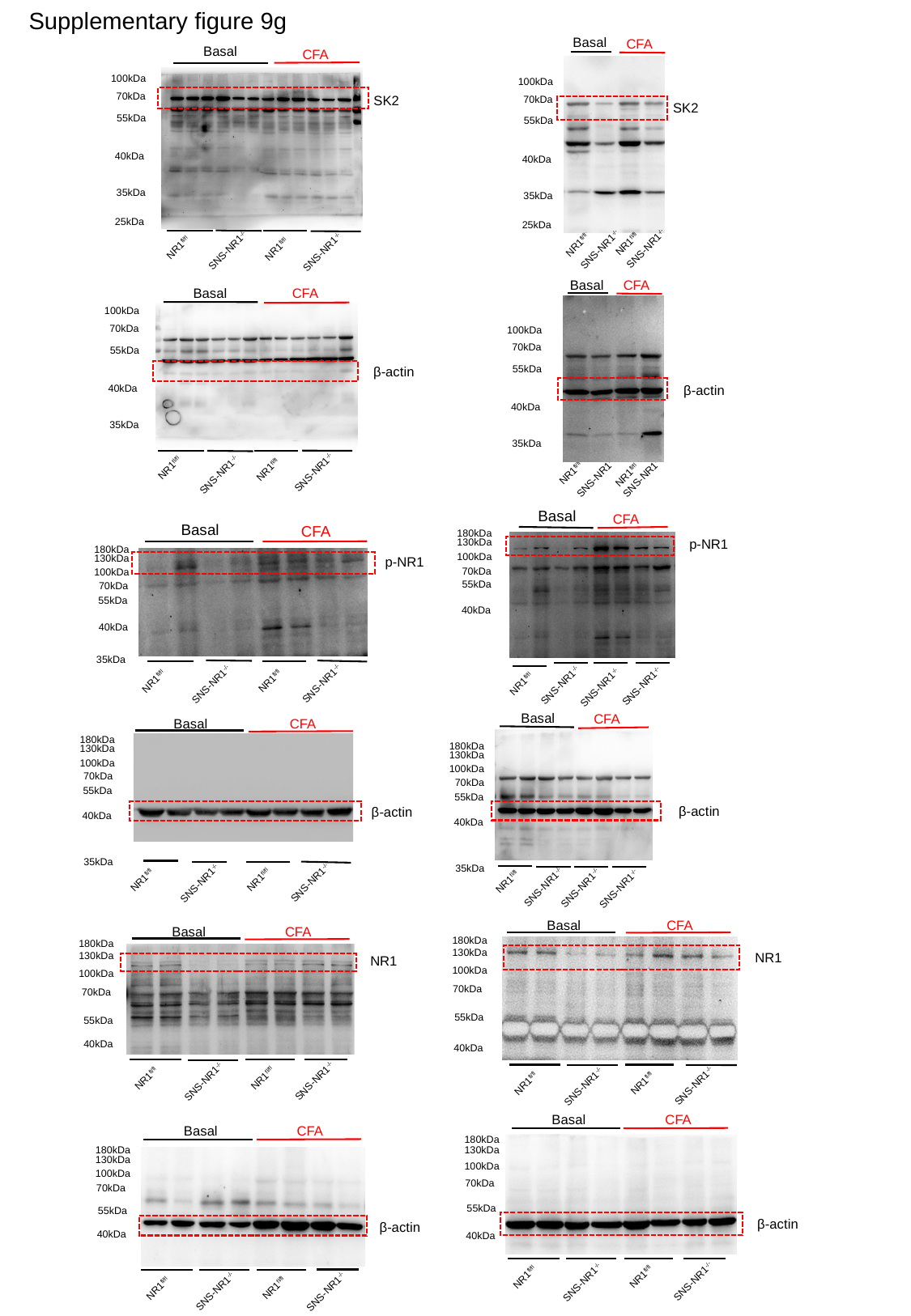

Supplementary figure 9g
Basal
CFA
NR1fl/fl
NR1fl/fl
SNS-NR1-/-
SNS-NR1-/-
100kDa
70kDa
55kDa
40kDa
35kDa
25kDa
SK2
Basal
CFA
100kDa
70kDa
55kDa
40kDa
35kDa
25kDa
SK2
NR1fl/fl
NR1fl/fl
SNS-NR1-/-
SNS-NR1-/-
Basal
CFA
NR1fl/fl
NR1fl/fl
SNS-NR1-/-
SNS-NR1-/-
Basal
CFA
100kDa
70kDa
55kDa
40kDa
35kDa
β-actin
NR1fl/fl
NR1fl/fl
SNS-NR1-/-
SNS-NR1-/-
100kDa
70kDa
55kDa
40kDa
35kDa
β-actin
Basal
CFA
180kDa
130kDa
100kDa
70kDa
55kDa
40kDa
p-NR1
NR1fl/fl
SNS-NR1-/-
SNS-NR1-/-
SNS-NR1-/-
Basal
CFA
180kDa
130kDa
p-NR1
100kDa
70kDa
55kDa
40kDa
35kDa
NR1fl/fl
NR1fl/fl
SNS-NR1-/-
SNS-NR1-/-
Basal
CFA
180kDa
130kDa
100kDa
70kDa
55kDa
40kDa
35kDa
β-actin
NR1fl/fl
SNS-NR1-/-
SNS-NR1-/-
SNS-NR1-/-
CFA
Basal
180kDa
130kDa
100kDa
70kDa
55kDa
β-actin
40kDa
35kDa
NR1fl/fl
NR1fl/fl
SNS-NR1-/-
SNS-NR1-/-
Basal
CFA
Basal
CFA
NR1fl/fl
NR1fl/fl
SNS-NR1-/-
SNS-NR1-/-
180kDa
130kDa
100kDa
70kDa
55kDa
40kDa
180kDa
130kDa
NR1
NR1
100kDa
70kDa
55kDa
40kDa
NR1fl/fl
NR1fl/fl
SNS-NR1-/-
SNS-NR1-/-
Basal
CFA
Basal
CFA
180kDa
130kDa
100kDa
70kDa
55kDa
40kDa
180kDa
130kDa
100kDa
70kDa
55kDa
40kDa
β-actin
β-actin
NR1fl/fl
NR1fl/fl
SNS-NR1-/-
SNS-NR1-/-
NR1fl/fl
NR1fl/fl
SNS-NR1-/-
SNS-NR1-/-

## Slide 10
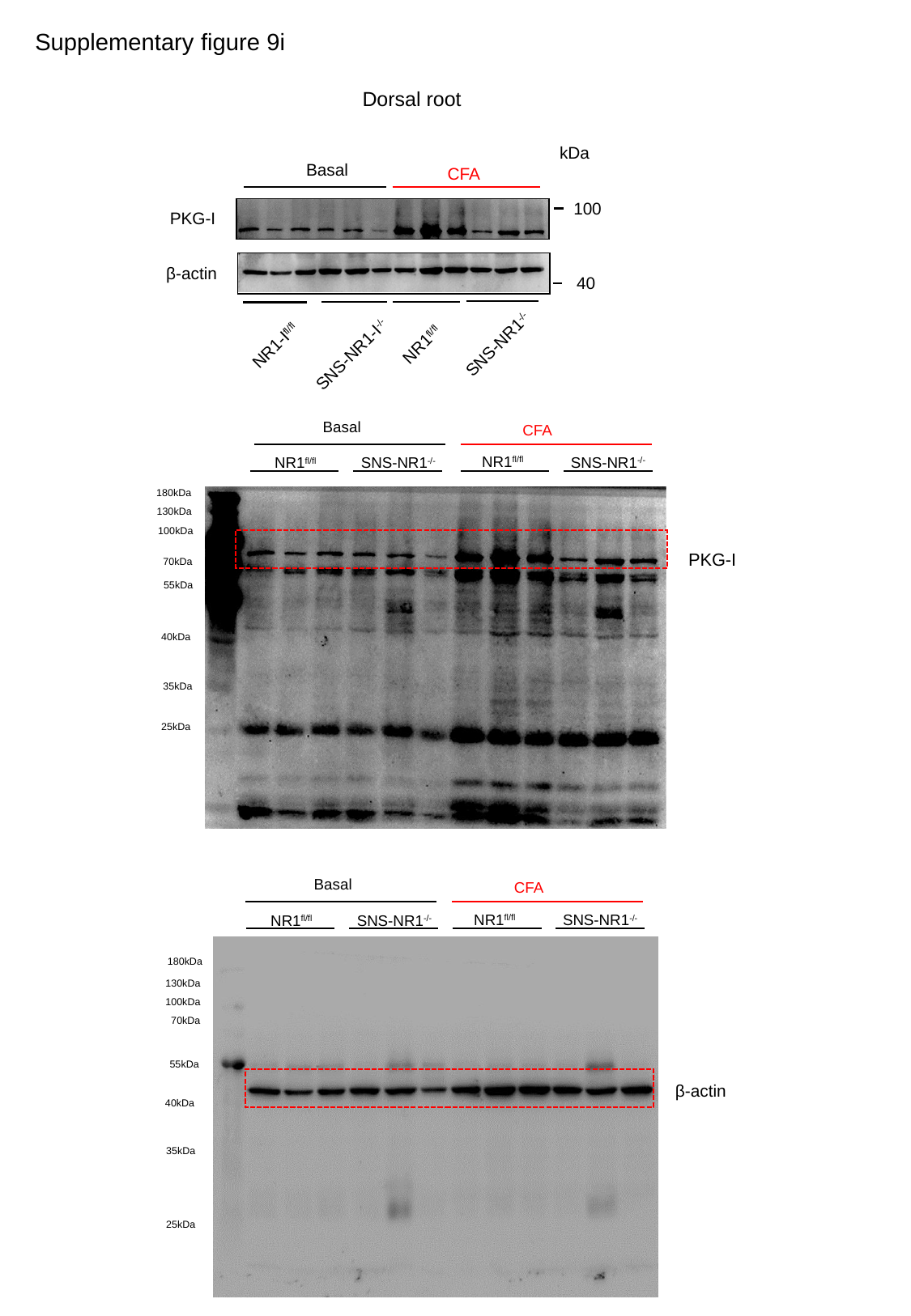

Supplementary figure 9i
Dorsal root
kDa
Basal
CFA
100
PKG-I
β-actin
40
SNS-NR1-/-
NR1fl/fl
NR1-Ifl/fl
SNS-NR1-I-/-
Basal
CFA
NR1fl/fl
SNS-NR1-/-
SNS-NR1-/-
NR1fl/fl
180kDa
130kDa
100kDa
PKG-I
70kDa
55kDa
40kDa
35kDa
25kDa
Basal
CFA
NR1fl/fl
SNS-NR1-/-
SNS-NR1-/-
NR1fl/fl
180kDa
130kDa
100kDa
70kDa
55kDa
β-actin
40kDa
35kDa
25kDa

## Slide 11
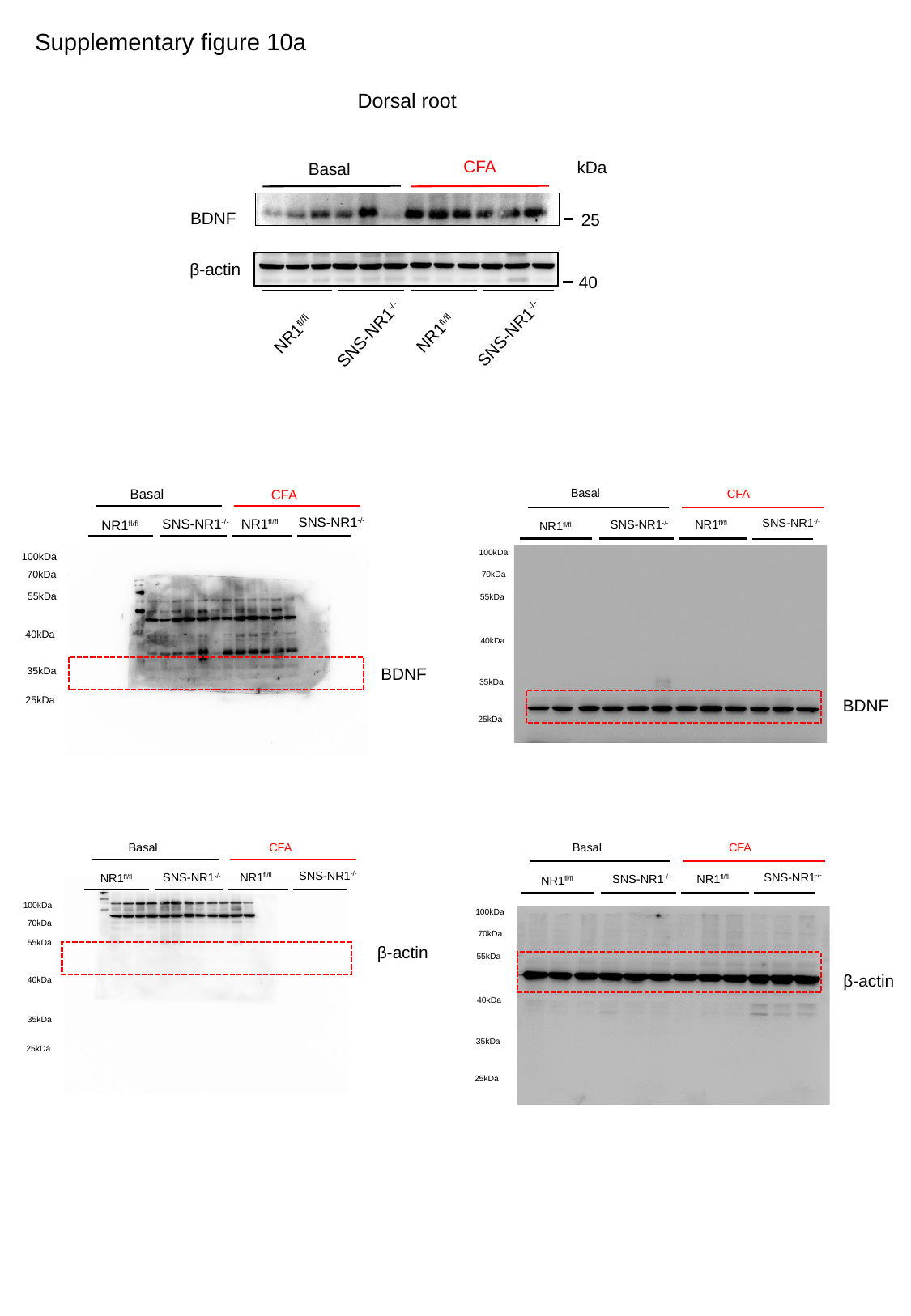

Supplementary figure 10a
Dorsal root
CFA
kDa
Basal
BDNF
25
β-actin
40
NR1fl/fl
SNS-NR1-/-
SNS-NR1-/-
NR1fl/fl
Basal
CFA
SNS-NR1-/-
NR1fl/fl
SNS-NR1-/-
NR1fl/fl
Basal
CFA
SNS-NR1-/-
NR1fl/fl
SNS-NR1-/-
NR1fl/fl
100kDa
70kDa
55kDa
40kDa
35kDa
25kDa
100kDa
70kDa
55kDa
40kDa
35kDa
25kDa
BDNF
BDNF
Basal
CFA
SNS-NR1-/-
NR1fl/fl
SNS-NR1-/-
NR1fl/fl
Basal
CFA
SNS-NR1-/-
NR1fl/fl
SNS-NR1-/-
NR1fl/fl
100kDa
70kDa
55kDa
40kDa
35kDa
25kDa
β-actin
100kDa
70kDa
55kDa
40kDa
35kDa
25kDa
β-actin
β-actin

## Slide 12
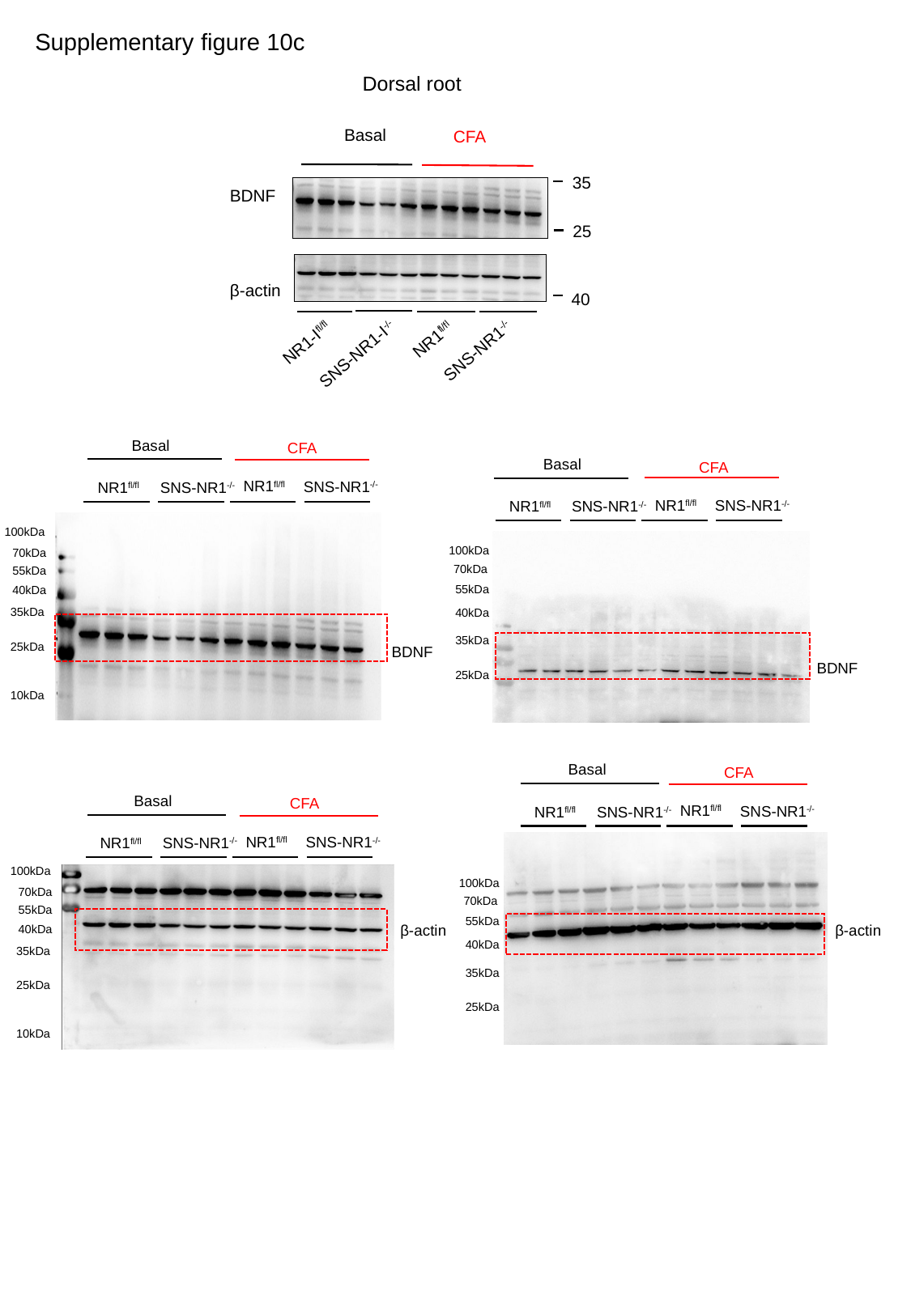

Supplementary figure 10c
Dorsal root
Basal
CFA
35
BDNF
25
β-actin
40
NR1fl/fl
NR1-Ifl/fl
SNS-NR1-/-
SNS-NR1-I-/-
Basal
CFA
NR1fl/fl
SNS-NR1-/-
SNS-NR1-/-
NR1fl/fl
Basal
CFA
NR1fl/fl
SNS-NR1-/-
SNS-NR1-/-
NR1fl/fl
100kDa
70kDa
55kDa
40kDa
35kDa
25kDa
10kDa
100kDa
70kDa
55kDa
40kDa
35kDa
25kDa
BDNF
BDNF
Basal
CFA
NR1fl/fl
SNS-NR1-/-
SNS-NR1-/-
NR1fl/fl
Basal
CFA
NR1fl/fl
SNS-NR1-/-
SNS-NR1-/-
NR1fl/fl
100kDa
70kDa
55kDa
40kDa
35kDa
25kDa
10kDa
100kDa
70kDa
55kDa
40kDa
35kDa
25kDa
β-actin
β-actin

## Slide 13
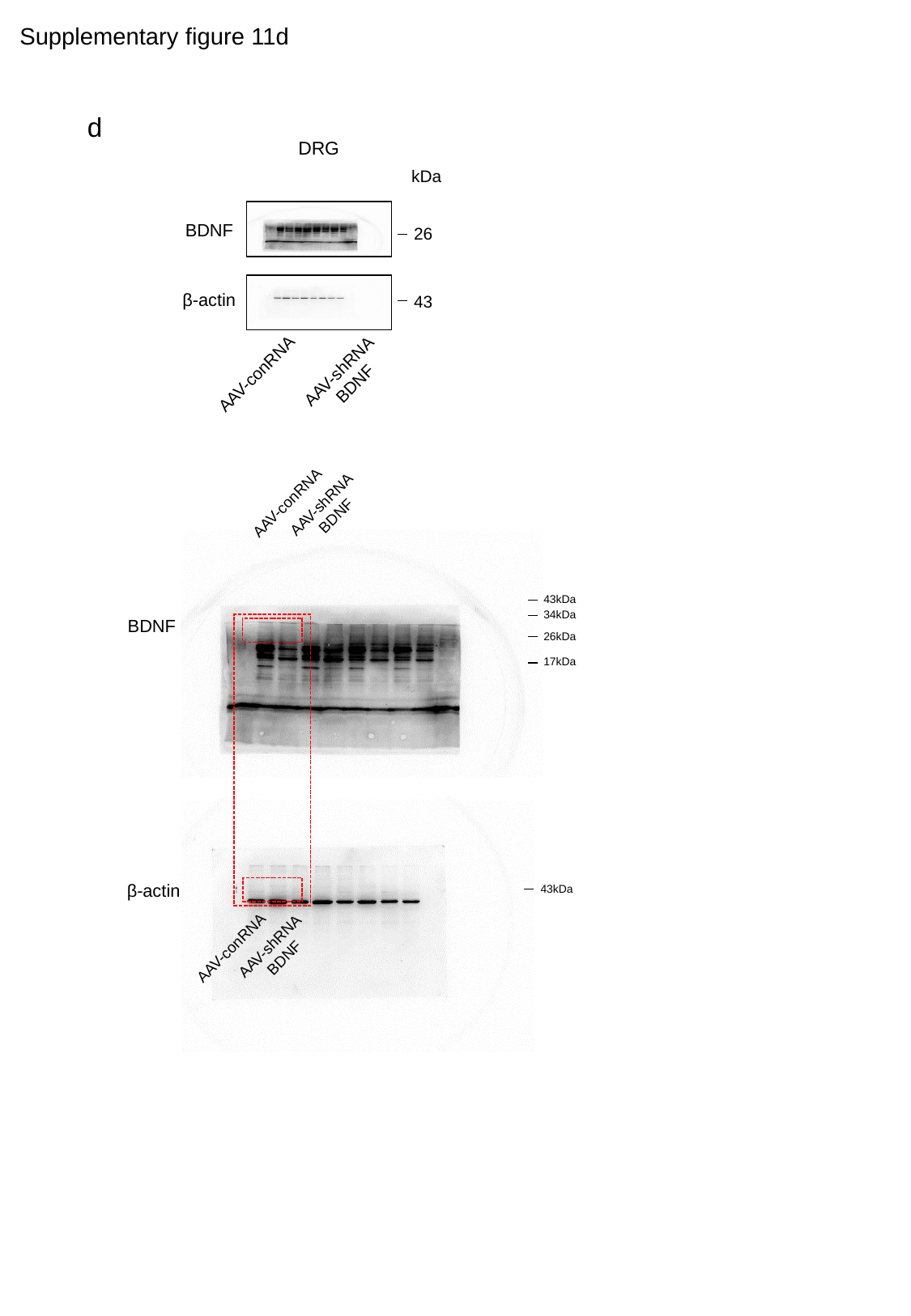

Supplementary figure 11d
d
BDNF
β-actin
AAV-shRNA
 BDNF
AAV-conRNA
DRG
kDa
26
43
AAV-shRNA
 BDNF
AAV-conRNA
43kDa
34kDa
BDNF
26kDa
17kDa
β-actin
43kDa
AAV-shRNA
 BDNF
AAV-conRNA

## Slide 14
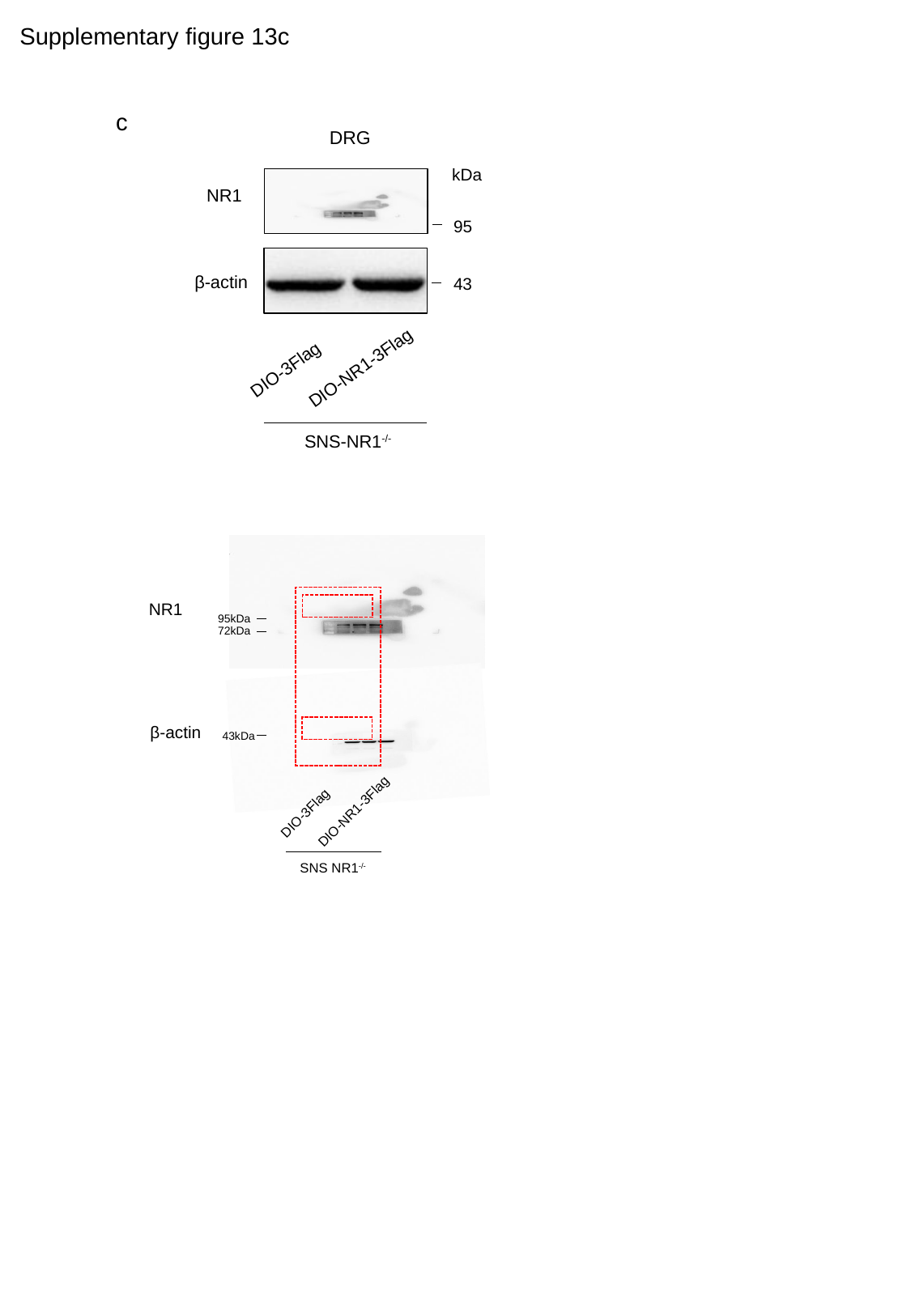

Supplementary figure 13c
c
DRG
kDa
NR1
95
β-actin
43
DIO-NR1-3Flag
DIO-3Flag
SNS-NR1-/-
NR1
95kDa
72kDa
β-actin
43kDa
DIO-NR1-3Flag
DIO-3Flag
SNS NR1-/-
